# Obesity, metabolic health status, and adverse outcomes in men and women

**DOI:** 10.1016/j.ajpc.2026.101556

**Published:** 2026-03-14

**Authors:** Roshan A Ananda, Bethlehem Solomon, Stephen J Nicholls, Kausik K Ray

**Affiliations:** a School of Public Health, Imperial College London, London, United Kingdom.; b Department of Primary Care and Public Health, Imperial College London, London, United Kingdom.; c Department of General Medicine, Box Hill Hospital, Melbourne, Victoria, Australia.; d Rock County Public Health, Wisconsin, United States.; e Victorian Heart Institute, Monash University, Victoria, Australia.

**Keywords:** Obesity, Metabolic health, Atherosclerotic cardiovascular disease, Heart failure, Cardiovascular risk, MASLD, Fatty liver, Renal disease, Prevention, UK Biobank

## Abstract

**Background:**

Metabolically healthy obesity account for approximately one-third of individuals with obesity and could affect up to 300million individuals worldwide. Whether this is a truly benign cardiometabolic phenotype as the name suggests is incompletely defined, leading to uncertainty regarding the optimal risk stratification and management strategies for these individuals.

**Objectives:**

To assess the sex-specific independent and joint associations of obesity and metabolic health status on cardiometabolic outcomes and death.

**Methods:**

A prospective cohort study of UK Biobank participants free from cardiovascular diseases and not underweight. Participants were divided by BMI into normal, overweight or obese, and the presence or absence of ≥1metabolic abnormality (hypertension, diabetes or dyslipidaemia). Exposures were assessed at baseline(2006-2010), with outcomes ascertained over a median follow-up of 12.9 years(IQR 12.6-13.3). Sex-specific outcomes were fatal or non-fatal atherosclerotic cardiovascular disease(ASCVD; a composite of coronary heart disease, ischaemic stroke, and peripheral artery disease), heart failure(HF), metabolic dysfunction-associated steatotic liver disease(MASLD), end-stage renal disease(ESRD) and all-cause mortality. Multivariable-adjusted cox regression models were used to estimate hazard ratio (HR) and 95 %CI.

**Results:**

Among 157,159 participants (mean age 56.5years [SD 8.2]; 55.6 % women), 24.2 % were obese and 68.2 % had ≥1 metabolic abnormality. Compared to normal BMI and no metabolic abnormality (reference group), obesity was associated with increased risk of ASCVD(HR 1.46, 95 %CI 1.24-1.73), HF(1.63,1.14-2.32), MASLD(2.37,1.22-4.61), all-cause mortality(1.36,1.10-1.69) but not ESRD in men *without* metabolic abnormalities, which increased when any metabolic abnormality was present: ASCVD(2.21,2.03-2.41), HF(2.91,2.41-3.50), MASLD(6.84,4.60-10.18), ESRD(5.42,2.94-10.02), and all-cause mortality(1.62,1.45-1.81). Corresponding risk from obesity in women *without* metabolic abnormalities were: ASCVD(1.34,1.14-1.58), HF(1.69,1.21-2.37), MASLD(4.44,3.00-6.59), and all-cause mortality(1.27,1.05-1.52) but not ESRD, which increased when metabolic abnormalities were present: ASCVD(2.51,2.30-2.74), HF(3.67,3.04-4.43), MASLD(8.17,6.13-10.89), ESRD(7.96,4.00-15.85) and all-cause mortality(1.67,1.51-1.85). Adverse outcomes increased with severity of obesity, the presence of central obesity, and with increasing numbers of metabolic abnormalities, with an effect modification by sex suggesting more harm from obesity, central obesity and metabolic abnormalities in women.

**Conclusions:**

Obesity *without* metabolic abnormalities is not benign and associated with multiple adverse cardiometabolic outcomes, further exacerbated when metabolic abnormalities occur. As 300million individuals may be considered metabolically healthy but obese, future studies should explore whether preventing or reversing obesity prior to the appearance of significant metabolic abnormalities results in improved health outcomes.

**Condensed Abstract:**

This prospective cohort study of over 1.9 million person-years follow-up demonstrates that obesity without metabolic abnormality increases the risk of ASCVD by 46 % in men and 34 % in women respectively, HF by 63 % and 69 %, MASLD by 137 % and 344 % and all-cause mortality by 36 % and 27 %, compared to those with normal BMI, with graded increase as severity of obesity increases. The presence of metabolic abnormality doubles event rates associated with obesity, with risk increasing with number of metabolic abnormalities present. These findings suggest that obesity *without* metabolic abnormality is not benign and support efforts to prevent or reverse obesity before overt metabolic dysfunction manifests.

## Introduction

Obesity rates have doubled since 1990 with nearly two-thirds of adults expected to be overweight or obese by 2050 [1]. Several cardiometabolic health outcomes and deaths[[2], [3], [4], [5], [6], [7], [8], [9]] are attributable to obesity, but whether these result from obesity *per se* or from casual metabolic risk factors resulting from or which cluster with obesity[10] is less certain. Metabolic risk factors are absent in one-third of individuals with obesity[11] and conflicting evidence about the independent effects of obesity[7,8,[12], [13], [14], [15], [16]] have led to the term *“metabolically healthy obesity”*, inferring a more benign state. Globally, this group accounts for up to 300 million individuals among an estimated 837 million people living with obesity in 2021, a figure projected to rise to 2 billion by 2050 [1]. Evidence from weight loss trials increasingly demonstrate that obesity is a modifiable risk factor for multiple disease outcomes with both weight dependent and independent mechanisms [17,18]. Thus, improved understanding of the relationship between obesity alone, or when it coexists with metabolic abnormalities across a spectrum of health outcomes[19] could help inform future public health strategies and the design of therapeutic trials.

Previously, we had explored the adverse effects of obesity and metabolic abnormalities on arterial stiffness[20], as elevated arterial stiffness is a strong predictor of future cardiovascular (CV) events and all-cause mortality[21]. In that study, we observed that arterial stiffness was increased among metabolically healthy obese individuals and was similar to that observed among metabolically unhealthy individuals with a normal BMI and highest when both were present (metabolically unhealthy obese) [20]. This provided the rationale to extend our observation in the same study population to prospectively evaluate the sex-specific independent and joint effects of obesity and metabolic health on fatal and non-fatal cardiometabolic outcomes and mortality. We hypothesised that obesity without metabolic abnormalities represents a transitional state in both men and women and is associated with higher cardiometabolic risk which increases further when metabolic dysfunction is present.

## Methods

### Study design

Details of UK Biobank are described in the Appendix. Participants with measurements of both BMI and arterial stiffness index (ASI) at baseline (n=169,529) were eligible (Flow Diagram Fig. S1). Further exclusion of underweight individuals (<18.5kg/m^2^), extreme ASI outliers, prevalent atherosclerotic cardiovascular disease (ASCVD) or heart failure (HF), provided a dataset for cardiovascular and mortality outcomes (*n* = 157,159). Individuals with pre-existing liver disease, defined as metabolic dysfunction-associated steatotic liver disease (MASLD), metabolic dysfunction-associated steatohepatitis (MASH) or liver cirrhosis were further excluded for liver outcome analyses. Those with pre-existing renal disease, defined as chronic kidney disease (CKD) stage 5 or baseline eGFR < 15 mL/min/1.73m², dialysis or renal transplant were excluded for renal outcome analyses. UK Biobank was approved by the North-West Multi-centre Ethics Committee. All participants provided written informed consent, complying with the Helsinki Declaration.

### Exposures

Exposures of interest were assessed at baseline (2006-2010), and their classifications are shown in the Appendix. Briefly, obesity was defined by BMI using WHO criteria: normal (18.5–24.9kg/m^2^), overweight (25.0–29.9kg/m^2^), and obese (≥30.0kg/m^2^), further classified by severity into class I (30.0–34.9kg/m^2^), II (35.0–39.9kg/m^2^) and III (≥40.0kg/m^2^). Central obesity was defined using WHO criteria: waist circumference ≥102cm for men and ≥88cm for women. BMI offers several advantages as a simple and reproducible surrogate for adiposity at the population-level, and is also routinely and systematically measured in clinical practice. BMI and waist circumference are highly correlated, but not interchangeable. Here, measures of central obesity were incorporated to determine whether they provide incremental prognostic information, particularly among those with normal BMI or overweight. Participants were considered as metabolically unhealthy if ≥1 of the following were present: hypertension, dyslipidaemia or diabetes using prior definitions of metabolic health [[22], [23], [24]]. This definition was chosen specifically to provide a “cleaner” more robust metabolically healthy cohort compared with many other prior analyses. This justification was supported by analyses where the impact of the number of metabolic abnormalities present (metabolic burden) was assessed for a graded relationship with outcomes.

### Outcomes

Disease outcomes were ascertained by linkage to national death-registries, primary care and hospital admission data, with definitions based on International Classification of Diseases (ICD-10) and Read (v2/v3) codes (Table S1). Outcomes of interest were fatal or non-fatal first events involving the cardiovascular, hepatic and renal systems separately, as well as mortality. The principal cardiovascular outcome of interest was ASCVD, reflecting a composite of coronary heart disease (CHD), ischaemic strokes (IS) and peripheral arterial disease (PAD) with individual components assessed separately, as was heart failure (HF). The hepatic outcome was metabolic dysfunction-associated steatotic liver disease (MASLD), consisting of a composite of metabolic dysfunction-associated steatohepatitis (MASH) or liver cirrhosis resulting from MASLD or MASH. The renal outcome was end-stage renal disease (ESRD), which was a composite of chronic kidney disease (CKD) stage 5, dialysis, renal transplant, or death from CKD, and finally all-cause mortality. Components of the CHD composite of myocardial infarction (MI) and chronic coronary syndrome (CCS) were assessed separately. CV death, haemorrhagic strokes, and a composite of all strokes were also assessed.

### Statistical analyses

All analyses were conducted separately by sex given the known differences in body fat between men and women, with participants divided into 6 mutually exclusive groups, based on the presence or absence of metabolic abnormalities (metabolic health strata) and three BMI categories. Sex-specific analyses were performed to better understand biological differences in the impact of adiposity (given known differences in body fat composition between men and women) on cardiometabolic risk between men and women. Event rates were presented as events per 1,000 person-years.

Multivariable-adjusted cox regression adjusted for age at baseline, ethnicity, smoking status, and Townsend deprivation quintiles, with normal BMI without metabolic abnormality as the reference group were performed with hazard ratios (HRs) and 95 % confidence interval (CI) reported to assess independent effects of all other combinations against the reference group, with corresponding rates per 1000 per year presented alongside (Appendix). Proportional hazards assumptions were assessed using Schoenfeld residuals with no violations detected.

Causal inference for the role of adiposity and outcomes was assessed for consistency and coherence in several ways by assessing biological gradients for increasing adiposity using linear tests for trend within each metabolic strata and sex, by severity of obesity, and using alternative definitions of such as central obesity. Similarly, the impact of the number of metabolic abnormalities present was assessed within each BMI category. Tests for interaction on a multiplicate scale (log additive) were performed using Cox regression which included an interaction term and assessed effect modification between sexes for obesity, central obesity and metabolic abnormalities on outcomes (Appendix).

Several sensitivity analyses were performed (Appendix). Firstly, the main regression model was further adjusted for systolic blood pressure, HbA1c, history of diabetes, HDL-cholesterol, LDL-cholesterol, and triglyceride levels. As obesity corelates with several lifestyle factors also associated with outcomes, further sensitivity analyses included stepwise-adjustment for physical activity, alcohol intake and sleep duration, with further adjustment for elevated high-sensitivity CRP (hs-CRP; ≥2mg/L), elevated ASI (≥10m/s) and eGFR (for renal outcome only) with an exploratory mediation analyses for elevated hs-CRP given emerging data from weight loss trials that potential benefits of these therapies may relate to attenuating inflammation[17,18].

Complete case analyses were performed for all outcomes using R version 4.0.2. As the proportion of missing data was low (< 0.2 %), it was assumed to be at random and multiple imputation was not performed.

## Results

### Baseline characteristics

A total of 157,159 participants were included with a mean age of 56.5 (SD 8.5) years and 55.6 % (n=86,902) were women. Among participants, 42.6 % (n=66,886) were overweight and 24.2 % (n=38,037) obese, mostly of class I (17.4 %,n=27,323) with 33.6 % (n=52,805) having central obesity. Overall, 68.2 % (n=107,258) had ≥1 metabolic abnormality.

Baseline characteristics of men and women were presented in Table 1, Table 2. Men were more often current smokers, more physically active with higher alcohol intake (all p<0.001). Prevalence of obesity was similar between sexes, but fewer men had class II-III obesity (Tables S2-S3) or had central obesity. Metabolic abnormalities were more prevalent among men whilst elevated hs-CRP was less common (p<0.001;Tables S4-S7).

**Table 1:** Baseline characteristics for men.

Characteristics	All Men (n = 70,257)	Metabolically Healthy*			Metabolically Unhealthy*		
		Normal BMI (n = 7,637)	Overweight (n = 7,923)	Obesity (n = 1,670)	Normal BMI (n = 10,395)	Overweight (n = 26,965)	Obesity (n = 15,667)
Age, mean (SD)	56·6 (8·3)	53·8 (8·4)	53·4 (8·2)	52·7 (8·1)	58·0 (8·1)	57·7 (8·1)	57·1 (8·0)
Ethnicity†, n ( %)							
White	63782 (91·5)	6992 (92·3)	7191 (91·5)	1477 (89·0)	9259 (89·8)	24508 (91·5)	14355 (92·4)
Black	1757 (2·5)	156 (2·0)	275 (3·5)	99 (6·0)	195 (1·9)	610 (2·3)	422 (2·8)
Asian	2916 (4·2)	277 (3·7)	240 (3·0)	37 (2·2)	687 (6·7)	1198 (4·5)	477 (3·1)
Mixed	450 (0·6)	68 (0·9)	59 (0·8)	18 (1·0)	58 (0·6)	145 (0·5)	102 (0·7)
Others	809 (1·2)	82 (1·1)	98 (1·2)	28 (1·7)	106 (1·0)	312 (1·2)	183 (1·2)
Central obesity†, n ( %)	20739 (29·5)	14 (0·2)	896 (11·3)	1255 (75·1)	42 (0·4)	5428 (20·1)	13104 (83·6)
Education†, n ( %)							
Has college or university qualifications	25177 (35·8)	3740 (49·0)	3156 (39·8)	518 (31·1)	4220 (40·6)	9269 (34·4)	4274 (27·3)
Others	44956 (64·0)	3884 (50·9)	4754 (60·0)	1148 (68·7)	6156 (59·2)	17654 (65·5)	11360 (72·5)
Region of residences†, n ( %)							
Urban	60657 (86·3)	6224 (86·7)	6885 (86·9)	1454 (87·1)	8976 (86·3)	23077 (85·6)	13641 (87·1)
Regional and rural areas	8830 (12·6)	907 (11·9)	922 (11·6)	186 (11·1)	1319 (12·7)	3607 (13·4)	1889 (12·1)
Townsend deprivation quintiles†, n ( %)							
Q1 (least deprived)	14115 (20·1)	1495 (19·6)	1666 (21·1)	303 (18·2)	2134 (20·5)	5717 (21·2)	2800 (17·9)
Q2	14037 (20·0)	1481 (19·5)	1572 (19·9)	332 (19·9)	2083 (20·1)	5622 (20·9)	2947 (18·8)
Q3	13955 (19·9)	1426 (18·7)	1646 (20·8)	328 (19·7)	1970 (19·0)	5468 (20·3)	3117 (19·9)
Q4	14007 (20·0)	1611 (21·1)	1582 (20·0)	321 (19·2)	2126 (20·5)	5199 (19·3)	3168 (20·3)
Q5 (most deprived)	14025 (20·0)	1610 (21·1)	1443 (18·2)	383 (23·0)	2070 (19·9)	4917 (18·3)	3602 (23·0)
Smoking status†, n ( %)							
Never-smoker	35579 (50·6)	4520 (59·2)	4365 (55·1)	840 (50·3)	5704 (54·9)	13197 (48·9)	6953 (44·4)
Ex-smoker	25945 (36·9)	2060 (27·0)	2580 (32·6)	629 (37·7)	3172 (30·5)	10559 (39·2)	6945 (44·3)
Current smoker	8295 (11·8)	1017 (13·3)	934 (11·8)	190 (11·4)	1466 (14·1)	3037 (11·3)	1651 (10·5)
Physical activity†							
Summed MET minutes per week for all activity, mean (SD)	2871 (2953)	3112 (3032)	3075 (3021)	2897 (3070)	2998 (2984)	2863 (2926)	2561 (2860)
Sleep duration† (hours per day), mean (SD)	7·1 (1·1)	7·1 (1·0)	7·0 (1·0)	7·0 (1·1)	7·2 (1·1)	7·1 (1·1)	7·1 (1·2)
Alcohol intake frequency†, n ( %)							
Never	4515 (6·4)	531 (7·0)	445 (5·6)	110 (6·6)	762 (7·3)	1590 (5·9)	1077 (6·9)
Special occasion only	5462 (7·8)	535 (7·0)	506 (6·4)	139 (8·3)	802 (7·7)	1991 (7·4)	1489 (9·5)
One to three times a month	6421 (9·1)	702 (9·2)	746 (9·4)	199 (11·9)	848 (8·2)	2282 (8·5)	1644 (10·5)
Once or twice a week	17924 (25·5)	1965 (25·7)	2168 (27·4)	493 (29·5)	2366 (22·8)	6636 (24·6)	4296 (27·4)
Three or four times a week	17817 (25·4)	2022 (26·5)	2192 (27·7)	406 (24·3)	2525 (24·3)	7043 (26·1)	3629 (23·2)
Daily or almost daily	17875 (25·4)	1856 (24·3)	1841 (23·2)	317 (19·0)	3055 (29·4)	7338 (27·2)	3468 (22·1)
Hypertension, n ( %)	41401 (58·9)	-	-	-	7876 (75·8)	20624 (76·5)	12901 (82·3)
Diabetes, n ( %)	4385 (6·2)	-	-	-	489 (4·7)	1689 (6·3)	2207 (14·1)
Dyslipidaemia, n ( %)	34600 (49·2)	-	-	-	5307 (51·1)	17600 (65·3)	11693 (74·6)
Number of metabolic abnormalities, n ( %)							
0	17230 (24·5)	7637 (100)	7923 (100)	1670 (100)	-	-	-
1	28760 (40·9)	-	-	-	7371 (70·9)	15141 (56·1)	6248 (39·9)
2	21175 (30·2)	-	-	-	2771 (26·7)	10700 (39·7)	7704 (49·2)
3	3092 (4·4)	-	-	-	253 (2·4)	1124 (4·2)	1715 (10·9)
Prevalence of arterial stiffness (≥ 10m/s), n ( %)	33303 (47·4)	2483 (32·5)	3095 (39·1)	704 (42·2)	4578 (44·0)	13908 (51·6)	8535 (54·5)

† Missing data: Ethnicity, education qualifications, smoking status, alcohol intake frequency (124; 0·2 %); Central obesity (9; 0·01 %); Region of residences (770; 1·1 %); Townsend deprivation quintiles (118; 0·2 %); Sleep duration (471; 0·7 %); Physical activity (10656; 15·2 %). *Metabolically healthy defined as the absence of hypertension, diabetes or dyslipidaemia.

⁎ Metabolically unhealthy defined as the presence of ≥1 hypertension, diabetes or dyslipidaemia.

**Table 2:** Baseline characteristics for woman.

Characteristics	All Women (n = 86,902)	Metabolically Healthy*			Metabolically Unhealthy*		
		Normal BMI (n = 18,417)	Overweight (n = 10,695)	Obesity (n = 3,559)	Normal BMI (n = 15,787)	Overweight (n = 21,303)	Obesity (n =17,141)
Age, mean (SD)	56·4 (8·1)	53·2 (7·9)	54·0 (7·9)	53·5 (7·7)	58·4 (7·6)	58·7 (7·5)	57·4 (7·8)
Ethnicity†, n ( %)							
White	78807 (91·2)	17139 (93·5)	9832 (92·4)	3090 (87·4)	14557 (92·7)	19143 (90·3)	15046 (88·4)
Black	2505 (2·9)	247 (1·4)	293 (2·8)	255 (7·2)	178 (1·1)	598 (2·8)	934 (5·5)
Asian	3114 (3·6)	530 (2·9)	269 (2·5)	72 (2·0)	663 (4·2)	972 (4·6)	608 (3·6)
Mixed	769 (0·9)	207 (1·1)	106 (1·0)	40 (1·2)	123 (0·8)	156 (0·7)	137 (0·8)
Others	1232 (1·4)	209 (1·1)	138 (1·3)	77 (2·2)	189 (1·2)	326 (1·6)	293 (1·7)
Central obesity†, n ( %)	32066 (36·9)	370 (2·0)	2983 (27·9)	3014 (84·7)	757 (4·8)	8944 (42·0)	15998 (93·3)
Education†, n ( %)							
Has college or university qualifications	28282 (32·5)	8099 (44·0)	3773 (35·3)	1121 (31·5)	5395 (34·2)	5718 (26·8)	4176 (24·4)
Others	58476 (67·3)	10290 (55·9)	6905 (64·6)	2431 (68·3)	10375 (65·7)	15551 (73·0)	12924 (75·4)
Region of residences†, n ( %)							
Urban	75238 (86·6)	15935 (86·5)	9242 (86·4)	3129 (87·9)	13435 (85·1)	18358 (86·2)	15139 (88·3)
Regional and rural areas	10862 (12·5)	2268 (12·3)	1329 (12·4)	389 (10·9)	2230 (14·1)	2782 (13·1)	1864 (10·9)
Townsend deprivation quintiles†, n ( %)							
Q1 (least deprived)	17380 (20·0)	3894 (21·2)	2208 (20·7)	570 (16·0)	3589 (22·8)	4371 (20·6)	2748 (16·1)
Q2	17365 (20·0)	3765 (20·5)	2175 (20·4)	624 (17·6)	3437 (21·8)	4399 (20·7)	2965 (17·3)
Q3	17318 (20·0)	3778 (20·5)	2117 (19·8)	691 (19·5)	3137 (19·9)	4387 (20·6)	3208 (18·7)
Q4	17403 (20·1)	3811 (20·7)	2192 (20·5)	715 (20·1)	3037 (19·3)	4107 (19·3)	3541 (20·7)
Q5 (most deprived)	17306 (19·9)	3143 (17·1)	1992 (18·6)	952 (26·8)	2557 (16·2)	4009 (18·8)	4653 (27·2)
Smoking status†, n ( %)							
Never-smoker	52358 (60·2)	11470 (62·3)	6312 (59·0)	2141 (60·2)	9581 (60·7)	12708 (59·7)	10146 (59·2)
Ex-smoker	26885 (30·9)	5346 (29·0)	3449 (32·2)	1120 (31·5)	4679 (29·6)	6786 (31·9)	5505 (32·1)
Current smoker	7159 (8·2)	1526 (8·3)	881 (8·2)	278 (7·8)	1461 (9·3)	1667 (7·8)	1346 (7·9)
Physical activity†							
Summed MET minutes per week for all activity, mean (SD)	2626 (2531)	2879 (2619)	2576 (2464)	2266 (2425)	2889 (2640)	2609 (2486)	2189 (2361)
Sleep duration† (hours per day), mean (SD)	7·2 (1·1)	7·2 (1·0)	7·2 (1·1)	7·1 (1·2)	7·2 (1·1)	7·2 (1·1)	7·1 (1·3)
Alcohol intake frequency†, n ( %)							
Never	8821 (10·2)	1288 (7·0)	800 (7·5)	352 (9·9)	1551 (9·8)	2356 (11·1)	2474 (14·4)
Special occasion only	13679 (15·7)	1975 (10·7)	1427 (13·3)	674 (18·9)	2092 (13·3)	3556 (16·7)	3955 (23·1)
One to three times a month	11434 (13·2)	2129 (11·6)	1412 (13·2)	594 (16·7)	1773 (11·2)	2736 (12·8)	2790 (16·3)
Once or twice a week	21597 (24·9)	4874 (26·5)	2925 (27·3)	934 (26·2)	3730 (23·6)	5229 (24·5)	3905 (22·8)
Three or four times a week	17284 (19·9)	4555 (24·7)	2378 (22·2)	592 (16·6)	3351 (21·2)	4066 (19·1)	2342 (13·7)
Daily or almost daily	13833 (15·9)	3551 (19·3)	1724 (16·1)	401 (11·3)	3251 (20·6)	3297 (15·5)	1609 (9·4)
Hypertension, n ( %)	41005 (47·2)	-	-	-	11795 (74·7)	15827 (74·3)	13383 (78·1)
Diabetes, n ( %)	3062 (3·5)	-	-	-	347 (2·2)	915 (4·3)	1800 (10·5)
Dyslipidaemia, n ( %)	31566 (36·3)	-	-	-	7060 (44·7)	12658 (59·4)	11848 (69·1)
Number of metabolic abnormalities*, n ( %)							
0	32671 (37·6)	18417 (100·0)	10695 (100·0)	3559 (100·0)	-	-	-
1	34911 (40·2)	-	-	-	12542 (79·4)	13783 (64·7)	8586 (50·1)
2	17238 (19·8)	-	-	-	3075 (19·5)	6943 (32·6)	7220 (42·1)
3	2082 (2·4)	-	-	-	170 (1·1)	577 (2·7)	1335 (7·8)
Prevalence of arterial stiffness (≥ 10m/s), n ( %)	26459 (30·4)	3815 (20·7)	2691 (25·2)	1025 (28·8)	4767 (30·2)	7628 (35·8)	6533 (38·1)

† Missing data: Ethnicity, education qualifications, smoking status, alcohol intake frequency (144; 0·2 %); Central obesity (20; 0·02 %); Region of residences (802; 0·9 %); Townsend deprivation quintiles (130; 0·2 %); Sleep duration (805; 0·9 %); Physical activity (18584; 21·4 %). *Metabolically healthy defined as the absence of hypertension, diabetes or dyslipidaemia.

⁎ Metabolically unhealthy defined as the presence of ≥1 hypertension, diabetes or dyslipidaemia.

Among the metabolically healthy, higher BMI category in both sexes was associated with less physical activity, higher social deprivation, lower educational attainment, worse arterial stiffness (Table 1, Table 2), modest differences in biomarkers of metabolic health but greater prevalence of inflammation (Tables S4-S5). The metabolically unhealthy were older and by definition had more risk factors, with the presence of obesity associated with the highest prevalence of arterial stiffness, inflammation, highest triglycerides and HbA1c (Tables S4-S5).

### Cardiovascular outcomes

**Fig. 1:**
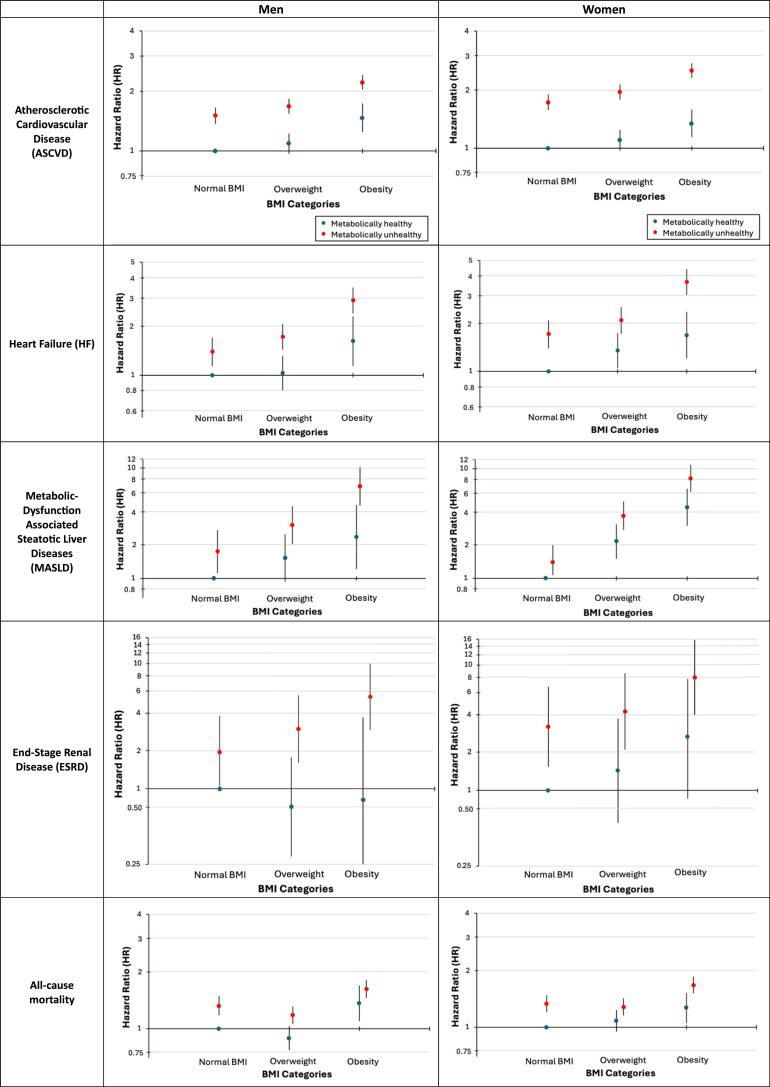
Relationship between increasing BMI and risk of ASCVD, Heart Failure, MASLD, ESRD, and all-cause mortality, in men and women by metabolic-health status. Metabolically unhealthy defined as presence any of hypertension, diabetes or dyslipidaemia. Metabolically healthy defined as the absence of any one of these. *Analyses adjusted for age, smoking status, ethnicity, and Townsend deprivation quintiles*. *Hazard ratios were plotted on log scales*.

During a median follow-up of 12.9 years (IQR 12.6-13.3), 10,507 (15.0 %) and 6,878 (7.9 %) ASCVD events occurred among men and women respectively. Among metabolically healthy men, rates per 1000 person-years for ASCVD increased from 6.37 (normal BMI) to 8.47 (obese) (HR 1.46,95 %CI 1.24-1.73), and among women from 2.91 to 4.00 (HR 1.34, 95 %CI 1.14-1.58;Fig. 1;Table 3; Table S8;P_Trend_<0.001 for each sex). Among the metabolically unhealthy, rates were approximately double, increasing in men from 12.27 (normal BMI) to 16.64 (obese) (HR 2.21,CI 2.03-2.41) and in women from 6.85 to 9.54 (HR 2.51, 95 %CI 2.30-2.74;Table S8); P_Trend_<0.001 for each sex. Individual components of the ASCVD composite endpoint and CV death (Fig. S2;Table S8) generally followed similar trends, except PAD which was only apparent among the metabolically unhealthy, with no association for haemorrhagic strokes (Fig. S3;Table S8).

**Table 3:** Summary comparing the absolute risk of cardiovascular, hepatic and renal outcomes across body mass index and metabolic health status categories.

	Event Rate (per 1,000 person-year)					
	Metabolically Healthy			Metabolically Unhealthy		
	Normal BMI	Overweight	Obesity	Normal BMI	Overweight	Obesity
ASCVD						
Men	6.37	6.67	8.47	12.27	13.19	16.64
Women	2.91	3.36	4.00	6.85	7.85	9.54
						
Heart Failure						
Men	1.28	1.26	1.90	2.82	3.24	5.23
Women	0.55	0.81	1.04	1.62	2.05	3.37
						
MASLD						
Men	0.27	0.40	0.60	0.58	0.95	2.16
Women	0.22	0.50	1.02	0.38	1.02	2.19
						
ESRD						
Men	0.11	0.08	0.09	0.30	0.44	0.78
Women	0.04	0.06	0.11	0.17	0.24	0.46
						
CV Death						
Men	0.92	0.77	1.23	2.16	2.02	3.03
Women	0.38	0.37	0.75	1.16	1.12	1.61
						
All-Cause Mortality						
Men	4.07	3.46	5.00	7.65	6.57	8.79
Women	2.39	2.75	3.18	4.87	4.74	5.68

Rates of HF per 1000 person-years increased with BMI from 1.28 (normal) to 1.90 (obese) in men(HR 1.63,95 %CI 1.14-2.32) and from 0.55 to 1.04 in women (HR1.69,95 %CI 1.21-2.37;Fig. 1;Table 3; Table S8;P_Trend_ 0.054 and <0.001 respectively) in the metabolically healthy, reaching 5.23 among obese men (HR 2.91,95 %CI 2.41-3.50) and 3.37 among obese women (3.67,95 %CI 3.04-4.43) who were metabolically unhealthy (Table S8;P_Trend_<0.001 for each sex).

### Hepatic, renal and mortality outcomes

Rates per 1000 person-years for MASLD increased from 0.27 (normal BMI) to 0.60 (obese) in men and from 0.22 to 1.02 in women (Fig. 1;Table 3; Table S11;P_Trend_ 0.004 and <0.001 respectively) among the metabolically healthy, increasing further among the metabolically unhealthy from 0.58 (normal BMI) to 2.16 (obese) for men (HR 6.84,95 %CI 4.60-10.18) and from 0.38 to 2.19 among women (HR 8.17,95 %CI 6.13-10.89;P_Trend_<0.001 for each sex). Increasing BMI was only associated with risk of ESRD among the metabolically unhealthy (Fig. 1;Table S14;P_Trend_<0.001 for each sex), with the highest risk seen among both obese men and women: HR 5.42 (95 %CI 2.94-10.02) and 7.96 (95 %CI 4.00-15.85).

Compared with a normal BMI, risk of all-cause mortality in metabolically healthy obese men and women were 1.36 (95 %CI 1.10-1.69) and 1.27 (95 %CI 1.05-1.52;Fig. 1;Table S15; P_Trend_ 0.096 and 0.009 respectively) which increased further among the metabolically unhealthy in men (HR 1.62,95 %CI 1.45-1.81) and women (HR 1.67,95 %CI 1.51-1.85; P_Trend_<0.001 for each sex).

### Severity of obesity and outcomes

**Fig. 2:**
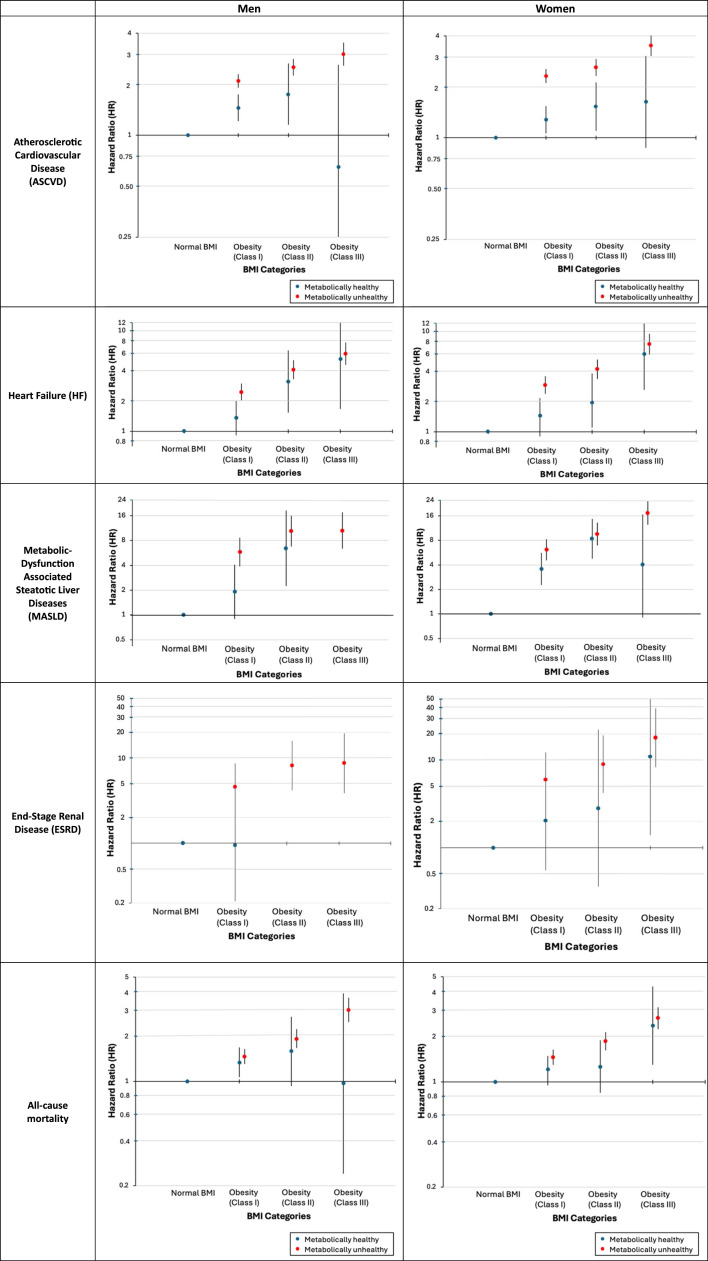
Relationship between severity of obesity among metabolically healthy or unhealthy individuals (presence or absence of any of hypertension, diabetes or dyslipidaemia) and risk of ASCVD, Heart Failure, MASLD, ESRD, and all-cause mortality in men and women. *Analyses adjusted for age, smoking status, ethnicity, and Townsend deprivation quintiles*. *Hazard ratios were plotted on log scales*.

Among the metabolically healthy, increasing severity of obesity was generally associated with higher risk of adverse outcomes in both sexes (Fig. 2). For instance, rates per 1000 person years for ASCVD increased from 8.35 (obesity class I) to 10.41 (class II) in men and from 3.86 to 4.52 in women respectively (Table S16) with too few men (n= 40/17,230, 0.2 %; Table 2) and women (n= 182/32,671, 0.5 %; Table S3) with class III obesity to allow meaningful assessment. Rates were higher still among the metabolically unhealthy, ranging from 15.91 (class I) to 21.21 (class III) in men and from 9.07 to 11.85 in women (P_Trend_<0.001 for each sex). Similar trends were observed for individual components of ASCVD (Fig. S4;Table S16), HF (Fig. 2;Table S16) and MASLD (Fig. 2; Table S17) within both metabolic health strata for each sex. However, the risk of ESRD (Fig. 2;Tables S18), and deaths (Fig. 2 and S4; Table S16 and S19) with increasing severity of obesity were only observed among the metabolically unhealthy and highest for class III obesity.

### Central obesity

**Fig. 3:**
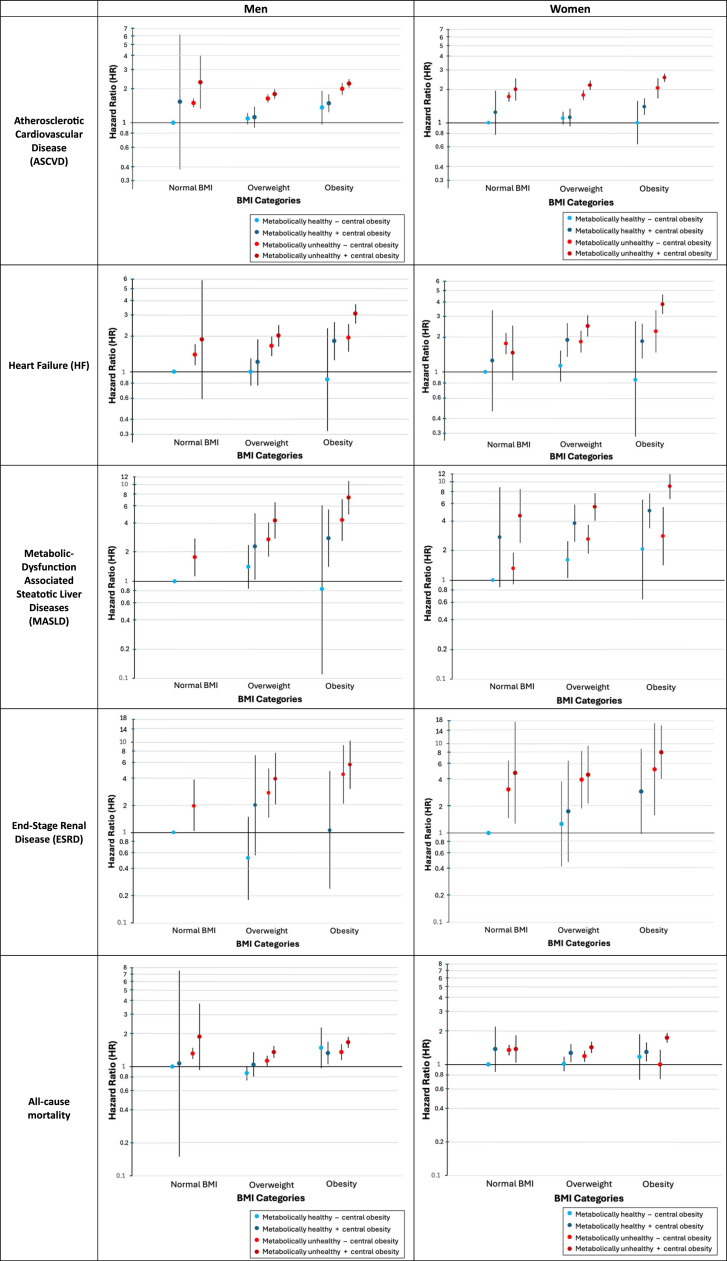
Relationship between the presence of central obesity within different categories of BMI and risk of ASCVD, Heart Failure, MASLD, ESRD, and all-cause mortality, in men and women by metabolic health status. Central obesity was defined as having a waist circumference of ≥102cm for men and ≥88cm for women. *Analyses adjusted for age, smoking status, ethnicity, and Townsend deprivation quintiles*. *Hazard ratios were plotted on log scales*.

In general, when central obesity was present, event rates tended to be higher for most outcomes in both men and women, and most apparent among those with normal BMI or overweight (Fig. 3 and S5;Tables S20-S23). These effects were largely independent in multivariable models (Tables S36-41). For instance, among the metabolically healthy with normal BMI, rates per 1000 person years for ASCVD increased from 6.36 (without central obesity) to 12.36 (with central obesity) in men and from 2.89 (without central obesity) to 4.01 (with central obesity; Table S20) in women, but had insufficient statistical power to demonstrate a significant difference. The effect of central obesity was better observed among the metabolically unhealthy. Among those with normal BMI, rates per 1000 person years for ASCVD increased from 12.21 (without central obesity) to 26.40 (with central obesity) in men and from 6.76 (without central obesity) to 8.68 (with central obesity; Table S20) in women, with corresponding risk of 1.50 (95 % CI 1.37-1.65) and 2.31 (95 % CI 1.33-4.00) in men, and 1.72 (95 % CI 1.56-1.89) and 2.01 (95 % CI 1.59-2.53) in women vs reference group. Similar trends were observed for individual components of ASCVD (Fig. S5;Table S20), HF (Fig. 3;Table S20), MASLD (Fig. 3;Table S21), ESRD (Fig. 3;Table S22) and all-cause mortality (Fig. 3;Table S23).

### Metabolic burden and outcomes

**Fig. 4:**
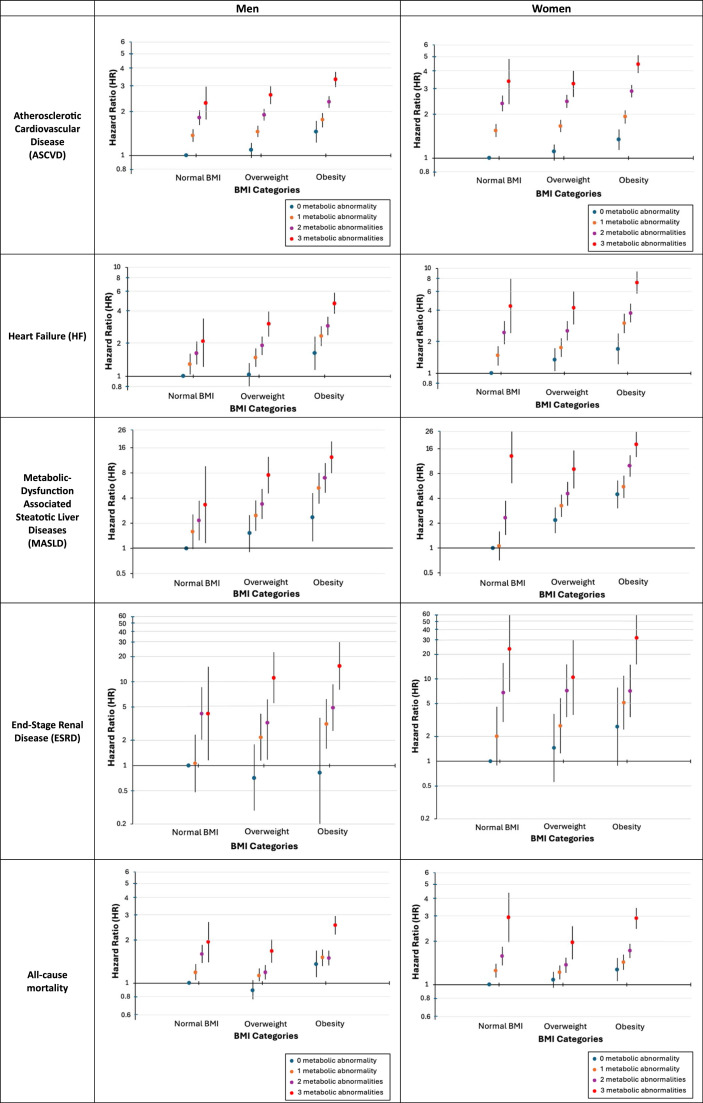
Relationship between the number of metabolic abnormalities present within different categories of BMI and risk of ASCVD, Heart Failure, MASLD, ESRD, and all-cause mortality in men and women. *Analyses adjusted for age, smoking status, ethnicity, and Townsend deprivation quintiles*. *Hazard ratios were plotted on log scales*.

The presence of increasing numbers of metabolic abnormalities generally associated with worse outcomes (Fig. 4); including ASCVD, its individual components (P_trend_<0.001;Fig. S6;Table S24), HF (Table S24), MASLD (Table S25), ESRD (Table S26), and mortality (Table S27) for both sexes, even when BMI was normal. Comparing extremes of exposure with outcomes (Table 4), rates per 1000 person-years associated with obesity and 3 metabolic abnormalities in men were for: ASCVD 27.98 vs 6.37, HF 10.25 vs 1.28, MASLD 4.30 vs 0.27, ESRD 2.51 vs 0.11 and all-cause mortality 15.96 vs 4.07 vs the metabolically healthy normal BMI group. Corresponding rates in women were for: ASCVD 18.52 vs 2.91, HF 8.14 vs 0.55, MASLD 4.99 vs 0.22, ESRD 2.06 vs 0.04 and all-cause mortality 10.92 vs 2.39.

**Table 4:** Summary comparing the risk of obesity with 3 metabolic abnormalities (most severe phenotype) on cardiovascular, hepatic and renal outcomes, to the reference group.

Outcomes	Men			Women		
	Normal BMI 0 metabolic abnormality (reference group)	Obesity 3 metabolic abnormalities		Normal BMI 0 metabolic abnormality (reference group)	Obesity 3 metabolic abnormalities	
	Event Rate (per 1,000 person-year)	Event Rate (per 1,000 person-year)	HR (95 % CI)	Event Rate (per 1,000 person-year)	Event Rate (per 1,000 person-year)	HR (95 % CI)
ASCVD	6.37	27.98	3.33 (2.96 – 3.75)	2.91	18.52	4.44 (3.86 – 5.11)
Heart failure	1.28	10.25	4.68 (3.75 – 5.85)	0.55	8.14	7.32 (5.73 – 9.36)
MASLD	0.27	4.30	12.35 (7.96 – 19.14)	0.22	4.99	18.09 (12.74 – 25.70)
ESRD	0.11	2.51	15.45 (8.05 – 29.65)	0.04	2.06	31.74 (15.03 – 67.04)
CV Death	0.92	6.89	4.62 (3.54 – 6.04)	0.38	4.45	6.74 (4.91 – 9.26)
All-Cause Mortality	4.07	15.96	2.55 (2.00 – 2.96)	2.39	10.92	2.90 (2.44 – 3.43)

^†^Adjusted for age, smoking, ethnicity, Townsend deprivation quintiles

^†^p < 0.001 for all outcomes vs reference group

ASCVD refers to atherosclerotic heart disease; MASLD refers to metabolic dysfunction-associated liver disease; ESRD refers to end-stage renal disease; CV death refers to cardiovascular death.

### Effect modification by sex

The effects of obesity and metabolic abnormalities were independent and largely additive (Tables S28-S43). However, among women, the adverse effects from obesity appeared multiplicative for CHD, IS, HF and MASLD (P_Intercation_<0.05;Tables S29-S33). Among individuals with normal BMI or overweight, presence of central obesity was not only independently associated with higher risk of all outcomes (Tables S36-S41), with evidence for multiplicative effects among women for ASCVD (Table S36) and MASLD (Table S38;P_Intercation_<0.05). Increasing number of metabolic abnormalities present were associated with multiplicative effects on ASCVD and HF in women (P_Intercation_<0.05;Tables S42-S43).

### Sensitivity and mediation analysis

Modest attenuation in risk was observed after adjusting for several CV risk factors (systolic BP, HbA1C, history of diabetes, HDL, LDL, and triglycerides) but the overall relationships remained significant (Fig. S7;Tables S44-S47). Further adjustment for lifestyle factors, elevated arterial stiffness, and baseline eGFR (for ESRD only) (Fig.s S8-S11;Tables S48-S55) provided further modest attenuation but associations remained. Adjustment for elevated hs-CRP (exploratory analysis) provided the strongest attenuation among sexes (Fig.s S8-S11;Tables S48-S55) with mediation analyses suggesting more prominent associations among women especially among the metabolically healthy (Fig.s S12-S15), mediating 48.5 % (women) and 28.0 % (men) of the observed association between obesity and ASCVD, compared to 15.6 % and 10.5 % respectively among the metabolically unhealthy. Other outcomes significantly mediated by hs-CRP among the metabolically healthy included HF and mortality while mediation of MASLD risk was seen only in women. No detectable mediation for ESRD was found for hs-CRP.

## Discussion

This study, with more than 1.9 million person-years of follow-up, has shown that obesity even without metabolic abnormalities is associated with a significantly higher risk of ASCVD, HF, MASLD and all-cause mortality in both sexes. When metabolic abnormalities occur, these risks increase further and include ESRD. These effects were largely independent and additive, increasing in a graded fashion both with increasing BMI category, severity of obesity and the number of metabolic abnormalities present. Although women had lower event rates, effect modification with greater harm from obesity, central obesity at lower BMI categories, and from presence of multiple metabolic abnormalities were observed.

Differences between this study and prior studies may relate to the definitions of “metabolically healthy obesity (MHO)” previously used, such as the absence of metabolic syndrome (≥3 metabolic abnormalities)[25] which may have attenuated differences between metabolically healthy and unhealthy individuals, inadvertently concluding that the former was more benign. Specifically, a previous analysis of the UK Biobank cohort[14] that employed a more stringent criteria[26] than the classical metabolic syndrome[25] reported weak associations between MHO phenotype with ASCVD and mortality outcomes, and that this risk was generally lower than among metabolically unhealthy non-obese counterparts. Furthermore, that study combined dichotomised BMI combining normal and overweight into one category (non-obese) as the reference group[14] attenuating potential differences between groups, versus the linear graded associations between increasing BMI, BMI severity and multiple outcomes in our study.

Our observation of independent and additive effects of obesity with any single metabolic abnormality, increasing further as the number of abnormalities present increased also support findings in some studies[[6], [7], [8]] that previously challenged the MHO concept. A large retrospective cohort study that utilised UK primary care electronic records (THIN)[7,8] had shown comparable findings to our study, whereby obesity without metabolic abnormality increased the risk of CHD by 49 %, stroke by 7 %, HF by 96 %, and CKD stage II or below by 66 %, but not PAD. By contrast, we reported on ESRD (hard renal outcomes) and found a linear association with increasing BMI only when metabolic abnormalities were present. Moreover, prior routine health record-based studies[7,8] did not adjust fully for lifestyle factors associated with obesity, nor assess the differential impact in men and women or assess whether associations varied by severity of obesity. External validation of our observations comes from the EPIC-CVD study[6], a prospective nested case-cohort of 10,474 individuals, which reported that obesity in the absence of any metabolic abnormality was associated with higher risk of CHD. However, EPIC-CVD did not study joint effects, sex-specific associations, nor outcomes beyond CHD [6].

The recent AHA PREVENT equations incorporate BMI for the prediction of heart failure, but not ASCVD citing limited evidence [27]. Initiation of preventive strategies for ASCVD may be delayed in obesity, relying on the manifestation of metabolic abnormalities which may only appear later from sustained obesity [5]. Our observations of a graded relationship with increasing BMI category and severity of obesity, consistent with recent studies[4,9], and merit further formal evaluation in multiple cohorts using measures of calibration, discrimination and net reclassification to assess whether measures of adiposity should be incorporated into risk prediction tools for primary prevention of ASCVD. The study by Dardari et al.[9] demonstrated that individuals with obesity class II and III had a significantly higher risk of adverse cardiovascular outcomes, with a greater magnitude of risk observed in women compared with men. However, the study[9] did not stratify participants according to metabolic health status, in contrast to our findings, which suggest that metabolically unhealthy phenotype further amplifies their cardiovascular risk across higher classes of obesity. Beyond obesity by BMI, our study suggests that women experience more cardiovascular risk associated with central obesity and metabolic abnormalities. Furthermore, consistent with prior findings[3], central obesity assessed using waist circumference identified individuals at higher ASCVD risk, particularly women, who by BMI would be considered normal or overweight, underscoring the incremental value of routine waist circumference measurement in this subgroup to capture visceral adiposity, which is currently underutilised in the real-world clinical practice. Given the higher rates of obesity in women worldwide,[1] the sex differences in body fat distribution and the greater relative harm observed from obesity and its associated risk factors in women, sex-specific risk equations incorporating measures of adiposity might prove useful in more precisely quantifying risk in women who already face disparities in cardiovascular outcomes. Such approaches may be particularly important for addressing existing sex disparities in cardiovascular outcomes, as traditional risk equations may underrecognize risk in women, especially those with obesity by BMI or excess visceral adiposity. While risk calculators evolve and formally assess the utility of measures of adiposity, it may be reasonable for now to incorporate obesity as part of shared decision-making for primary prevention before traditional obesity-related risk factors appear, analogous to discussions around existing risk enhancers which are not formally incorporated into risk engines.

In 2021, it was estimated that that 3.71 million deaths and 129 million disability-adjusted life-years(DALYs) were attributable to obesity,[28] with an economic cost projected to exceed 3.3 % global GDP by 2060 [29]. Furthermore, the rising prevalence of obesity among children and adolescents[30] often extending into adulthood[31] risks overwhelming health systems, hence, tackling obesity including its social determinants is a priority for policymakers worldwide [[32], [33], [34]]. We also observed that obesity was associated with higher social deprivation, lower educational attainment and less physical activity. Addressing these pose significant challenges, but several public health interventions show promise, including taxation of sugar-sweetened drinks[35], incentive-based health insurance schemes promoting healthy lifestyles[36,37], government-funded nutritious school meals[38], national child health[39], and school-based health literacy programs[40]. These represent cost-effective interventions for promoting healthy lifestyle habits and maintaining a healthy weight at a population level, with emerging highly effective weight loss pharmacotherapy[41,42] serving as potential adjuncts. Improved end-organ hard outcomes using these therapies in individuals with obesity and established ASCVD[17], HF with preserved ejection fraction[18,43], MASH or fibrosis[44,45], and CKD,[46] demonstrate the modifiability of multiple health outcomes resulting from obesity. However, enthusiasm for broad adoption of these medications should be balanced by considerations of access and costs[47], as cardiometabolic benefits may diminish after treatment discontinuation[48,49] with weight-regain of up to 60 % after 1-year [48] as well as unknown long-term adverse effects.

Previously, we observed an independent association between obesity and arterial stiffness [20]. However, elevated arterial stiffness did not mediate risk of ASCVD, suggesting that it was a marker rather than a causal factor. Obesity results in a chronic state of low-grade inflammation, with higher circulating levels of inflammatory cytokines [50]. In mediation analyses, inflammatory markers appeared to contribute to observed associations with several adverse outcomes which were stronger among women, especially when metabolic abnormalities were absent. Although healthy diet[51] and increased physical exercise[52] may attenuate inflammation, sensitivity analyses adjusting for these lifestyle factors did not attenuate the associations with elevated hs-CRP, suggesting potential direct links between adiposity and outcomes through inflammation. Of interest, GLP-1 agonists have been shown to reduce hs-CRP by 37.8 % before maximal weight loss is achieved, potentially contributing to observed CV benefits [17].

### Limitations

As with all studies, there are limitations. UK Biobank data may not be completely representative of the UK as only 5.5 % of those invited enrolled in the study, with potential healthy volunteer selection bias. That said, estimates of effect size were consistent with other UK population-representative cohorts [53]. This study represents a UK (mostly White) population, hence the implications for diverse global populations are uncertain and require validation in particular among populations where body fat distribution varies. Our analyses were restricted to those with ASI data rather than the whole UK Biobank cohort based on an a priori hypothesis, hence it is uncertain whether the results would materially change if extended to the whole cohort. We used a single cut off for waist circumference rather than ethnicity specific measures. Plasma insulin was not available in UK Biobank for the calculation of Homeostasis Model Assessment of Insulin Resistance (HOMA-IR), hence obesity-related insulin resistance could not be quantified. We excluded individuals with pre-existing liver diseases using ICD-10 and Read code diagnosis of MASLD, MASH or liver cirrhosis, rather than elevated AST or ALT, and did not account for baseline liver enzyme in liver outcome analysis. Medication use was incorporated into the exposure classification of hypertension, diabetes, and dyslipidaemia, but was not additionally adjusted for in the sensitivity analysis. The study is observational and whilst analyses adjusted for major confounders, residual confounding cannot be excluded. Several endpoints were assessed and some events were infrequent with relatively wide confidence intervals, hence these should be interpreted with appropriate caution. Furthermore, the duration and chronicity of obesity and metabolic dysfunction could not be determined in this study.

## Conclusions

The present study does not support the concept of “metabolically healthy obesity” as obesity without metabolic abnormality increases the risk of several adverse health outcomes, potentially reflecting a transition state towards more overt manifestation of cardiometabolic abnormalities which further increased risk. As 300 million individuals may be considered metabolically healthy but obese, future studies should explore whether preventing or reversing obesity prior to the appearance of significant metabolic abnormalities results in improved health outcomes.

## Data sharing

Data may be obtained from a third party and are not publicly available. This research was conducted using the UK Biobank resource under access application 104072. UK Biobank will make the data available to all bona fide researchers for all types of health-related research that is in the public interest, without preferential or exclusive access for any persons. All researchers will be subject to the same application process and approval criteria as specified by UK Biobank. For more details on the access procedure, see the UK Biobank website: http://www.ukbiobank.ac.uk/register-apply.

**Figure:**
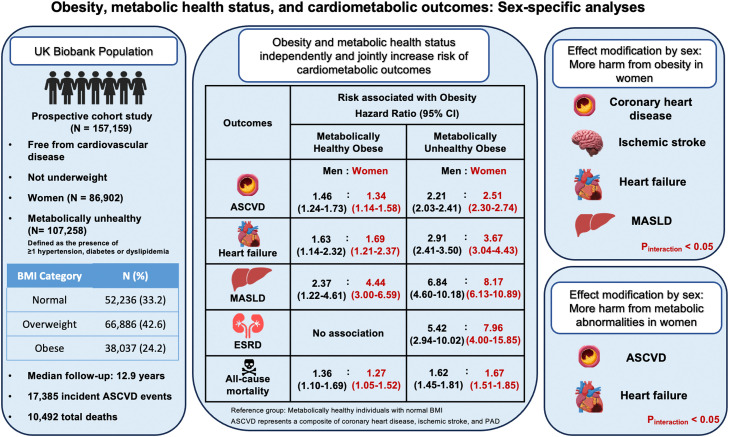
Central Illustration.

## CRediT authorship contribution statement

**Roshan A Ananda:** Writing – original draft, Visualization, Validation, Software, Methodology, Formal analysis, Data curation, Conceptualization. **Bethlehem Solomon:** Writing – review & editing, Methodology, Conceptualization. **Stephen J Nicholls:** Writing – review & editing, Supervision, Methodology, Conceptualization. **Kausik K Ray:** Writing – review & editing, Visualization, Validation, Supervision, Methodology, Conceptualization.

## Declaration of competing interest

SJN received grant/research support from AstraZeneca, NewAmsterdam Pharma, Amgen, Anthera, Cyclarity, Eli Lilly, Esperion, Novartis, Cerenis, The Medicines Company, Resverlogix, InfraReDx, Roche, Sanofi-Regeneron, and LipoScience; and was a consultant for Abcentra, AstraZeneca, Amarin, Akcea, Eli Lilly, Anthera, Omthera, Merck, Takeda, Resverlogix, Sanofi-Regeneron, CSL Behring, Esperion, Boehringer Ingelheim, Daiichi Sankyo, Scribe, Silence Therapeutics, CSL Seqirus and Vaxxinity. KKR reports unrestricted research grants (last 3 years) to Imperial College London, Amarin, Amgen, Sanofi, Regeneron, Daiichi Sankyo, and Ultragenix; Consulting fees from Novartis, Daiichi Sankyo, Kowa, Esperion, Novo Nordisk, MSD, Lilly, Silence Therapeutics, AZ, New Amsterdam Pharma, Bayer, Beren Therapeutics, CLEERLY, EMENDOBIO, SCRIBE, CRISPR, VAXXINITY, Amarin, Regeneron, Ultragenix, for serving as a member of the SC or EC of clinical trials and roles as PI, NLI, attending advisory boards; lecture fees from Novartis, BI, AZ, Novo Nordisk, Viatris, Amarin, Sanofi, Amgen, Esperion, Daiichi Sankyo, Macleod Pharma for CME and non-CME symposia at international meetings; stock options from New Amsterdam Pharma, SCRIBE and PEMI31. The other authors have no disclosures.

## Appendix Supplementary materials

Image, application 1
