# Supplementary material for: Obesity, metabolic health status, and adverse outcomes in men and women
Source: Am J Prev Cardiol. 2026 Mar 14;28:101556. doi: 10.1016/j.ajpc.2026.101556 (PMC13326122; doi:10.1016/j.ajpc.2026.101556)
Supplement: Supplementary file 1 [file mmc1.pdf]

Supplementary Appendix

Obesity, Metabolic Health Status, and Adverse Outcomes in Men and Women

Ananda RA, Solomon B, Nicholls SJ, Ray KK.

Table of Contents

Index	Page
**Supplemental Methods**	**5**
**Table S1** Ascertainment of cardiovascular, hepatic, and renal outcomes using International Classification of Disease (ICD)-10 codes.	**8**
**Table S2** Baseline body mass index for men, stratified by metabolic health status.	**9**
**Table S3** Baseline body mass index for women, stratified by metabolic health status.	**9**
**Table S4** Baseline cardiometabolic profile and medication use for men, excluding those pre-existing cardiovascular disease.	**10**
**Table S5** Baseline cardiometabolic profile of for women, excluding those pre-existing cardiovascular disease.	**11**
**Table S6** Distribution of medication uses for the control of metabolic health for men, excluding those pre-existing cardiovascular disease.	**12**
**Table S7** Distribution of medication uses for the control of metabolic health for women, excluding those pre-existing cardiovascular disease.	**13**
**Table S8** Relationship between BMI and metabolic health status (absence or presence of metabolic abnormalities) on cardiovascular outcomes, stratified by sex.	**14**
**Table S9** Baseline characteristics for men, excluding those pre-existing cardiovascular or liver disease.	**18**
**Table S10** Baseline characteristics for women, excluding those pre-existing cardiovascular or liver disease.	**20**
**Table S11** Relationship between BMI and metabolic health status on MASLD, stratified by sex.	**22**
**Table S12** Baseline characteristics for men, excluding those pre-existing cardiovascular or renal disease.	**23**
**Table S13** Baseline characteristics for women, excluding those with pre-existing cardiovascular or renal diseases.	**25**
**Table S14** Relationship between BMI and metabolic health status on end-stage renal disease, stratified by sex.	**27**
**Table S15** Relationship between BMI and metabolic health status on all-cause mortality, stratified by sex.	**28**
**Table S16** Relationship between obesity severity and metabolic health status on cardiovascular outcomes, stratified by sex.	**29**
**Table S17** Relationship between obesity severity and metabolic health status on MASLD, stratified by sex.	**33**
**Table S18** Relationship between obesity severity and metabolic health status on end-stage renal disease, stratified by sex.	**34**
**Table S19** Relationship between obesity severity and metabolic health status on all-cause mortality, stratified by sex.	**35**
**Table S20** Relationship between BMI, central obesity and metabolic health status on cardiovascular outcomes, stratified by sex.	**36**
**Table S21** Relationship between BMI, central obesity and metabolic health status on MASLD, stratified by sex.	**42**
**Table S22** Relationship between BMI, central obesity, and metabolic health status on end-stage renal disease, stratified by sex.	**43**
**Table S23** Relationship between BMI, central obesity, and metabolic health status on all-cause mortality, stratified by sex.	**44**
**Table S24** Relationship between BMI and the number of metabolic abnormalities on cardiovascular outcomes, stratified by sex.	**45**

**Table S25** Relationship between BMI and the number of metabolic abnormalities on MASLD, stratified by sex.	**52**
**Table S26** Relationship between BMI and the number of metabolic abnormalities on end-stage renal disease, stratified by sex.	**53**
**Table S27** Relationship between BMI and the number of metabolic abnormalities on all-cause mortality, stratified by sex.	**54**
**Table S28** Interaction between sex and obesity, and its effect on ASCVD.	**55**
**Table S29** Interaction between sex and obesity, and its effect on CHD.	**55**
**Table S30** Interaction between sex and obesity, and its effect on ischaemic stroke.	**56**
**Table S31** Interaction between sex and obesity, and its effect on HF.	**56**
**Table S32** Interaction between sex and obesity, and its effect on CV death.	**57**
**Table S33** Interaction between sex and obesity, and its effect on MASLD.	**57**
**Table S34** Interaction between sex and obesity, and its effect on ESRD.	**58**
**Table S35** Interaction between sex and obesity, and its effect on all-cause mortality.	**58**
**Table S36** Interaction between sex and central obesity, and its effect on ASCVD.	**59**
**Table S37** Interaction between sex and central obesity, and its effect on HF.	**59**
**Table S38** Interaction between sex and central obesity, and its effect on CV death.	**60**
**Table S39** Interaction between sex and central obesity, and its effect on MASLD.	**60**
**Table S40** Interaction between sex and central obesity, and its effect on ESRD.	**61**
**Table S41** Interaction between sex and central obesity, and its effect on all-cause mortality.	**61**
**Table S42** Interaction between sex and the number of metabolic abnormalities, and its effect on ASCVD.	**62**
**Table S43** Interaction between sex and the number of metabolic abnormalities, and its effect on heart failure.	**63**
**Table S44** Sensitivity analysis of the relationship between BMI and metabolic health status (absence or presence of metabolic abnormalities) on cardiovascular outcomes, adjusting for biomarkers of metabolic health profile.	**64**
**Table S45** Sensitivity analysis of the relationship between BMI and metabolic health status on MASLD, adjusting for biomarkers of metabolic health profile.	**68**
**Table S46** Sensitivity analysis of the relationship between BMI and metabolic health status on end-stage renal disease, adjusting for biomarkers of metabolic health profile.	**69**
**Table S47** Sensitivity analysis of the relationship between BMI and metabolic health status on all-cause mortality, adjusting for biomarkers of metabolic health profile.	**70**
**Table S48** Sensitivity analysis of the relationship between BMI and metabolic health status on cardiovascular outcomes among men, adjusting for lifestyle and biological factors.	**71**
**Table S49** Sensitivity analysis of the relationship between BMI and metabolic health status on cardiovascular outcomes among women, adjusting for lifestyle and biological factors.	**75**
**Table S50** Sensitivity analysis of the relationship between BMI and metabolic health status on MASLD among men, adjusting for lifestyle and biological factors.	**79**
**Table S51** Sensitivity analysis of the relationship between BMI and metabolic health status on MASLD among women, adjusting for lifestyle and biological factors.	**80**
**Table S52** Sensitivity analysis of the relationship between BMI and metabolic health status on end-stage renal disease among men, adjusting for lifestyle and biological factors.	**81**

**Table S53** Sensitivity analysis of the relationship between BMI and metabolic health status on end-stage renal disease among women, adjusting for lifestyle and biological factors.	**82**
**Table S54** Sensitivity analysis of the relationship between BMI and metabolic health status on all-cause mortality among men, adjusting for lifestyle and biological factors.	**83**
**Table S55** Sensitivity analysis of the relationship between BMI and metabolic health status on all-cause mortality among women, adjusting for lifestyle and biological factors.	**84**
**Figure S1** Flowchart of participant selection for analysis.	**85**
**Figure S2** Relationship between BMI and the risk of CHD, IS, PAD, MI, and CV death, in men and women by metabolic health status.	**86**
**Figure S3** Relationship between BMI and the risk of strokes (all) and haemorrhagic strokes, in men and women by metabolic health status.	**88**
**Figure S4** Relationship between severity of obesity and metabolic health status on the risk of CHD, IS, PAD, MI, and CV death, in men and women.	**89**
**Figure S5** Relationship between the presence of central obesity within BMI categories and metabolic health status on the risk of CHD, MI, IS, CV death, in men and women.	**91**
**Figure S6** Relationship between the number of metabolic abnormalities present within different categories of BMI and the risk of CHD, ischaemic stroke, PAD, MI, and CV death, in men and women.	**93**
**Figure S7** Sensitivity analysis of the relationship between BMI and metabolic health after adjusting for biomarkers of metabolic health profile in men and women with the risk cardiovascular, hepatic, renal and mortality outcomes.	**95**
**Figure S8** Sensitivity analysis of the relationship between obesity and metabolic health status after adjusting for lifestyle and biological factors in stepwise manner in Men with the risk cardiovascular, hepatic, renal and mortality outcomes.	**98**
**Figure S9** Sensitivity analysis of the relationship between obesity and metabolic health status after adjusting for lifestyle and biological factors in stepwise manner in Women with the risk cardiovascular, hepatic, renal and mortality outcomes.	**101**
**Figure S10** Sensitivity analysis of the relationship between unhealthy metabolic phenotype and BMI after adjusting for lifestyle and biological factors in Men with the risk cardiovascular, hepatic, renal and mortality outcomes.	**104**
**Figure S11** Sensitivity analysis of the relationship between unhealthy metabolic phenotype and BMI after adjusting for lifestyle and biological factors in Men with the risk cardiovascular, hepatic, renal and mortality outcomes.	**107**
**Figure S12** Mediation analysis of the elevated high-sensitivity CRP (≥2 mg/dL) and metabolically healthy obesity phenotype in Men on the risk cardiovascular, hepatic and mortality outcomes.	**110**
**Figure S13** Mediation analysis of the elevated high-sensitivity CRP (≥2 mg/dL) and metabolically healthy obesity phenotype in Women on the risk cardiovascular, hepatic and mortality outcome.	**112**
**Figure S14** Mediation analysis of the elevated high-sensitivity CRP (≥2 mg/dL) and metabolically unhealthy obesity phenotype in Men on the risk cardiovascular, hepatic, renal and mortality outcomes.	**114**
**Figure S15** Mediation analysis of the elevated high-sensitivity CRP (≥2 mg/dL) and metabolically unhealthy obesity phenotype in Women on the risk cardiovascular, hepatic, renal and mortality outcomes.	**117**
**References**	**120**

Supplemental Methods

UK biobank

UK Biobank is a population-based prospective cohort study of over 500,000 participants aged 40-69 years recruited from the general UK population. Between 2006 and 2010, participants underwent physical examination; provided blood, urine and saliva specimens; and completed questionnaires on sociodemographic, lifestyle and behavioural information. Health outcome data were obtained through linkage to electronic health records.

Exposure

BMI was calculated using participants' body weight and standing height, which were measured by trained staff during assessment visits.

Metabolic abnormalities were classified as follows:

- Hypertension[1]
    - clinical diagnosis of hypertension
    - elevated systolic blood pressure (≥140 mmHg), OR
    - elevated diastolic blood pressure (≥90 mmHg), OR
    - taking antihypertensive medications
- Diabetes[2]
    - clinical diagnosis of diabetes
    - elevated fasting plasma glucose (≥126 mg/dL or 7.0 mmol/L), OR
    - elevated random blood glucose (≥200 mg/dL or 11.1 mmol/L), OR
    - elevated glycated haemoglobin (HbA1C; ≥48 mmol/mol or 6.5%), OR
    - taking diabetes medications (insulin or glucose lowering medications)
- Dyslipidaemia[3]
    - clinical diagnosis of dyslipidaemia

- low fasting or non-fasting high-density lipoprotein (HDL-C; ≤1.0 mmol/L for men, ≤1.3 mmol/L for women), OR
- elevated triglycerides (≥2.0 mmol/L), OR
- taking statins or other lipid lowering agents

HDL-C and triglycerides were used for the classification of dyslipidaemia because they are known to associate with insulin resistance and metabolic traits.[4, 5]

Underweight participants (BMI < 18.5 kg/m^2) were excluded from our analysis due to small sample size (n = 878) and its known association with multiple chronic diseases, such as chronic obstructive pulmonary disease (COPD)[6, 7] and malignancy[8, 9], which increases mortality risk from persistent malnourishment and infection, rather than metabolic dysfunction per se.

Arterial Stiffness Index

Arterial stiffness index (ASI) provides an indirect estimate of large artery stiffness measured using a non-invasive technique. Pulse waveform was recorded by clipping a photoplethysmography transducer (PulseTrace PCA2™, CareFusion, USA) to a rested finger or thumb of participants over a period of 10–15 s. The carotid-to-femoral pulse transit time was estimated from the volume of dicrotic pulse waveform in the finger as the time difference between a forward compound of waveform travelling through the arterial tree in the lower body and a reflected compound of waveform back to the finger. ASI was calculated by dividing the participants' standing height by the pulse transit time and was expressed as meters per second (m/s). Participants with extreme outlier ASI values, defined as exceeding ±5 standard deviations from the mean (ASI ≥29.59 m/s), were excluded from this study.

Interaction Analyses

Test for interaction was performed to assess independent and multiplicative effect of obesity, central obesity, and the number of metabolic abnormalities with sex, with adjustments for age in all models, and BMI and metabolic abnormalities in models only when appropriate to the interactions tested. The interaction terms used were “sex*obesity”, “sex*central obesity” and

“sex*number of metabolic abnormalities”. Individuals classified as obesity by BMI (≥30 kg/m^2) were excluded from the interaction analysis for central obesity to avoid collinearity as 87.8% of individuals with obesity had central obesity. We have also tested for the interaction between obesity vs metabolic abnormalities, and central obesity vs metabolic abnormalities, with adjustments for age and sex, but detected no interaction.

Mediation Analyses

An exploratory mediation analysis was performed to determine to what extent inflammation (using hs-CRP, ≥ 2mg/L as a proxy) might mediate any observed association between obesity and outcomes within each metabolic strata. These were conducted using 1000 non-parametric bootstrap simulations to estimate the indirect effect of obesity on each outcome through elevated hs-CRP as the mediator and quantify the proportion mediated by the indirect effect.

Table S1 Ascertainment of cardiovascular, hepatic, and renal outcomes using International Classification of Disease (ICD)-10 and Read v2/v3 codes.

Outcomes	ICD-10 codes	Read v2/v3 codes
**Atherosclerotic cardiovascular disease (ASCVD)**	I21-25, I63, I70-72	G30, G31, G34, G35, G38, G39, G3y, G3z, G63, G64, G6W, G6X, G734
**Coronary Heart Disease**	I21-25	G30, G31, G34, G35, G38, G39, G3y, G3z
**Myocardial Infarction**	I21-23	G30, G31, G35, G38
**Ischaemic Stroke**	I63	G63, G64, G6W, G6X
**Haemorrhagic Stroke**	I60-62	G61
**Strokes (All)**	I60-64	G61, G63, G64, G66, G6W, G6X
**Peripheral Artery Disease**	I70-72	G734
**Heart Failure**	I50	G58
**Metabolic dysfunction-associated liver disease (MASLD)**	K74.6, K75.8, K76.0	C32y5, J6154, J61y1, J61y7, J61y8, J61y9
**Chronic kidney disease stage V**	N18.0, N18.5	1Z1K.00, 1Z1L.00, K050, K055, 1Z1d, 1Z1e,1Z1f
**Renal dialysis or renal transplant**	T82.4, T86.1, Y84.1, Z49, Z94.0, Z99.2	7L1A0, 7L1A1, 7L1A2, 7L1A3, 7L1A4, 7L1A5, 7L1A6, 7B00, 7B000, 7B001, 7B002, 7B003, 7B004, 7B005,7B006, 7B00y, 7B00z, ZV420
**Deaths from chronic kidney disease**	B52.0, D59.3, E10.2, E11.2, E13.2, E14.2, E85.1, I12, I13, I15.0, I15.1, N00-N07, N11, N12, N14, N15, N18, N19, N25-28, N39.1, N39.2, Q60-63	-
**Cardiovascular death**	I20-25, I26, I42-43, I44.7, I46, I49.0, I50, I60-69, I70-72	-
**All-cause mortality**	All death outcomes	-

Table S2 Baseline body mass index for men, stratified by metabolic health status.

Body Mass Index	All Men (n = 70,257)	Metabolically Healthy (n = 17,230)	Metabolically Unhealthy (n = 53,027)
**Normal BMI**, n (%)	18032 (25.7)	7637 (44.3)	10395 (19.6)
**Overweight**, n (%)	34888 (49.6)	7923 (46.0)	26965 (50.9)
**Obesity**, n (%)			
Class I	13526 (19.2)	1433 (8.3)	12093 (22.8)
Class II	2925 (4.2)	197 (1.2)	2728 (5.1)
Class III	886 (1.3)	40 (0.2)	846 (1.6)

Table S3 Baseline body mass index for women, stratified by metabolic health status.

Body Mass Index	All Women (n =86,902)	Metabolically Healthy (n = 32,671)	Metabolically Unhealthy (n = 54,231)
**Normal BMI**, n (%)	34204 (39.4)	18417 (56.4)	15787 (29.1)
**Overweight**, n (%)	31998 (36.8)	10695 (32.7)	21303 (39.3)
**Obesity**, n (%)			
Class I	13797 (15.9)	2732 (8.4)	11065 (20.4)
Class II	4777 (5.5)	645 (2.0)	4132 (7.6)
Class III	2126 (2.4)	182 (0.5)	1944 (3.6)

Table S4 Baseline cardiometabolic profile and medication use for men, excluding those pre-existing cardiovascular disease.

Characteristics	All Men (n = 70,257)	Metabolically Healthy			Metabolically Unhealthy		
		Normal BMI (n = 7,637)	Overweight (n = 7,923)	Obesity (n = 1,670)	Normal BMI (n = 10,395)	Overweight (n = 26,965)	Obesity (n = 15,667)
**Systolic blood pressure** (mmHg), mean (SD)	141 (17)	125 (9)	127 (8)	128 (8)	145 (17)	146 (17)	146 (17)
**HbA1c** (%), mean (SD)	5.5 (0.7)	5.3 (0.4)	5.3 (0.4)	5.4 (0.4)	5.4 (0.7)	5.5 (0.7)	5.7 (10.0)
**HDL cholesterol** (mg/dL), mean (SD)	50 (12)	58 (11)	55 (11)	51 (8)	54 (16)	50 (11)	46 (11)
**LDL cholesterol** (mg/dL), mean (SD)	135 (31)	132 (26)	139 (27)	140 (26)	140 (30)	140 (34)	142 (35)
**Triglycerides**(mg/dL), mean (SD)	168 (97)	106 (35)	115 (35)	124 (36)	159 (89)	195 (106)	213 (115)
**High-sensitivity CRP** (≥ 2mg/L), n (%)	21037 (32.0)	1044 (14.8)	1689 (23.7)	592 (41.3)	2103 (21.3)	8011 (31.3)	7598 (51.6)
**eGFR distribution**, n (%)							
≥ 90 ml/min/1.73 m^2	46256 (65.8)	5659 (74.1)	5197 (65.6)	1010 (60.5)	7272 (70.0)	17110 (63.5)	10008 (63.9)
60 to < 90 ml/min/1.73 m^2	18799 (26.8)	1371 (18.0)	1922 (24.3)	416 (24.9)	2485 (23.9)	8135 (30.2)	4470 (28.5)
30 to < 60 ml/min/1.73 m^2	804 (1.1)	18 (0.2)	30 (0.4)	10 (0.5)	114 (1.1)	361 (1.3)	272 (1.7)
15 to < 30 ml/min/1.73 m^2	32 (0.05)	0	0	0	3 (0.03)	15 (0.06)	14 (0.09)
< 15 ml/min/1.73 m^2	12 (0.02)	0	0	1 (0.06)	1 (0.01)	5 (0.02)	5 (0.03)

†**Missing data:** Systolic blood pressure (314; 0.4%); HbA1c (5071; 7.2%); HDL cholesterol (9541; 13.6%); LDL cholesterol (4464; 6.4%); Triglycerides (4379; 6.3%); High-sensitivity CRP (4489; 6.4%); eGFR distribution (4354; 6.2%).

Table S5 Baseline cardiometabolic profile of for women, excluding those pre-existing cardiovascular disease.

Characteristics	All Women (n = 86,902)	Metabolically Healthy Normal BMI (n = 18,417)	Metabolically Healthy Overweight (n = 10,695)	Metabolically Healthy Obesity (n = 3,559)	Metabolically Unhealthy Normal BMI (n = 15,787)	Metabolically Unhealthy Overweight (n = 21,303)	Metabolically Unhealthy Obesity (n =17,141)
**Systolic blood pressure** (mmHg), mean (SD)	136 (19)	121 (11)	124 (10)	126 (9)	144 (20)	144 (19)	144 (18)
**HbA1c** (%), mean (SD)	5.4 (0.6)	5.3 (0.3)	5.3 (0.3)	5.4 (0.4)	5.4 (0.5)	5.5 (0.6)	5.7 (0.9)
**HDL cholesterol** (mg/dL), mean (SD)	62 (16)	70 (12)	66 (11)	62 (12)	66 (11)	58 (12)	54 (12)
**LDL cholesterol** (mg/dL), mean (SD)	139 (35)	131 (31)	139 (30)	143 (32)	139 (34)	147 (36)	143 (35)
**Triglycerides**(mg/dL), mean (SD)	133 (71)	97 (35)	106 (34)	115 (37)	133 (71)	159 (80)	177 (89)
**High-sensitivity CRP** (≥ 2mg/L), n (%)	29739 (36.8)	2296 (13.6)	2914 (30.0)	1832 (59.5)	3199 (21.4)	8244 (41.1)	11254 (70.3)
**eGFR distribution**, n (%)							
≥ 90 ml/min/1.73 m^2	55530 (63.9)	13073 (71.0)	6818 (63.7)	2090 (58.7)	10562 (66.9)	12965 (60.9)	10022 (58.5)
60 to < 90 ml/min/1.73 m^2	24226 (27.9)	3828 (20.8)	2820 (26.4)	968 (27.2)	4201 (26.6)	6799 (31.9)	5610 (32.7)
30 to < 60 ml/min/1.73 m^2	1032 (1.2)	68 (0.4)	75 (0.7)	27 (0.8)	173 (1.1)	302 (1.4)	387 (2.3)
15 to < 30 ml/min/1.73 m^2	24 (0.03)	2 (0.01)	0	0	6 (0.04)	8 (0.04)	8 (0.05)
< 15 ml/min/1.73 m^2	12 (0.01)	1 (0.01)	0	0	7 (0.04)	2 (0.01)	2 (0.01)

[†]**Missing data:** Systolic blood pressure (455; 0.5%); HbA1c (7263; 8.4%); HDL cholesterol (13834; 15.9%); LDL cholesterol (6183; 7.1%); Triglycerides (6091; 7.0%); High-sensitivity CRP (6166; 7.1%); eGFR distribution (6078; 7.0%).

Table S6 Distribution of medication uses for the control of metabolic health for men, excluding those pre-existing cardiovascular disease.

Medication Use	All Men (n = 70,257)	Metabolically Unhealthy (n = 53,027)		
		**Normal BMI** (n = 10,395)	**Overweight** (n = 26,965)	**Obesity** (n = 15,667)
**Antihypertensive medications**	14873 (21.2)	2070 (19.9)	6848 (25.4)	5955 (38.0)
ACE inhibitor or ARB	9898 (14.1)	1208 (11.6)	4461 (16.5)	4229 (27.0)
Calcium channel blocker	5959 (8.5)	780 (7.5)	2686 (10.0)	2493 (15.9)
Beta blocker	3111 (4.4)	449 (4.3)	1379 (5.1)	1283 (8.2)
Mineralocorticoid receptor antagonist	80 (0.1)	8 (0.1)	24 (0.1)	48 (0.3)
Diuretic	4227 (6.0)	504 (4.8)	1844 (6.8)	1879 (12.0)
**Lipid-lowering medications**	13022 (18.5)	1904 (18.3)	6340 (23.5)	4778 (30.5)
Statin	12774 (18.2)	1864 (17.9)	6230 (23.1)	4680 (29.9)
Ezetimibe	298 (0.4)	36 (0.3)	150 (0.6)	112 (0.7)
Fibrates	291 (0.4)	34 (0.3)	132 (0.5)	125 (0.8)
**Diabetes medications**	3398 (4.8)	404 (3.9)	1317 (4.9)	1677 (10.7)
Metformin	2793 (4.0)	262 (2.5)	1037 (3.8)	1494 (9.5)
Sulphonyureas	1069 (1.5)	117 (1.1)	408 (1.5)	544 (3.5)
GLP-1 agonist	0	0	0	0
Insulin	800 (1.1)	147 (1.4)	321 (1.2)	332 (2.1)
Other diabetes medications	248 (0.4)	12 (0.1)	74 (0.3)	162 (1.0)

Table S7 Distribution of medication uses for the control of metabolic health for women, excluding those pre-existing cardiovascular disease.

Medication Use	All Women (n = 86,902)	Metabolically Unhealthy (n = 54,231)		
		**Normal BMI** (n = 15,787)	**Overweight** (n = 21,303)	**Obesity** (n =17,141)
**Antihypertensive medications**	14951 (17.2)	3160 (20.0)	5543 (26.0)	6248 (36.5)
ACE inhibitor or ARB	8096 (9.3)	1550 (9.8)	2848 (13.4)	3698 (21.6)
Calcium channel blocker	4426 (5.1)	900 (5.7)	1622 (7.6)	1904 (11.1)
Beta blocker	3637 (4.2)	814 (5.2)	1372 (6.4)	1451 (8.5)
Mineralocorticoid receptor antagonist	80 (0.1)	16 (0.1)	27 (0.1)	37 (0.2)
Diuretic	5923 (5.8)	1013 (6.4)	2160 (10.1)	2750 (16.0)
**Lipid-lowering medications**	9636 (11.1)	2037 (12.9)	3796 (17.8)	3803 (22.2)
Statin	9383 (10.8)	1991 (12.6)	3702 (17.4)	3690 (21.5)
Ezetimibe	300 (0.4)	58 (0.4)	113 (0.5)	129 (0.8)
Fibrates	153 (0.2)	25 (0.2)	48 (0.2)	80 (0.5)
**Diabetes medications**	2367 (2.7)	297 (1.9)	701 (3.3)	1369 (8.0)
Metformin	1928 (2.2)	172 (1.1)	554 (2.6)	1202 (7.0)
Sulphonyureas	590 (0.7)	56 (0.4)	165 (0.8)	369 (2.2)
GLP-1 agonist	0	0	0	0
Insulin	609 (0.7)	124 (0.8)	187 (0.9)	298 (1.7)
Other diabetes medications	147 (0.2)	5 (0.03)	28 (0.1)	114 (0.7)

Table S8 Relationship between BMI and metabolic health status (absence or presence of metabolic abnormalities) on cardiovascular outcomes, stratified by sex.

Metabolic Health Status	BMI	By Sex							
		Male				Female			
		Event Rate (per 1,000 person-year)	HR (95% CI)	p-value	p for trend (BMI)	Event Rate (per 1,000 person-year)	HR (95% CI)	p-value	p for trend (BMI)
**Atherosclerotic cardiovascular disease (ASCVD; composite of CHD, IS and PAD)**									
**Metabolically healthy**	**Normal BMI**	6.37	Ref		< 0.001	2.91	Ref		< 0.001
	**Overweight**	6.67	1.09 (0.98 – 1.22)	0.124		3.36	1.10 (0.98 – 1.24)	0.106	
	**Obesity**	8.47	1.46 (1.24 – 1.73)	< 0.001		4.00	1.34 (1.14 – 1.58)	< 0.001	
**Metabolically unhealthy**	**Normal BMI**	12.27	1.51 (1.37 – 1.65)	< 0.001	< 0.001	6.85	1.72 (1.57 – 1.89)	< 0.001	< 0.001
	**Overweight**	13.19	1.68 (1.54 – 1.83)	< 0.001		7.85	1.95 (1.78 – 2.13)	< 0.001	
	**Obesity**	16.64	2.21 (2.03 – 2.41)	< 0.001		9.54	2.51 (2.30 – 2.74)	< 0.001	
**Coronary Heart Disease (CHD; composite of MI and chronic coronary syndrome)**									
**Metabolically healthy**	**Normal BMI**	3.75	Ref		< 0.001	1.34	Ref		< 0.001
	**Overweight**	4.44	1.23 (1.07 – 1.42)	0.003		1.72	1.23 (1.04 – 1.45)	0.018	
	**Obesity**	6.00	1.73 (1.41 – 2.12)	< 0.001		2.40	1.76 (1.42 – 2.19)	< 0.001	
**Metabolically unhealthy**	**Normal BMI**	7.90	1.68 (1.49 – 1.89)	< 0.001	< 0.001	3.83	2.10 (1.84 – 2.40)	< 0.001	< 0.001
	**Overweight**	9.23	2.03 (1.82 – 2.27)	< 0.001		5.02	2.72 (2.40 – 3.08)	< 0.001	
	**Obesity**	12.25	2.80 (2.51 – 3.13)	< 0.001		6.40	3.69 (3.26 – 4.17)	< 0.001	

**Myocardial Infarction (MI)**									
**Metabolically healthy**	**Normal BMI**	1.42	Ref		< 0.001	0.45	Ref		0.029
	**Overweight**	1.80	1.29 (1.04 – 1.61)	0.022		0.60	1.28 (0.96 – 1.70)	0.093	
	**Obesity**	2.19	1.64 (1.17 – 2.28)	0.004		0.69	1.54 (1.04 – 2.28)	0.033	
**Metabolically unhealthy**	**Normal BMI**	2.90	1.70 (1.40 – 2.07)	< 0.001	< 0.001	1.38	2.26 (1.80 – 2.83)	< 0.001	0.003
	**Overweight**	3.58	2.15 (1.80 – 2.57)	< 0.001		1.64	2.64 (2.13 – 3.27)	< 0.001	
	**Obesity**	4.16	2.53 (2.11 – 3.03)	< 0.001		1.65	2.87 (2.31 – 3.58)	< 0.001	
**Strokes (All)**									
**Metabolically healthy**	**Normal BMI**	2.03	Ref		0.094	1.18	Ref		0.156
	**Overweight**	2.12	1.11 (0.92 – 1.35)	0.281		1.39	1.11 (0.92 – 1.34)	0.259	
	**Obesity**	2.23	1.23 (0.89 – 1.69)	0.204		1.39	1.14 (0.87 – 1.50)	0.341	
**Metabolically unhealthy**	**Normal BMI**	4.65	1.59 (1.36 – 1.88)	< 0.001	0.002	3.29	1.74 (1.51 – 2.00)	< 0.001	< 0.001
	**Overweight**	4.19	1.50 (1.29 – 1.75)	< 0.001		3.49	1.78 (1.55 – 2.04)	< 0.001	
	**Obesity**	4.86	1.84 (1.58 – 2.15)	< 0.001		3.73	2.08 (1.81 – 2.38)	< 0.001	
**Ischemic Strokes**									
**Metabolically healthy**	**Normal BMI**	0.91	Ref		0.017	0.37	Ref		0.044
	**Overweight**	1.01	1.19 (0.89 – 1.58)	0.240		0.52	1.33 (0.97 – 1.81)	0.077	
	**Obesity**	1.30	1.61 (1.05 – 2.47)	0.028		0.52	1.36 (0.87 – 2.14)	0.179	
**Metabolically unhealthy**	**Normal BMI**	2.28	1.86 (1.46 – 2.36)	< 0.001	0.073	1.31	2.26 (1.77 – 2.88)	< 0.001	< 0.001

	**Overweight**	2.06	1.76 (1.41 – 2.20)	< 0.001		1.44	2.41 (1.90 – 3.40)	< 0.001	
	**Obesity**	2.33	2.09 (1.66 – 2.63)	< 0.001		1.70	3.10 (2.45 – 3.92)	< 0.001	
**Haemorrhagic Strokes**									
**Metabolically healthy**	**Normal BMI**	0.40	Ref		0.722	0.33	Ref		0.127
	**Overweight**	0.34	0.89 (0.57 – 1.42)	0.647		0.32	0.95 (0.66 – 1.39)	0.805	
	**Obesity**	0.46	1.24 (0.62 – 2.49)	0.545		0.15	0.47 (0.21 – 1.02)	0.057	
**Metabolically unhealthy**	**Normal BMI**	0.72	1.42 (0.97 – 2.07)	0.070	0.898	0.63	1.42 (1.06 – 1.89)	0.019	0.074
	**Overweight**	0.57	1.09 (0.77 – 1.56)	0.613		0.56	1.27 (0.96 – 1.68)	0.098	
	**Obesity**	0.69	1.39 (0.97 – 2.00)	0.073		0.47	1.11 (0.82 – 1.51)	0.484	
**Peripheral Artery Disease**									
**Metabolically healthy**	**Normal BMI**	2.22	Ref		0.976	1.43	Ref		0.724
	**Overweight**	1.93	0.91 (0.75 – 1.10)	0.322		1.44	0.97 (0.81 – 1.16)	0.733	
	**Obesity**	2.09	1.04 (0.76 – 1.44)	0.800		1.37	0.92 (0.70 – 1.21)	0.560	
**Metabolically unhealthy**	**Normal BMI**	3.80	1.27 (1.08 – 1.49)	0.004	0.0008	2.48	1.26 (1.10 – 1.46)	0.001	0.778
	**Overweight**	3.54	1.21 (1.05 – 1.40)	0.012		2.24	1.14 (0.99 – 1.31)	0.059	
	**Obesity**	4.18	1.50 (1.29 – 1.75)	< 0.001		2.34	1.29 (1.12 – 1.48)	< 0.001	
**Heart Failure**									
**Metabolically healthy**	**Normal BMI**	1.28	Ref		0.054	0.55	Ref		< 0.001
	**Overweight**	1.26	1.03 (0.80 – 1.32)	0.788		0.81	1.35 (1.05 – 1.74)	0.019	

	**Obesity**	1.90	1.63 (1.14 – 2.32)	0.007		1.04	1.69 (1.21 – 2.37)	0.002	
**Metabolically unhealthy**	**Normal BMI**	2.82	1.40 (1.14 – 1.71)	< 0.001	< 0.001	1.62	1.72 (1.40 – 2.10)	< 0.001	< 0.001
	**Overweight**	3.24	1.73 (1.44 – 2.08)	< 0.001		2.05	2.10 (1.73 – 2.54)	< 0.001	
	**Obesity**	5.23	2.91 (2.41 – 3.50)	< 0.001		3.37	3.67 (3.04 – 4.43)	< 0.001	
**Cardiovascular Death**									
**Metabolically healthy**	**Normal BMI**	0.92	Ref		0.246	0.38	Ref		0.033
	**Overweight**	0.77	0.88 (0.65 – 1.20)	0.231		0.37	0.92 (0.66 – 1.30)	0.631	
	**Obesity**	1.23	1.49 (0.96 – 2.31)	0.073		0.75	1.85 (1.24 – 2.77)	0.003	
**Metabolically unhealthy**	**Normal BMI**	2.16	1.58 (1.24 – 2.00)	< 0.001	< 0.001	1.16	1.88 (1.47 – 2.41)	< 0.001	< 0.001
	**Overweight**	2.02	1.56 (1.25 – 1.95)	< 0.001		1.12	1.76 (1.38 – 2.24)	< 0.001	
	**Obesity**	3.03	2.41 (1.92 – 3.01)	< 0.001		1.61	2.76 (2.18 – 3.50)	< 0.001	

[†]Adjusted for age, smoking, ethnicity, Townsend deprivation quintiles

Table S9 Baseline characteristics for men, excluding those pre-existing cardiovascular or liver disease.

Characteristics	All Men (n = 70,134)	Metabolically Healthy			Metabolically Unhealthy		
		Normal BMI (n = 7,634)	Overweight (n = 7,918)	Obesity (n = 1,666)	Normal BMI (n = 10,389)	Overweight (n = 26,920)	Obesity (n = 15,607)
**Age**, mean (SD)	56.6 (8.3)	53.8 (8.4)	53.4 (8.2)	52.7 (8.1)	58.0 (8.1)	57.7 (8.1)	57.1 (8.0)
**Ethnicity**†, n (%)							
White	63674 (91.5)	6991 (92.3)	7186 (91.4)	1474 (89.1)	9254 (89.8)	24469 (91.6)	14300 (92.4)
Black	1755 (2.5)	155 (2.0)	275 (3.5)	99 (6.0)	195 (1.9)	610 (2.3)	421 (2.7)
Asian	2914 (4.2)	277 (3.7)	240 (3.1)	37 (2.2)	687 (6.7)	1197 (4.5)	476 (3.1)
Mixed	450 (0.6)	68 (0.9)	59 (0.8)	18 (1.1)	58 (0.6)	145 (0.5)	102 (0.7)
Others	799 (1.1)	82 (1.1)	98 (1.2)	27 (1.6)	105 (1.0)	307 (1.1)	180 (1.1)
**Abdominal obesity**†, n (%)	20667 (29.5)	14 (0.2)	895 (11.3)	1251 (75.1)	42 (0.4)	5417 (20.1)	13048 (83.6)
**Townsend deprivation quintiles†,** n (%)							
Q1 (least deprived)	14098 (20.1)	1495 (19.6)	1666 (21.1)	303 (18.2)	2131 (20.5)	5711 (21.3)	2792 (17.9)
Q2	14020 (20.0)	1481 (19.4)	1572 (19.9)	332 (20.0)	2083 (20.1)	5612 (20.9)	2940 (18.9)
Q3	13943 (19.9)	1426 (18.7)	1645 (20.8)	327 (19.7)	1970 (19.0)	5463 (20.3)	3112 (20.0)
Q4	13978 (20.0)	1611 (21.2)	1580 (20.0)	321 (19.3)	2125 (20.5)	5190 (19.3)	3150 (20.2)
Q5 (most deprived)	13977 (20.0)	1607 (21.1)	1441 (18.2)	380 (22.8)	2067 (19.9)	4902 (18.2)	3580 (23.0)
**Smoking status†,** n (%)							
Never-smoker	35533 (50.7)	4517 (59.2)	4362 (50.1)	839 (50.4)	5701 (54.9)	13176 (48.9)	6938 (44.5)
Ex-smoker	25885 (36.9)	2060 (27.0)	2576 (32.6)	626 (37.6)	3171 (30.5)	10538 (39.1)	6912 (44.3)
Current smoker	8279 (11.8)	1017 (13.3)	934 (11.8)	190 (11.4)	1464 (14.1)	3035 (11.3)	1639 (10.5)
**Physical activity**†							
Summed MET minutes per week for all activity, mean (SD)	2873 (2953)	3112 (3032)	3076 (3022)	2897 (3070)	3000 (2985)	2865 (2927)	2565 (2859)
**Sleep duration**† (hours per day), mean (SD)	7.1 (1.1)	7.1 (1.0)	7.0 (1.0)	7.0 (1.1)	7.2 (1.1)	7.1 (1.1)	7.1 (1.2)
**Alcohol intake frequency**†, n (%)							
Never	4488 (6.4)	530 (6.9)	444 (5.6)	109 (6.5)	759 (7.3)	1582 (5.9)	1064 (6.8)
Special occasion only	5444 (7.8)	535 (7.0)	506 (6.4)	139 (8.3)	802 (7.7)	1984 (7.4)	1478 (9.5)
One to three times a month	6408 (9.1)	701 (9.2)	744 (9.4)	199 (11.9)	848 (8.2)	2279 (8.5)	1637 (10.5)
Once or twice a week	17895 (25.5)	1965 (25.7)	2167 (27.4)	492 (29.5)	2364 (22.8)	6628 (24.6)	4279 (27.4)
Three or four times a week	17800 (25.4)	2021 (26.5)	2192 (27.7)	404 (24.2)	2525 (24.3)	7035 (26.1)	3623 (23.2)

Daily or almost daily	17857 (25.5)	1856 (24.3)	1840 (23.2)	317 (19.0)	3054 (29.4)	7328 (27.2)	3462 (22.2)
**Hypertension**, n (%)	41311 (58.9)	-	-	-	7872 (75.8)	20590 (76.5)	12849 (82.3)
**Diabetes**, n (%)	4347 (6.2)	-	-	-	488 (4.7)	1679 (6.2)	2180 (14.0)
**Dyslipidaemia**, n (%)	34520 (49.2)	-	-	-	5306 (51.1)	17567 (65.3)	11647 (74.6)
**Number of metabolic abnormalities**, n (%)							
0	17218 (24.6)	7634 (100)	7918 (100)	1666 (100)	-	-	-
1	28719 (40.9)	-	-	-	7365 (70.9)	15121 (56.2)	6233 (39.9)
2	21132 (30.1)	-	-	-	2771 (26.7)	10682 (39.7)	7679 (49.2)
3	3065 (4.4)	-	-	-	253 (2.4)	1117 (4.1)	1695 (10.9)
**Prevalence of arterial stiffness** (≥ 10m/s), n (%)	33240 (47.4)	2483 (32.5)	3092 (39.1)	702 (42.1)	4575 (44.0)	13879 (51.6)	8509 (54.5)

[†]**Missing data:** Ethnicity, education qualifications, smoking status, alcohol intake frequency (124; 0.2%); Abdominal obesity (9; 0.01); Townsend deprivation quintiles (118; 0.2%); Sleep duration (470; 0.7%); Physical activity (10639; 15.2%).

Table S10 Baseline characteristics for women, excluding those with pre-existing cardiovascular or liver diseases.

Characteristics	All Women (n = 86,772)	Metabolically Healthy			Metabolically Unhealthy		
		Normal BMI (n = 18,410)	Overweight (n = 10,684)	Obesity (n = 3,555)	Normal BMI (n = 15,777)	Overweight (n = 21,270)	Obesity (n = 17,076)
**Age**, mean (SD)	56.4 (8.1)	53.2 (7.9)	54.0 (7.9)	53.5 (7.7)	58.4 (7.6)	58.7 (7.5)	57.4 (7.8)
**Ethnicity**†, n (%)							
White	78692 (91.2)	17132 (93.5)	9822 (92.4)	3086 (87.4)	14549 (92.7)	19115 (90.3)	14988 (88.4)
Black	2501 (2.9)	247 (1.3)	292 (2.7)	255 (7.2)	178 (1.1)	597 (2.8)	932 (5.5)
Asian	3107 (3.6)	530 (2.9)	269 (2.5)	72 (2.0)	663 (4.2)	969 (4.6)	604 (3.6)
Mixed	768 (0.9)	207 (1.1)	106 (1.0)	40 (1.1)	123 (0.8)	156 (0.7)	136 (0.8)
Others	1230 (1.4)	209 (1.1)	138 (1.3)	77 (2.2)	188 (1.2)	325 (1.5)	293 (1.7)
**Abdominal obesity**†, n (%)	31970 (36.8)	370 (2.0)	2978 (27.9)	3011 (84.7)	757 (4.8)	8919 (41.9)	15935 (93.3)
**Townsend deprivation quintiles†,** n (%)							
Q1 (least deprived)	17362 (20.0)	3894 (21.2)	2205 (20.7)	568 (16.0)	3586 (22.8)	4365 (20.6)	2744 (16.1)
Q2	17342 (20.0)	3763 (20.5)	2175 (20.4)	623 (17.5)	3435 (21.8)	4391 (20.7)	2955 (17.3)
Q3	17305 (20.0)	3778 (20.6)	2115 (19.8)	691 (19.5)	3137 (19.9)	4385 (20.6)	3199 (18.8)
Q4	17312 (20.0)	3795 (20.6)	2180 (20.4)	712 (20.1)	3026 (19.2)	4086 (19.2)	3513 (20.6)
Q5 (most deprived)	17321 (20.0)	3154 (17.1)	1998 (18.7)	954 (26.9)	2563 (16.3)	4013 (18.9)	4639 (27.2)
**Smoking status†,** n (%)							
Never-smoker	52297 (60.3)	11468 (62.3)	6307 (59.0)	2139 (60.2)	9579 (60.7)	12689 (59.7)	10115 (59.2)
Ex-smoker	26835 (30.9)	5342 (29.0)	3445 (32.2)	1118 (31.4)	4674 (29.6)	6775 (31.9)	5481 (32.1)
Current smoker	7141 (8.2)	1525 (8.3)	879 (8.2)	278 (7.8)	1458 (9.2)	1664 (7.8)	1337 (7.8)
**Physical activity**†							
Summed MET minutes per week for all activity, mean (SD)	2627 (2532)	2879 (2619)	2577 (2464)	2266 (2426)	2890 (2640)	2610 (2487)	2189 (2360)
**Sleep duration**† (hours per day), mean (SD)	7.2 (1.1)	7.2 (1.0)	7.2 (1.1)	7.1 (1.2)	7.2 (1.1)	7.2 (1.1)	7.1 (1.3)
**Alcohol intake frequency**†, n (%)							
Never	8790 (10.1)	1288 (7.0)	798 (7.5)	352 (9.9)	1550 (9.8)	2350 (11.0)	2452 (14.4)
Special occasion only	13644 (15.7)	1972 (10.7)	1424 (13.3)	673 (18.9)	2090 (13.2)	3548 (16.7)	3937 (23.1)
One to three times a month	11419 (13.2)	2128 (11.6)	1410 (13.2)	593 (16.7)	1772 (11.2)	2730 (12.8)	2786 (16.3)
Once or twice a week	21573 (24.9)	4872 (26.5)	2925 (27.4)	932 (26.2)	3727 (23.6)	5226 (24.7)	3891 (22.8)
Three or four times a week	17267 (19.9)	4555 (24.7)	2375 (22.2)	592 (16.7)	3351 (21.2)	4058 (19.1)	2336 (13.7)

Daily or almost daily	13826 (15.9)	3550 (19.3)	1723 (16.1)	401 (11.3)	3249 (20.6)	3295 (15.5)	1608 (9.4)
**Hypertension**, n (%)	40926 (47.2)	-	-	-	11788 (74.7)	15805 (74.3)	13333 (78.1)
**Diabetes**, n (%)	3033 (3.5)	-	-	-	344 (2.2)	904 (4.3)	1785 (10.5)
**Dyslipidaemia**, n (%)	31482 (36.3)	-	-	-	7053 (44.7)	12631 (59.4)	11798 (69.1)
**Number of metabolic abnormalities***, n (%)							
0	32649 (37.6)	18410 (100)	10684 (100)	3555 (100)	-	-	-
1	34867 (40.2)	-	-	-	12538 (79.5)	13770 (64.7)	8559 (50.1)
2	17194 (19.8)	-	-	-	3070 (19.4)	6930 (32.6)	7194 (42.1)
3	2062 (2.4)	-	-	-	169 (1.1)	570 (2.7)	1323 (7.8)
**Prevalence of arterial stiffness** (≥ 10m/s), n (%)	26405 (30.4)	3814 (20.7)	2688 (25.2)	1024 (28.8)	4764 (30.2)	7607 (35.8)	6508 (38.1)

†**Missing data:** Ethnicity, education qualifications, smoking status, alcohol intake frequency (144; 0.2%); Abdominal obesity (20; 0.02%); Townsend deprivation quintiles (130; 0.2%); Sleep duration (798; 0.9%); Physical activity (18557; 21.4%).

Table S11 Relationship between BMI and metabolic health status (absence or presence of metabolic abnormalities) on MASLD, stratified by sex.

BMI	Metabolic Health Status	By Sex							
		Male				Female			
		Event Rate (per 1,000 person-year)	HR (95% CI)	p-value	p for trend	Event Rate (per 1,000 person-year)	HR (95% CI)	p-value	p for trend
Metabolically healthy	Normal BMI	0.27	Ref		0.004	0.22	Ref		< 0.001
	Overweight	0.40	1.53 (0.94 – 2.51)	0.089		0.50	2.17 (1.51 – 3.10)	< 0.001	
	Obesity	0.60	2.37 (1.22 – 4.61)	0.011		1.02	4.44 (3.00 – 6.59)	< 0.001	
Metabolically unhealthy	Normal BMI	0.58	1.75 (1.12 – 2.74)	0.014	< 0.001	0.38	1.41 (1.01 – 2.01)	0.049	< 0.001
	Overweight	0.95	3.04 (2.03 – 4.53)	< 0.001		1.02	3.72 (2.77 – 5.01)	< 0.001	
	Obesity	2.16	6.84 (4.60 – 10.18)	< 0.001		2.19	8.17 (6.13 – 10.89)	< 0.001	

†Adjusted for age, smoking, ethnicity, Townsend deprivation quintiles

Table S12 Baseline characteristics for men, excluding those pre-existing cardiovascular or renal disease.

Characteristics	All Men (n = 70,157)	Metabolically Healthy			Metabolically Unhealthy		
		Normal BMI (n = 7,634)	Overweight (n = 7,920)	Obesity (n = 1,668)	Normal BMI (n = 10,374)	Overweight (n = 26,925)	Obesity (n = 15,636)
**Age**, mean (SD)	56.6 (8.3)	53.8 (8.4)	53.4 (8.2)	52.7 (8.1)	58.0 (8.1)	57.7 (8.1)	57.1 (8.0)
**Ethnicity†**, n (%)							
White	63704 (91.5)	6989 (92.3)	7188 (91.5)	1476 (89.1)	9240 (89.8)	24479 (91.6)	14332 (92.4)
Black	1750 (2.5)	156 (2.0)	275 (3.5)	99 (6.0)	195 (1.9)	605 (2.3)	420 (2.7)
Asian	2907 (4.2)	277 (3.7)	240 (3.0)	37 (2.2)	685 (6.7)	1195 (4.5)	473 (3.0)
Mixed	450 (0.6)	68 (0.9)	59 (0.8)	18 (1.1)	58 (0.5)	145 (0.5)	102 (0.7)
Others	805 (1.2)	82 (1.1)	98 (1.2)	27 (1.6)	106 (1.0)	310 (1.2)	182 (1.2)
**Abdominal obesity†**, n (%)	20696 (29.5)	14 (0.2)	895 (11.3)	1253 (75.1)	42 (0.4)	5414 (20.1)	13078 (83.6)
**Townsend deprivation quintiles†,** n (%)							
Q1 (least deprived)	14101 (20.1)	1495 (19.6)	1666 (21.1)	303 (18.2)	2132 (20.5)	5711 (21.2)	2794 (17.9)
Q2	14018 (20.0)	1481 (19.5)	1570 (19.9)	332 (19.9)	2077 (20.0)	5616 (20.9)	2942 (18.9)
Q3	13932 (19.9)	1426 (18.7)	1646 (20.8)	328 (19.7)	1966 (19.0)	5456 (20.3)	3110 (19.9)
Q4	13991 (20.0)	1609 (21.1)	1581 (20.0)	321 (19.3)	2122 (20.5)	5194 (19.3)	3164 (20.3)
Q5 (most deprived)	13997 (20.0)	1609 (21.1)	1443 (18.2)	381 (22.9)	2065 (19.9)	4906 (18.3)	3593 (23.0)
**Smoking status†,** n (%)							
Never-smoker	35531 (50.6)	4519 (59.2)	4365 (55.1)	840 (50.4)	5690 (54.8)	13177 (48.9)	6940 (44.4)
Ex-smoker	25904 (36.9)	2059 (27.0)	2579 (32.6)	627 (37.6)	3167 (30.5)	10542 (39.2)	6930 (44.3)
Current smoker	8284 (11.8)	1016 (13.3)	932 (11.8)	190 (11.4)	1464 (14.1)	3034 (11.3)	1648 (10.5)
**Physical activity†**							
Summed MET minutes per week for all activity, mean (SD)	2871 (2952)	3112 (3032)	3075 (3021)	2897 (3072)	3001 (2986)	2863 (2926)	2560 (2856)
**Sleep duration†** (hours per day), mean (SD)	7.1 (1.1)	7.1 (1.0)	7.0 (1.0)	7.0 (1.1)	7.2 (1.1)	7.1 (1.1)	7.1 (1.2)
**Alcohol intake frequency†**, n (%)							
Never	4505 (6.4)	531 (7.0)	445 (5.6)	109 (6.5)	759 (7.3)	1588 (5.9)	1073 (6.9)
Special occasion only	5449 (7.8)	535 (7.0)	506 (6.4)	139 (8.3)	798 (7.7)	1987 (7.4)	1484 (9.5)
One to three times a month	6408 (9.1)	702 (9.2)	746 (9.4)	199 (11.9)	846 (8.2)	2278 (8.5)	1637 (10.5)
Once or twice a week	17903 (25.5)	1964 (25.7)	2168 (27.4)	493 (29.6)	2363 (22.8)	6625 (24.6)	4290 (27.4)
Three or four times a week	17793 (25.4)	2020 (26.5)	2191 (27.7)	405 (24.3)	2521 (24.3)	7035 (26.1)	3621 (23.1)

Daily or almost daily	17856 (25.5)	1856 (24.3)	1839 (23.2)	317 (19.0)	3050 (29.4)	7327 (27.2)	3467 (22.2)
**Hypertension**, n (%)	41312 (58.9)	-	-	-	7856 (75.7)	20585 (76.5)	12871 (82.3)
**Diabetes**, n (%)	4371 (6.2)	-	-	-	488 (4.7)	1687 (6.3)	2196 (14.0)
**Dyslipidaemia**, n (%)	34529 (49.2)	-	-	-	5293 (51.0)	17569 (65.3)	11667 (74.6)
**Number of metabolic abnormalities**, n (%)							
0	17222 (24.5)	7634 (100)	7920 (100)	1668 (100)	-	-	-
1	28737 (41.0)	-	-	-	7363 (71.0)	15131 (56.2)	6243 (39.9)
2	21119 (30.1)	-	-	-	2759 (26.6)	10672 (39.6)	7688 (49.2)
3	3079 (4.4)	-	-	-	252 (2.4)	1122 (4.2)	1705 (10.9)
**eGFR distribution**, n (%)							
≥ 90 ml/min/1.73 m^2	46228 (65.9)	5657 (74.1)	5195 (65.6)	1010 (60.6)	7262 (70.0)	17100 (63.5)	10004 (64.0)
60 to < 90 ml/min/1.73 m^2	18773 (26.8)	1370 (17.9)	1922 (24.3)	415 (24.9)	2483 (23.9)	8123 (30.2)	4460 (28.5)
30 to < 60 ml/min/1.73 m^2	777 (1.1)	18 (0.2)	30 (0.4)	9 (0.5)	107 (1.0)	351 (1.3)	262 (1.7)
15 to < 30 ml/min/1.73 m^2	31 (0.04)	0	0	0	3 (0.03)	14 (0.05)	14 (0.09)
**Prevalence of arterial stiffness** (≥ 10m/s), n (%)	33260 (47.4)	2481 (32.5)	3094 (39.1)	704 (42.2)	4568 (44.0)	13895 (51.6)	8518 (54.5)

[†]**Missing data:** Ethnicity, education qualifications, smoking status, alcohol intake frequency (124; 0.2%); Abdominal obesity (8; 0.01%); Townsend deprivation quintiles (118; 0.2%); Sleep duration (471; 0.7%); eGFR distribution (4348; 6.2%); Physical activity (10645; 15.2%).

Table S13 Baseline characteristics for women, excluding those with pre-existing cardiovascular or renal diseases.

Characteristics	All Women (n = 86,835)	Metabolically Healthy			Metabolically Unhealthy		
		Normal BMI (n = 18,411)	Overweight (n = 10,689)	Obesity (n = 3,557)	Normal BMI (n = 15,766)	Overweight (n = 21,290)	Obesity (n = 17,122)
**Age**, mean (SD)	56.4 (8.1)	53.2 (7.9)	54.0 (7.9)	53.5 (7.7)	58.4 (7.6)	58.7 (7.5)	57.4 (7.8)
**Ethnicity**†, n (%)							
White	78747 (91.2)	17133 (93.5)	9826 (92.4)	3089 (87.4)	14538 (92.7)	19133 (90.3)	15028 (88.4)
Black	2503 (2.9)	247 (1.4)	293 (2.8)	255 (7.2)	178 (1.1)	597 (2.8)	933 (5.5)
Asian	3112 (3.6)	530 (2.9)	269 (2.5)	72 (2.1)	662 (4.2)	971 (4.6)	608 (3.6)
Mixed	769 (0.9)	207 (1.1)	106 (1.0)	40 (1.1)	123 (0.8)	156 (0.8)	137 (0.8)
Others	1230 (1.4)	209 (1.1)	138 (1.3)	77 (2.2)	188 (1.2)	325 (1.5)	293 (1.7)
**Abdominal obesity**†, n (%)	32042 (36.9)	370 (2.0)	2982 (27.9)	3012 (84.7)	757 (4.8)	8941 (42.0)	15980 (93.3)
**Townsend deprivation quintiles†,** n (%)							
Q1 (least deprived)	17373 (20.0)	3894 (21.2)	2207 (20.7)	570 (16.1)	3586 (22.8)	4370 (20.6)	2746 (16.1)
Q2	17356 (20.0)	3763 (20.5)	2174 (20.4)	624 (17.6)	3434 (21.8)	4397 (20.7)	2964 (17.3)
Q3	17304 (20.0)	3777 (20.5)	2116 (19.8)	690 (19.4)	3135 (19.9)	4386 (20.6)	3200 (18.7)
Q4	17388 (20.1)	3809 (20.7)	2191 (20.5)	715 (20.1)	3030 (19.3)	4104 (19.3)	3539 (20.7)
Q5 (most deprived)	17284 (19.9)	3142 (17.1)	1990 (18.6)	951 (26.8)	2551 (16.2)	4003 (18.8)	4647 (27.2)
**Smoking status†,** n (%)							
Never-smoker	52317 (60.3)	11467 (62.3)	6308 (59.0)	2139 (60.1)	9569 (60.7)	12698 (59.6)	10136 (59.2)
Ex-smoker	26864 (30.9)	5343 (29.0)	3447 (32.2)	1120 (31.5)	4673 (29.6)	6784 (31.9)	5497 (32.1)
Current smoker	7155 (8.2)	1526 (8.3)	881 (8.2)	278 (7.8)	1458 (9.2)	1666 (7.8)	1346 (7.9)
**Physical activity**†							
Summed MET minutes per week for all activity, mean (SD)	2626 (2531)	2878 (2617)	2577 (2464)	2264 (2424)	2890 (2640)	2608 (2486)	2190 (2362)
**Sleep duration**† (hours per day), mean (SD)	7.2 (1.1)	7.2 (1.0)	7.2 (1.1)	7.1 (1.2)	7.2 (1.1)	7.2 (1.1)	7.1 (1.3)
**Alcohol intake frequency**†, n (%)							
Never	8802 (10.1)	1286 (7.0)	800 (7.5)	351 (9.9)	1545 (9.8)	2353 (11.1)	2467 (14.4)
Special occasion only	13658 (15.7)	1973 (10.7)	1426 (13.3)	674 (18.9)	2085 (13.2)	3550 (16.7)	3950 (23.1)
One to three times a month	11429 (13.2)	2129 (11.6)	1411 (13.2)	594 (16.7)	1771 (11.2)	2735 (12.8)	2789 (16.3)
Once or twice a week	21590 (24.9)	4874 (26.5)	2925 (27.4)	933 (26.2)	3727 (23.6)	5228 (24.6)	3903 (22.8)
Three or four times a week	17272 (19.9)	4554 (24.7)	2375 (22.2)	592 (16.6)	3349 (21.2)	4064 (19.1)	2338 (13.7)

Daily or almost daily	13830 (15.9)	3550 (19.3)	1723 (16.1)	401 (11.3)	3250 (20.6)	3297 (15.5)	1609 (9.4)
**Hypertension**, n (%)	40963 (47.2)	-	-	-	11777 (74.7)	15820 (74.3)	13366 (78.1)
**Diabetes**, n (%)	3055 (3.5)	-	-	-	344 (2.2)	913 (4.3)	1798 (10.5)
**Dyslipidaemia**, n (%)	31523 (36.3)	-	-	-	7045 (44.7)	12647 (59.4)	11831 (69.1)
**Number of metabolic abnormalities***, n (%)							
0	32657 (37.6)	18411 (100)	10689 (100)	3557 (100)	-	-	-
1	34892 (40.2)	-	-	-	12534 (79.5)	13776 (64.7)	8582 (50.1)
2	17209 (19.8)	-	-	-	3064 (19.4)	6938 (32.6)	7207 (42.1)
3	2077 (2.4)	-	-	-	168 (1.1)	576 (2.7)	1333 (7.8)
**eGFR distribution at baseline**, n (%)							
≥ 90 ml/min/1.73 m^2	55512 (63.9)	13073 (71.0)	6816 (63.8)	2088 (58.7)	10559 (67.0)	12961 (60.9)	10015 (58.5)
60 to < 90 ml/min/1.73 m^2	24209 (27.9)	3825 (20.8)	2817 (26.4)	968 (27.2)	4199 (26.6)	6795 (31.9)	5605 (32.7)
30 to < 60 ml/min/1.73 m^2	1019 (1.2)	68 (0.4)	75 (0.7)	27 (0.8)	166 (1.1)	300 (1.5)	383 (2.2)
15 to < 30 ml/min/1.73 m^2	22 (0.03)	1 (0.01)	0	0	5 (0.03)	8 (0.04)	8 (0.05)
**Prevalence of arterial stiffness** (≥ 10m/s), n (%)	26439 (30.4)	3812 (20.7)	2689 (25.2)	1024 (28.8)	4761 (30.2)	7625 (35.8)	6528 (38.1)

†**Missing data:** Ethnicity, education qualifications, smoking status, alcohol intake frequency (144; 0.2%); Abdominal obesity (20; 0.02%); Townsend deprivation quintiles (130; 0.2%); Sleep duration (805; 0.9%); eGFR distribution (6073; 7.0%); Physical activity (18572; 21.4%).

Table S14 Relationship between BMI and metabolic health status (absence or presence of metabolic abnormalities) on end-stage renal disease, stratified by sex.

Metabolic Health Status	BMI	By Sex							
		Male				Female			
		Event Rate (per 1,000 person-year)	HR (95% CI)	p-value	p for trend	Event Rate (per 1,000 person-year)	HR (95% CI)	p-value	p for trend
Metabolically healthy	Normal BMI	0.11	Ref		0.751	0.04	Ref		0.077
	Overweight	0.08	0.72 (0.29 – 1.79)	0.476		0.06	1.44 (0.55 – 3.73)	0.454	
	Obesity	0.09	0.82 (0.18 – 3.71)	0.799		0.11	2.58 (0.86 – 7.72)	0.089	
Metabolically unhealthy	Normal BMI	0.30	1.96 (1.01 – 3.82)	0.049	< 0.001	0.17	3.22 (1.54 – 6.72)	0.002	< 0.001
	Overweight	0.44	3.00 (1.62 – 5.55)	< 0.001		0.24	4.26 (2.11 – 8.60)	< 0.001	
	Obesity	0.78	5.42 (2.94 – 10.02)	< 0.001		0.46	7.96 (4.00 – 15.85)	< 0.001	

†Adjusted for age, smoking, ethnicity, Townsend deprivation quintiles

Table S15 Relationship between BMI and metabolic health status (absence or presence of metabolic abnormalities) on all-cause mortality, stratified by sex.

Metabolic Health Status	BMI	By Sex							
		Men				Women			
		Event Rate (per 1,000 person-year)	HR (95% CI)	p-value	p for trend (BMI)	Event Rate (per 1,000 person-year)	HR (95% CI)	p-value	p for trend (BMI)
Metabolically healthy	Normal BMI	4.07	Ref		0.096	2.39	Ref		0.009
	Overweight	3.46	0.89 (0.77 – 1.03)	0.130		2.75	1.08 (0.95 – 1.23)	0.252	
	Obesity	5.00	1.36 (1.10 – 1.69)	0.005		3.18	1.27 (1.05 – 1.52)	0.012	
Metabolically unhealthy	Normal BMI	7.65	1.32 (1.18 – 1.49)	< 0.001	< 0.001	4.87	1.33 (1.20 – 1.48)	< 0.001	< 0.001
	Overweight	6.57	1.18 (1.06 – 1.31)	0.003		4.74	1.28 (1.16 – 1.42)	< 0.001	
	Obesity	8.79	1.62 (1.45 – 1.81)	< 0.001		5.68	1.67 (1.51 – 1.85)	< 0.001	

†Adjusted for age, smoking, ethnicity, Townsend deprivation quintiles

Table S16 Relationship between obesity severity and metabolic health status (absence or presence of metabolic abnormalities) on cardiovascular outcomes, stratified by sex.

Metabolic Health Status	BMI	By Sex							
		Male				Female			
		Event Rate (per 1,000 person-year)	HR (95% CI)	p-value	p for trend	Event Rate (per 1,000 person-year)	HR (95% CI)	p-value	p for trend
**Atherosclerotic cardiovascular disease**									
Metabolically healthy	**Normal BMI**	6.37	Ref			2.91	Ref		
	**Obesity (Class I)**	8.35	1.45 (1.21 – 1.74)	< 0.001	0.928	3.86	1.28 (1.06 – 1.54)	0.009	0.276
	**Obesity (Class II)**	10.41	1.74 (1.15 – 2.65)	0.009		4.52	1.53 (1.10 – 2.13)	0.012	
	**Obesity (Class III)**	3.84	0.65 (0.16 – 2.61)	0.544		4.32	1.63 (0.87 – 3.05)	0.124	
Metabolically unhealthy	**Obesity (Class I)**	15.91	2.09 (1.91 – 2.29)	< 0.001	< 0.001	9.07	2.32 (2.11 – 2.55)	< 0.001	< 0.001
	**Obesity (Class II)**	18.50	2.52 (2.25 – 2.82)	< 0.001		9.74	2.61 (2.32 – 2.93)	< 0.001	
	**Obesity (Class III)**	21.21	3.01 (2.57 – 3.53)	< 0.001		11.85	3.51 (3.05 – 4.04)	< 0.001	
**Coronary Heart Disease**									
Metabolically healthy	**Normal BMI**	3.75	Ref			1.34	Ref		
	**Obesity (Class I)**	5.93	1.73 (1.40 – 2.16)	< 0.001	0.961	2.19	1.59 (1.24 – 2.04)	< 0.001	0.046
	**Obesity (Class II)**	6.89	1.84 (1.10 – 3.08)	0.021		3.03	2.24 (1.49 – 3.36)	< 0.001	
	**Obesity (Class III)**	3.90	1.14 (0.28 – 4.58)	0.852		3.43	2.84 (1.41 – 5.73)	0.004	
	**Obesity (Class I)**	11.69	2.65	< 0.001	< 0.001	5.84	3.27	< 0.001	< 0.001

			(2.36 – 2.97)				(2.87 – 3.73)		
**Metabolically unhealthy**	**Obesity (Class II)**	13.55	3.15 (2.74 – 3.63)	< 0.001		6.86	4.02 (3.45 – 4.68)	< 0.001	
	**Obesity (Class III)**	16.27	3.94 (3.27 – 4.74)	< 0.001		8.70	5.57 (4.67 – 6.65)	< 0.001	
**Myocardial Infarction**									
**Metabolically healthy**	**Normal BMI**	1.42	Ref			0.45	Ref		
	**Obesity (Class I)**	2.28	1.70 (1.20 – 2.40)	0.003		0.68	1.49 (0.95 – 2.32)	0.079	
	**Obesity (Class II)**	1.97	1.52 (0.62 – 3.72)	0.356	0.373	0.84	1.89 (0.88 – 4.05)	0.104	0.763
	**Obesity (Class III)**	0	-	-		0.42	1.07 (0.15 – 7.64)	0.949	
**Metabolically unhealthy**	**Obesity (Class I)**	4.07	2.46 (2.05 – 2.96)	0.022		1.53	2.59 (2.05 – 3.27)	< 0.001	
	**Obesity (Class II)**	4.22	2.59 (2.05 – 3.27)	< 0.001	0.083	1.71	3.07 (2.31 – 4.07)	< 0.001	0.0021
	**Obesity (Class III)**	5.32	3.26 (2.39 – 4.46)	< 0.001		2.19	4.29 (3.09 – 5.97)	< 0.001	
**Strokes (All)**									
**Metabolically healthy**	**Normal BMI**	2.03	Ref			1.18	Ref		
	**Obesity (Class I)**	2.33	1.27 (0.91 – 1.78)	0.154		1.47	1.19 (0.88 – 1.60)	0.252	0.774
	**Obesity (Class II)**	1.18	0.69 (0.22 – 2.17)	0.529	0.637	0.96	0.80 (0.40 – 1.63)	0.544	
	**Obesity (Class III)**	3.86	2.16 (0.54 – 8.71)	0.278		1.70	1.69 (0.63 – 4.54)	0.298	
**Metabolically unhealthy**	**Obesity (Class I)**	4.60	1.70 (1.45 – 2.00)	< 0.001		3.82	2.03 (1.75 – 2.35)	< 0.001	0.225
	**Obesity (Class II)**	5.59	2.26 (1.85 – 2.76)	< 0.001	< 0.001	3.56	2.07 (1.72 – 2.50)	< 0.001	
	**Obesity (Class III)**	6.35	2.64	< 0.001		3.60	2.41	< 0.001	

			(2.00 – 3.49)				(1.89 – 3.06)		
**Ischemic Strokes**									
**Metabolically healthy**	**Normal BMI**	0.91	Ref			0.37	Ref		
	**Obesity (Class I)**	1.34	1.67 (1.07 – 2.61)	0.024	0.391	0.62	1.60 (1.01 – 2.56)	0.048	0.108
	**Obesity (Class II)**	1.18	1.52 (0.48 – 4.81)	0.475		0.24	0.64 (0.16 – 2.60)	0.533	
	**Obesity (Class III)**	0	-	-		0	-	-	
**Metabolically unhealthy**	**Obesity (Class I)**	1.60	1.93 (1.53 – 2.45)	< 0.001	0.003	1.72	3.01 (2.35 – 3.85)	< 0.001	0.336
	**Obesity (Class II)**	2.61	2.48 (1.85 – 3.33)	< 0.001		1.67	3.14 (2.33 – 4.23)	< 0.001	
	**Obesity (Class III)**	3.22	3.14 (2.12 – 4.65)	< 0.001		1.67	3.65 (2.52 – 5.29)	< 0.001	
**Peripheral Artery Disease**									
**Metabolically healthy**	**Normal BMI**	2.22	Ref			1.43	Ref		
	**Obesity (Class I)**	2.01	1.00 (0.70 – 1.41)	0.980	0.970	1.33	0.88 (0.65 – 1.20)	0.426	0.656
	**Obesity (Class II)**	3.19	1.63 (0.81 – 3.31)	0.173		1.69	1.17 (0.68 – 1.99)	0.573	
	**Obesity (Class III)**	0	-	-		0.85	0.66 (0.16 – 2.66)	0.561	
**Metabolically unhealthy**	**Obesity (Class I)**	4.02	1.42 (1.22 – 1.66)	< 0.001	0.002	2.42	1.25 (1.07 – 1.45)	0.005	0.136
	**Obesity (Class II)**	4.60	1.71 (1.39 – 2.10)	< 0.001		2.31	1.25 (1.01 – 1.54)	0.037	
	**Obesity (Class III)**	5.22	2.05 (1.53 – 2.75)	< 0.001		2.79	1.65 (1.27 – 2.15)	< 0.001	
**Heart Failure**									
**Metabolically healthy**	**Normal BMI**	1.28	Ref			0.55	Ref		
	**Obesity (Class I)**	1.57	1.36 (0.91 – 2.03)	0.138	0.0044	0.90	1.45 (0.98 – 2.16)	0.061	0.0065

	**Obesity (Class II)**	3.60	3.12 (1.53 – 6.38)	0.002		1.20	1.95 (1.01 – 3.83)	0.049	
	**Obesity (Class III)**	5.82	5.26 (1.67 – 16.55)	0.005		2.58	5.96 (2.63 – 13.52)	< 0.001	
**Metabolically unhealthy**	**Obesity (Class I)**	4.44	2.46 (2.03 – 2.98)	< 0.001	< 0.001	2.75	2.93 (2.40 – 3.58)	< 0.001	< 0.001
	**Obesity (Class II)**	7.01	4.10 (3.30 – 5.10)	< 0.001		3.67	4.22 (3.38 – 5.28)	< 0.001	
	**Obesity (Class III)**	11.12	5.93 (4.59 – 7.67)	< 0.001		6.26	7.50 (5.90 – 9.52)	< 0.001	
**Cardiovascular Death**									
**Metabolically healthy**	**Normal BMI**	0.92	Ref			0.38	Ref		
	**Obesity (Class I)**	1.27	1.54 (0.97 – 2.43)	0.066	0.843	0.57	1.43 (0.88 – 2.33)	0.145	0.005
	**Obesity (Class II)**	0.80	1.01 (0.25 – 4.10)	0.989		1.09	2.91 (1.46 – 5.77)	< 0.001	
	**Obesity (Class III)**	1.92	2.13 (0.30 – 15.30)	0.452		2.15	5.70 (2.09 – 15.55)	< 0.001	
**Metabolically unhealthy**	**Obesity (Class I)**	2.60	2.03 (1.61 – 2.56)	< 0.001	< 0.001	1.33	2.18 (1.68 – 2.81)	< 0.001	< 0.001
	**Obesity (Class II)**	3.81	3.18 (2.43 – 4.18)	< 0.001		1.61	2.86 (2.11 – 3.87)	< 0.001	
	**Obesity (Class III)**	6.79	5.94 (4.33 – 8.15)	< 0.001		3.21	6.73 (4.95 – 9.16)	< 0.001	

†Adjusted for age, smoking, ethnicity, Townsend deprivation quintiles

Table S17 Relationship between obesity severity and metabolic health status (absence or presence of metabolic abnormalities) on MASLD, stratified by sex.

Metabolic Health Status	BMI	By Sex							
		Male				Female			
		Event Rate (per 1,000 person-year)	HR (95% CI)	p-value	p for trend	Event Rate (per 1,000 person-year)	HR (95% CI)	p-value	p for trend
Metabolically healthy	**Normal BMI**	0.27	Ref		0.048	0.22	Ref		0.049
	**Obesity (Class I)**	0.48	1.91 (0.89 – 4.07)	0.096		0.82	3.57 (2.27 – 5.62)	< 0.001	
	**Obesity (Class II)**	1.58	6.43 (2.24 – 18.42)	< 0.001		1.93	8.37 (4.78 – 14.66)	< 0.001	
	**Obesity (Class III)**	0	-	-		0.86	4.05 (0.98 – 16.61)	0.052	
Metabolically unhealthy	**Obesity (Class I)**	1.84	5.80 (3.88 – 8.68)	< 0.001	< 0.001	1.68	6.14 (4.54 – 8.29)	< 0.001	< 0.001
	**Obesity (Class II)**	3.26	10.40 (6.78 – 15.96)	< 0.001		2.55	9.58 (6.95 – 13.20)	< 0.001	
	**Obesity (Class III)**	3.25	10.58 (6.36 – 17.59)	< 0.001		4.41	17.32 (12.43 – 24.14)	< 0.001	

[†]Adjusted for age, smoking, ethnicity, Townsend deprivation quintiles

Table S18 Relationship between obesity severity and metabolic health status on end-stage renal disease, stratified by sex.

Metabolic Health Status	BMI	By Sex							
		Male				Female			
		Event Rate (per 1,000 person-year)	HR (95% CI)	p-value	p for trend	Event Rate (per 1,000 person-year)	HR (95% CI)	p-value	p for trend
Metabolically healthy	**Normal BMI**	0.11	Ref	-	-	0.04	Ref	-	-
	**Obesity (Class I)**	0.11	0.95 (0.21 – 4.31)	0.952	1.00	0.08	2.03 (0.55 – 7.52)	0.288	0.213
	**Obesity (Class II)**	0	-	-		0.12	2.82 (0.36 – 22.32)	0.325	
	**Obesity (Class III)**	0	-	-		0.42	11.02 (1.39 – 87.13)	0.023	
Metabolically unhealthy	**Obesity (Class I)**	0.67	4.61 (2.47 – 8.61)	< 0.001	< 0.001	0.35	5.99 (2.93 – 12.25)	< 0.001	< 0.001
	**Obesity (Class II)**	1.13	8.18 (4.18 – 16.00)	< 0.001		0.52	8.99 (4.21 – 19.24)	< 0.001	
	**Obesity (Class III)**	1.18	8.71 (3.89 – 19.47)	< 0.001		0.99	18.02 (8.29 – 39.16)	< 0.001	

[†]Adjusted for age, smoking, ethnicity, Townsend deprivation quintiles

Table S19 Relationship between obesity severity and metabolic health status on all-cause mortality, stratified by sex.

Metabolic Health Status	BMI	By Sex							
		Men				Women			
		Event Rate (per 1,000 person-year)	HR (95% CI)	p-value	p for trend	Event Rate (per 1,000 person-year)	HR (95% CI)	p-value	p for trend
Metabolically healthy	**Normal BMI**	4.07	Ref	-	-	2.39	Ref	-	-
	**Obesity (Class I)**	4.95	1.34 (1.07 – 1.69)	0.012	0.871	3.05	1.21 (0.98 – 1.49)	0.069	0.099
	**Obesity (Class II)**	5.62	1.59 (0.93 – 2.71)	0.087		3.15	1.26 (0.85 – 1.89)	0.251	
	**Obesity (Class III)**	3.84	0.97 (0.24 – 3.90)	0.969		5.16	2.37 (1.30 – 4.31)	0.005	
Metabolically unhealthy	**Obesity (Class I)**	8.10	1.47 (1.31 – 1.65)	< 0.001	< 0.001	5.16	1.46 (1.30 – 1.64)	< 0.001	< 0.001
	**Obesity (Class II)**	10.01	1.93 (1.67 – 2.24)	< 0.001		6.16	1.87 (1.62 – 2.14)	< 0.001	
	**Obesity (Class III)**	15.02	3.02 (2.50 – 3.65)	< 0.001		7.59	2.66 (2.25 – 3.14)	< 0.001	

†Adjusted for age, smoking, ethnicity, Townsend deprivation quintiles

Table S20 Relationship between BMI, central obesity and metabolic health status on cardiovascular outcomes, stratified by sex.

BMI	Metabolic Health Status and Central Obesity†	By Sex					
		Male			Female		
		Event Rate (per 1,000 person-year)	HR (95% CI)	p-value	Event Rate (per 1,000 person-year)	HR (95% CI)	p-value
**Atherosclerotic Cardiovascular Disease**							
**Normal BMI**	**MH – CO**	6.36	Ref		2.89	Ref	
	**MH + CO**	12.36	1.54 (0.38 – 6.18)	0.540	4.01	1.24 (0.78 – 1.95)	0.358
	**MU – CO**	12.21	1.50 (1.37 – 1.65)	< 0.001	6.76	1.72 (1.56 – 1.89)	< 0.001
	**MU + CO**	26.40	2.31 (1.33 – 4.00)	0.003	8.68	2.01 (1.59 – 2.53)	< 0.001
**Overweight**	**MH – CO**	6.49	1.09 (0.97 – 1.22)	0.149	3.26	1.10 (0.97 – 1.26)	0.147
	**MH + CO**	8.17	1.12 (0.90 – 1.39)	0.330	3.63	1.12 (0.93 – 1.35)	0.221
	**MU – CO**	12.54	1.65 (1.51 – 1.80)	< 0.001	6.89	1.78 (1.61 – 1.97)	< 0.001
	**MU + CO**	15.81	1.81 (1.63 – 2.00)	< 0.001	9.19	2.19 (1.98 – 2.42)	< 0.001
**Obesity**	**MH – CO**	6.77	1.37 (0.97 – 1.93)	0.070	2.73	1.00 (0.64 – 1.58)	0.990
	**MH + CO**	9.06	1.49 (1.24 – 1.80)	< 0.001	4.24	1.40 (1.18 – 1.67)	< 0.001
	**MU – CO**	13.40	2.01 (1.77 – 2.27)	< 0.001	7.30	2.06 (1.67 – 2.53)	< 0.001
	**MU + CO**	17.29	2.25 (2.06 – 2.46)	< 0.001	9.70	2.56 (2.34 – 2.80)	< 0.001

**Coronary Heart Disease**							
**Normal BMI**	**MH – CO**	3.75	Ref		1.30	Ref	
	**MH + CO**	5.84	1.25 (0.18 – 8.91)	0.823	3.16	2.17 (1.29 – 3.64)	0.004
	**MU – CO**	7.85	1.67 (1.48 – 1.88)	< 0.001	3.82	2.17 (1.89 – 2.48)	< 0.001
	**MU + CO**	19.48	3.04 (1.62 – 5.70)	< 0.001	3.99	2.08 (1.49 – 2.90)	< 0.001
**Overweight**	**MH – CO**	4.35	1.23 (1.07 – 1.42)	0.004	1.59	1.19 (0.98 – 1.44)	0.081
	**MH + CO**	5.17	1.23 (0.93 – 1.62)	0.145	2.07	1.43 (1.12 – 1.83)	0.005
	**MU – CO**	8.83	2.00 (1.79 – 2.24)	< 0.001	4.38	2.53 (2.21 – 2.90)	< 0.001
	**MU + CO**	10.8	2.15 (1.89 – 2.44)	< 0.001	5.91	3.13 (2.72 – 3.59)	< 0.001
**Obesity**	**MH – CO**	5.38	1.80 (1.23 – 2.65)	0.003	1.28	1.05 (0.54 – 2.04)	0.887
	**MH + CO**	6.21	1.72 (1.37 – 2.15)	< 0.001	2.61	1.93 (1.54 – 2.42)	< 0.001
	**MU – CO**	10.30	2.62 (2.26 – 3.05)	< 0.001	5.33	3.35 (2.60 – 4.31)	< 0.001
	**MU + CO**	12.64	2.84 (2.53 – 3.18)	< 0.001	6.47	3.81 (3.36 – 4.33)	< 0.001
**Myocardial Infarction**							
**Normal BMI**	**MH – CO**	1.43	Ref		0.43	Ref	
	**MH + CO**	0	-	-	1.25	2.63 (1.15 – 6.00)	0.021
	**MU – CO**	2.88	1.65 (1.36 – 2.00)	< 0.001	1.37	2.34 (1.85 – 2.95)	< 0.001
	**MU + CO**	7.64	3.31 (1.23 – 8.97)	0.018	1.62	2.51 (1.48 – 4.26)	0.0007

**Overweight**	**MH – CO**	1.75	1.30 (1.04 – 1.64)	0.023	0.57	1.28 (0.92 – 1.77)	0.144
	**MH + CO**	2.16	1.37 (0.90 – 2.10)	0.146	0.70	1.44 (0.94 – 2.21)	0.092
	**MU – CO**	3.46	2.13 (1.78 – 2.55)	< 0.001	1.37	2.40 (1.89 – 3.05)	< 0.001
	**MU + CO**	4.02	2.21 (1.80 – 2.72)	< 0.001	2.02	3.17 (2.50 – 4.03)	< 0.001
**Obesity**	**MH – CO**	2.27	1.98 (1.10 – 3.57)	0.024	0.57	1.40 (0.52 – 3.81)	0.505
	**MH + CO**	2.17	1.60 (1.10 – 2.32)	0.013	0.72	1.63 (1.07 – 2.47)	0.023
	**MU – CO**	4.04	2.71 (2.13 – 3.45)	< 0.001	1.69	3.13 (2.00 – 4.89)	< 0.001
	**MU + CO**	4.19	2.55 (2.11 – 3.05)	< 0.001	1.64	2.97 (2.37 – 3.72)	< 0.001
**Strokes (All)**							
**Normal BMI**	**MH – CO**	2.03	Ref		1.17	Ref	
	**MH + CO**	5.76	2.15 (0.30 – 15.35)	0.445	1.88	1.37 (0.71 – 2.67)	0.350
	**MU – CO**	4.62	1.59 (1.36 – 1.88)	< 0.001	3.24	1.74 (1.50 – 2.01)	< 0.001
	**MU + CO**	11.39	2.79 (1.24 – 6.30)	0.013	4.42	2.07 (1.49 – 2.87)	< 0.001
**Overweight**	**MH – CO**	2.03	1.11 (0.91 – 1.36)	0.303	1.26	1.06 (0.86 – 1.31)	0.571
	**MH + CO**	2.85	1.14 (0.78 – 1.64)	0.502	1.71	1.26 (0.96 – 1.65)	0.097
	**MU – CO**	3.91	1.46 (1.25 – 1.70)	< 0.001	2.92	1.56 (1.34 – 1.82)	< 0.001
	**MU + CO**	5.33	1.67 (1.40 – 1.99)	< 0.001	4.29	2.10 (1.81 – 2.45)	< 0.001

**Obesity**	**MH – CO**	2.08	1.49 (0.81 – 2.73)	0.201	0.99	0.94 (0.44 – 1.99)	0.874
	**MH + CO**	2.29	1.17 (0.82 – 1.68)	0.378	1.46	1.19 (0.89 – 1.58)	0.241
	**MU – CO**	3.45	1.54 (1.22 – 1.94)	< 0.001	2.51	1.52 (1.08 – 2.15)	0.017
	**MU + CO**	5.14	1.90 (1.62 – 2.22)	< 0.001	3.82	2.14 (1.86 – 2.47)	< 0.001
**Ischaemic Strokes**							
**Normal BMI**	**MH – CO**	0.90	Ref		0.37	Ref	
	**MH + CO**	5.66	4.90 (0.68 – 35.19)	0.114	0	-	-
	**MU – CO**	2.25	1.86 (1.46 – 2.37)	< 0.001	1.28	2.17 (1.69 – 2.77)	< 0.001
	**MU + CO**	7.54	4.44 (1.62 – 12.13)	0.004	2.03	2.96 (1.80 – 4.87)	< 0.001
**Overweight**	**MH – CO**	0.95	1.17 (0.87 – 1.58)	0.309	0.49	1.27 (0.89 – 1.80)	0.182
	**MH + CO**	1.46	1.37 (0.82 – 2.31)	0.234	0.52	1.35 (0.85 – 2.14)	0.200
	**MU – CO**	1.95	1.74 (1.39 – 2.19)	< 0.001	1.13	1.92 (1.48 – 2.49)	< 0.001
	**MU + CO**	2.49	1.90 (1.46 – 2.46)	< 0.001	1.88	2.91 (2.26 – 3.74)	< 0.001
**Obesity**	**MH – CO**	1.13	1.70 (0.74 – 3.89)	0.209	0.57	1.65 (0.60 – 4.49)	0.329
	**MH + CO**	1.35	1.61 (1.01 – 2.57)	0.046	0.51	1.29 (0.79 – 2.09)	0.313
	**MU – CO**	1.59	1.66 (1.18 – 2.34)	0.004	0.74	1.41 (0.75 – 2.65)	0.280
	**MU + CO**	2.47	2.19 (1.73 – 2.76)	< 0.001	1.77	3.15 (2.48 – 3.99)	< 0.001

**Heart Failure**							
**Normal BMI**	**MH – CO**	1.28	Ref		0.55	Ref	
	**MH + CO**	0	-	-	0.84	1.25 (0.46 – 3.38)	0.661
	**MU – CO**	2.81	1.39 (1.14 – 1.71)	0.001	1.63	1.75 (1.42 – 2.15)	< 0.001
	**MU + CO**	5.59	1.87 (0.59 – 5.88)	0.285	1.52	1.46 (0.85 – 2.50)	0.167
**Overweight**	**MH – CO**	1.16	1.00 (0.77 – 1.30)	0.997	0.64	1.13 (0.83 – 1.52)	0.435
	**MH + CO**	1.98	1.21 (0.77 – 1.88)	0.411	1.26	1.88 (1.35 – 2.62)	< 0.001
	**MU – CO**	2.94	1.65 (1.36 – 1.99)	< 0.001	1.69	1.82 (1.47 – 2.25)	< 0.001
	**MU + CO**	4.48	2.01 (1.63 – 2.47)	< 0.001	2.55	2.48 (2.01 – 3.07)	< 0.001
**Obesity**	**MH – CO**	0.75	0.86 (0.32 – 2.33)	0.767	0.42	0.86 (0.28 – 2.72)	0.804
	**MH + CO**	2.29	1.81 (1.25 – 2.62)	0.002	1.15	1.83 (1.30 – 2.59)	< 0.001
	**MU – CO**	2.90	1.93 (1.48 – 2.52)	< 0.001	1.83	2.23 (1.47 – 3.38)	< 0.001
	**MU + CO**	5.69	3.07 (2.54 – 3.70)	< 0.001	3.48	3.80 (3.14 – 4.60)	< 0.001
**Cardiovascular Death**							
**Normal BMI**	**MH – CO**	0.93	Ref		0.37	Ref	
	**MH + CO**	0	-	-	0.63	1.43 (0.45 – 4.52)	0.544
	**MU – CO**	2.14	1.56 (1.23 – 1.99)	< 0.001	1.17	1.93 (1.50 – 2.48)	< 0.001
	**MU + CO**	7.84	3.58 (1.32 – 9.76)	0.013	1.03	1.48 (0.77 – 2.86)	0.242

**Overweight**	**MH – CO**	0.76	0.92<br>(0.67 – 1.27)	0.621	0.32	0.85<br>(0.56 – 1.27)	0.422
	**MH + CO**	0.79	0.65<br>(0.33 – 1.30)	0.222	0.50	1.11<br>(0.68 – 1.83)	0.678
	**MU – CO**	1.78	1.44<br>(1.14 – 1.80)	0.002	0.87	1.44<br>(1.09 – 1.89)	0.010
	**MU + CO**	2.98	1.96<br>(1.52 – 2.51)	< 0.001	1.47	2.21<br>(1.70 – 2.88)	< 0.001
**Obesity**	**MH – CO**	1.15	1.83<br>(0.80 – 4.20)	0.151	0.29	0.84<br>(0.21 – 3.41)	0.806
	**MH + CO**	1.26	1.41<br>(0.87 – 2.29)	0.165	0.83	2.04<br>(1.35 – 3.08)	< 0.001
	**MU – CO**	1.96	1.85<br>(1.34 – 2.56)	< 0.001	0.81	1.57<br>(0.86 – 2.87)	0.145
	**MU + CO**	3.24	2.50<br>(2.00 – 3.14)	< 0.001	1.66	2.88<br>(2.26 – 3.67)	< 0.001

†Adjusted for age, smoking, ethnicity, Townsend deprivation quintiles

† MH refers to metabolically healthy; MU refers to metabolically unhealthy; CO refers to central obesity.

Table S21 Relationship between BMI, central obesity and metabolic health status on MASLD, stratified by sex.

BMI	Metabolic Health Status and Central Obesity†	By Sex					
		Male			Female		
		Event Rate (per 1,000 person-year)	HR (95% CI)	p-value	Event Rate (per 1,000 person-year)	HR (95% CI)	p-value
Normal BMI	**MH – CO**	0.27	Ref		0.21	Ref	
	**MH + CO**	0	-	-	0.63	2.74 (0.85 – 8.79)	0.090
	**MU – CO**	0.58	1.77 (1.13 – 2.77)	0.012	0.34	1.32 (0.91 – 1.91)	0.143
	**MU + CO**	0	-	-	1.21	4.50 (2.39 – 8.47)	< 0.001
Overweight	**MH – CO**	0.36	1.41 (0.84 – 2.37)	0.192	0.35	1.61 (1.05 – 2.49)	0.030
	**MH + CO**	0.68	2.29 (1.04 – 5.06)	0.040	0.90	3.80 (2.45 – 5.87)	< 0.001
	**MU – CO**	0.82	2.71 (1.80 – 4.07)	< 0.001	0.67	2.61 (1.86 – 3.66)	< 0.001
	**MU + CO**	1.48	4.26 (2.77 – 6.57)	< 0.001	1.49	5.56 (4.04 – 7.65)	< 0.001
Obesity	**MH – CO**	0.19	0.83 (0.11 – 6.12)	0.855	0.43	2.06 (0.64 – 6.59)	0.225
	**MH + CO**	0.74	2.79 (1.41 – 5.53)	0.003	1.13	5.06 (3.37 – 7.60)	< 0.001
	**MU – CO**	1.26	4.29 (2.62 – 7.04)	< 0.001	0.67	2.80 (1.42 – 5.53)	0.003
	**MU + CO**	2.34	7.33 (4.92 – 10.93)	< 0.001	2.30	8.97 (6.68 – 12.05)	< 0.001

†Adjusted for age, smoking, ethnicity, Townsend deprivation quintiles

† MH refers to metabolically healthy; MU refers to metabolically unhealthy; CO refers to central obesity.

Table S22 Relationship between BMI, central obesity, and metabolic health status on end-stage renal disease, stratified by sex.

BMI	Metabolic Health Status and Central Obesity†	By Sex					
		Male			Female		
		Event Rate (per 1,000 person-year)	HR (95% CI)	p-value	Event Rate (per 1,000 person-year)	HR (95% CI)	p-value
Normal BMI	MH – CO	0.11	Ref		0.04	Ref	
	MH + CO	0	-	-	0	-	-
	MU – CO	0.30	1.97 (1.01 – 3.86)	0.045	0.17	3.07 (1.46 – 6.45)	0.003
	MU + CO	0	-	-	0.30	4.71 (1.27 – 17.50)	0.021
Overweight	MH – CO	0.05	0.52 (0.18 – 1.49)	0.220	0.05	1.26 (0.42 – 3.77)	0.674
	MH + CO	0.26	2.01 (0.56 – 7.20)	0.285	0.08	1.74 (0.47 – 6.41)	0.408
	MU – CO	0.39	2.75 (1.47 – 5.12)	0.002	0.22	3.94 (1.88 – 8.24)	< 0.001
	MU + CO	0.63	3.93 (2.03 – 7.64)	< 0.001	0.28	4.48 (2.13 – 9.44)	< 0.001
Obesity	MH – CO	0	-	-	0	-	-
	MH + CO	0.12	1.06 (0.24 – 4.79)	0.938	0.13	2.92 (0.98 – 8.74)	0.055
	MU – CO	0.57	4.40 (2.09 – 9.26)	< 0.001	0.27	5.15 (1.58 – 16.78)	0.006
	MU + CO	0.82	5.63 (3.04 – 10.43)	< 0.001	0.48	7.99 (4.01 – 15.93)	< 0.001

†Adjusted for age, smoking, ethnicity, Townsend deprivation quintiles

† MH refers to metabolically healthy; MU refers to metabolically unhealthy; CO refers to central obesity.

Table S23 Relationship between BMI, central obesity, and metabolic health status on all-cause mortality, stratified by sex.

BMI	Metabolic Health Status and Central Obesity†	By Sex					
		Men			Women		
		Event Rate (per 1,000 person-year)	HR (95% CI)	p-value	Event Rate (per 1,000 person-year)	HR (95% CI)	p-value
Normal BMI	MH – CO	4.07	Ref		2.36	Ref	
	MH + CO	5.66	1.07 (0.15 – 7.60)	0.948	3.81	1.38 (0.86 – 2.20)	0.183
	MU – CO	7.61	1.32 (1.18 – 1.49)	< 0.001	4.83	1.35 (1.21 – 1.50)	< 0.001
	MU + CO	15.69	1.88 (0.93 – 3.78)	0.078	5.58	1.38 (1.04 – 1.83)	0.026
Overweight	MH – CO	3.22	0.87 (0.75 – 1.01)	0.069	2.46	1.01 (0.87 – 1.18)	0.884
	MH + CO	5.43	1.04 (0.80 – 1.36)	0.769	3.50	1.27 (1.05 – 1.53)	0.014
	MU – CO	6.01	1.13 (1.01 – 1.26)	0.037	4.18	1.19 (1.06 – 1.33)	0.004
	MU + CO	8.79	1.36 (1.20 – 1.55)	< 0.001	5.50	1.43 (1.27 – 1.61)	< 0.001
Obesity	MH – CO	4.21	1.49 (0.97 – 2.28)	0.071	2.58	1.17 (0.73 – 1.88)	0.503
	MH + CO	5.28	1.33 (1.05 – 1.69)	0.017	3.29	1.30 (1.07 – 1.58)	0.009
	MU – CO	6.18	1.36 (1.15 – 1.61)	< 0.001	3.12	1.00 (0.74 – 1.36)	0.977
	MU + CO	9.31	1.67 (1.49 – 1.87)	< 0.001	5.86	1.74 (1.57 – 1.93)	< 0.001

†Adjusted for age, smoking, ethnicity, Townsend deprivation quintiles
† MH refers to metabolically healthy; MU refers to metabolically unhealthy; CO refers to central obesity

Table S24 Relationship between BMI and the number of metabolic abnormalities on cardiovascular outcomes, stratified by sex.

BMI	Number of metabolic abnormalities	By Sex							
		Male				Female			
		Event Rate (per 1,000 person-year)	HR (95% CI)	p-value	p for trend	Event Rate (per 1,000 person-year)	HR (95% CI)	p-value	p for trend
**Composite of Incident ASCVD (CHD, ischemic stroke, PAD)**									
**Normal BMI**	**0**	6.37	Ref		< 0.001	2.91	Ref		< 0.001
	**1**	10.59	1.37 (1.24 – 1.52)	< 0.001		5.85	1.55 (1.40 – 1.72)	< 0.001	
	**2**	16.03	1.82 (1.62 – 2.05)	< 0.001		10.49	2.38 (2.10 – 2.70)	< 0.001	
	**3**	21.87	2.29 (1.77 – 2.97)	< 0.001		16.20	3.37 (2.36 – 4.82)	< 0.001	
**Overweight**	**0**	6.67	1.09 (0.98 – 1.22)	0.129	< 0.001	3.36	1.11 (0.98 – 1.24)	0.096	< 0.001
	**1**	10.61	1.46 (1.34 – 1.60)	< 0.001		6.22	1.66 (1.51 – 1.83)	< 0.001	
	**2**	15.87	1.90 (1.74 – 2.09)	< 0.001		10.56	2.45 (2.22 – 2.72)	< 0.001	
	**3**	23.88	2.60 (2.26 – 2.99)	< 0.001		15.05	3.25 (2.64 – 4.01)	< 0.001	
**Obesity**	**0**	8.47	1.46 (1.23 – 1.73)	< 0.001	< 0.001	4.00	1.34 (1.14 – 1.58)	< 0.001	< 0.001
	**1**	12.00	1.76 (1.59 – 1.96)	< 0.001		6.71	1.93 (1.73 – 2.14)	< 0.001	
	**2**	18.14	2.33 (2.12 – 2.56)	< 0.001		11.38	2.89 (2.62 – 3.19)	< 0.001	
	**3**	27.98	3.33 (2.96 – 3.75)	< 0.001		18.52	4.44 (3.86 – 5.11)	< 0.001	

**Coronary Heart Disease (CHD)**									
**Normal BMI**	**0**	3.75	Ref		< 0.001	1.34	Ref		< 0.001
	**1**	6.55	1.47 (1.29 – 1.67)	< 0.001		3.16	1.86 (1.61 – 2.14)	< 0.001	
	**2**	10.93	2.18 (1.88 – 2.52)	< 0.001		6.32	3.15 (2.66 – 3.74)	< 0.001	
	**3**	15.09	2.66 (1.95 – 3.62)	< 0.001		8.22	3.62 (2.22 – 5.91)	< 0.001	
**Overweight**	**0**	4.44	1.23 (1.07 – 1.41)	0.004	< 0.001	1.72	1.23 (1.04 – 1.46)	0.016	< 0.001
	**1**	7.25	1.72 (1.53 – 1.94)	< 0.001		3.67	2.16 (1.88 – 2.47)	< 0.001	
	**2**	11.22	2.36 (2.10 – 2.65)	< 0.001		7.26	3.73 (3.25 – 4.28)	< 0.001	
	**3**	17.64	3.33 (2.82 – 3.93)	< 0.001		10.88	5.09 (3.95 – 6.55)	< 0.001	
**Obesity**	**0**	6.00	1.73 (1.41 – 2.12)	< 0.001	< 0.001	2.40	1.77 (1.42 – 2.20)	< 0.001	< 0.001
	**1**	8.41	2.12 (1.86 – 2.41)	< 0.001		4.05	2.58 (2.23 – 2.98)	< 0.001	
	**2**	13.58	3.03 (2.69 – 3.41)	< 0.001		7.93	4.43 (3.87 – 5.06)	< 0.001	
	**3**	21.01	4.29 (3.72 – 4.95)	< 0.001		13.72	7.19 (6.03 – 8.57)	< 0.001	
**Myocardial Infarction**									
**Normal BMI**	**0**	1.42	Ref		< 0.001	0.45	Ref		< 0.001
	**1**	2.42	1.48 (1.20 – 1.83)	< 0.001		1.16	2.02 (1.59 – 2.57)	< 0.001	
	**2**	3.82	2.14 (1.69 – 2.72)	< 0.001		2.11	3.12 (2.33 – 4.18)	< 0.001	
	**3**	6.82	3.59 (2.29 – 5.64)	< 0.001		4.51	6.46 (3.36 – 12.42)	< 0.001	

**Overweight**	**0**	1.80	1.29 (1.04 – 1.61)	0.023	< 0.001	0.60	1.28 (0.96 – 1.71)	0.088	< 0.001
	**1**	2.97	1.89 (1.57 – 2.27)	< 0.001		1.26	2.22 (1.76 – 2.80)	< 0.001	
	**2**	4.17	2.42 (2.00 – 2.92)	< 0.001		2.29	3.46 (2.72 – 4.40)	< 0.001	
	**3**	6.13	3.39 (2.59 – 4.43)	< 0.001		2.92	4.05 (2.52 – 6.52)	< 0.001	
**Obesity**	**0**	2.19	1.63 (1.17 – 2.27)	0.004	< 0.001	0.69	1.54 (1.04 – 2.29)	0.031	< 0.001
	**1**	3.10	2.04 (1.66 – 2.51)	< 0.001		1.05	2.00 (1.53 – 2.60)	< 0.001	
	**2**	4.44	2.66 (2.19 – 3.22)	< 0.001		1.92	3.29 (2.58 – 4.19)	< 0.001	
	**3**	6.82	3.75 (2.96 – 4.75)	< 0.001		4.00	6.48 (4.75 – 8.84)	< 0.001	
**Strokes (All)**									
**Normal BMI**	**0**	2.03	Ref		< 0.001	1.18	Ref		< 0.001
	**1**	4.11	1.52 (1.28 – 1.81)	< 0.001		2.94	1.65 (1.42 – 1.92)	< 0.001	
	**2**	5.58	1.69 (1.39 – 2.07)	< 0.001		4.55	2.01 (1.67 – 2.44)	< 0.001	
	**3**	10.25	2.72 (1.87 – 3.98)	< 0.001		6.37	2.65 (1.55 – 4.55)	< 0.001	
**Overweight**	**0**	2.12	1.11 (0.92 – 1.35)	0.284	< 0.001	1.39	1.11 (0.93 – 1.34)	0.252	< 0.001
	**1**	3.45	1.38 (1.17 - 1.62)	< 0.001		2.94	1.65 (1.42 – 1.91)	< 0.001	
	**2**	4.85	1.58 (1.34 – 1.85)	< 0.001		4.39	1.97 (1.69 – 2.31)	< 0.001	
	**3**	8.04	2.26 (1.78 – 2.86)	< 0.001		6.02	2.56 (1.86 – 3.52)	< 0.001	

**Obesity**	**0**	2.23	1.23 (0.89 – 1.69)	0.205	< 0.001	1.39	1.14 (0.87 – 1.50)	0.331	< 0.001
	**1**	3.85	1.70 (1.42 – 2.03)	< 0.001		2.74	1.71 (1.45 – 2.02)	< 0.001	
	**2**	4.81	1.71 (1.45 – 2.02)	< 0.001		4.32	2.22 (1.90 – 2.60)	< 0.001	
	**3**	8.85	2.86 (2.33 – 3.50)	< 0.001		6.98	3.41 (2.74 – 4.25)	< 0.001	
**Ischemic Strokes**									
**Normal BMI**	**0**	0.91	Ref		< 0.001	0.37	Ref		< 0.001
	**1**	2.11	1.84 (1.43 – 2.37)	< 0.001		1.15	2.09 (1.62 – 2.71)	< 0.001	
	**2**	2.49	1.81 (1.35 – 2.45)	< 0.001		1.91	2.81 (2.06 – 3.83)	< 0.001	
	**3**	4.56	3.10 (1.78 – 5.39)	< 0.001		2.68	3.72 (1.62 – 8.53)	0.002	
**Overweight**	**0**	1.01	1.19 (0.89 – 1.58)	0.242	< 0.001	0.52	1.33 (0.97 – 1.81)	0.075	< 0.001
	**1**	1.72	1.61 (1.27 – 2.04)	< 0.001		1.19	2.18 (1.69 – 2.80)	< 0.001	
	**2**	2.37	1.86 (1.47 – 2.36)	< 0.001		1.87	2.76 (2.12 – 3.60)	< 0.001	
	**3**	3.85	2.70 (1.92 – 3.80)	< 0.001		2.50	3.55 (2.15 – 5.87)	< 0.001	
**Obesity**	**0**	1.30	1.61 (1.05 – 2.47)	0.028	0.0005	0.52	1.37 (0.87 – 2.15)	0.176	< 0.001
	**1**	1.93	1.96 (1.50 – 2.55)	< 0.001		1.30	2.65 (2.02 – 3.46)	< 0.001	
	**2**	2.26	1.92 (1.50 – 2.47)	< 0.001		1.94	3.27 (2.52 – 4.23)	< 0.001	
	**3**	4.11	3.22 (2.39 – 4.34)	< 0.001		3.06	4.94 (3.49 – 6.98)	< 0.001	

**Peripheral Artery Disease**									
**Normal BMI**	**0**	2.22	Ref		< 0.001	1.43	Ref		< 0.001
	**1**	3.23	1.15 (0.97 – 1.37)	0.115		2.15	1.15 (0.99 – 1.34)	0.069	
	**2**	5.03	1.52 (1.24 – 1.86)	< 0.001		3.61	1.63 (1.33 – 1.99)	< 0.001	
	**3**	7.16	2.13 (1.38 – 3.29)	< 0.001		6.40	2.84 (1.62 – 4.95)	< 0.001	
**Overweight**	**0**	1.93	0.91 (0.75 – 1.10)	0.314	< 0.001	1.44	0.97 (0.82 – 1.16)	0.748	0.002
	**1**	2.83	1.07 (0.91 – 1.25)	0.406		1.98	1.08 (0.93 – 1.26)	0.322	
	**2**	4.22	1.31 (1.12 – 1.54)	< 0.001		2.64	1.25 (1.05 – 1.48)	0.011	
	**3**	6.69	1.95 (1.53 – 2.49)	< 0.001		3.43	1.57 (1.05 – 2.35)	0.028	
**Obesity**	**0**	2.09	1.04 (0.75 – 1.43)	0.810	< 0.001	1.37	0.92 (0.71 – 1.21)	0.572	< 0.001
	**1**	2.87	1.16 (0.96 – 1.40)	0.123		1.93	1.10 (0.93 – 1.32)	0.265	
	**2**	4.57	1.57 (1.33 – 1.85)	< 0.001		2.69	1.37 (1.16 – 1.62)	< 0.001	
	**3**	7.29	2.34 (1.90 – 2.88)	< 0.001		4.30	2.05 (1.58 – 2.66)	< 0.001	
**Heart Failure**									
**Normal BMI**	**0**	1.28	Ref		< 0.001	0.55	Ref		< 0.001
	**1**	2.37	1.29 (1.04 – 1.61)	0.023		1.27	1.46 (1.18 – 1.82)	< 0.001	
	**2**	3.81	1.63 (1.28 – 2.09)	< 0.001		2.84	2.45 (1.90 – 3.15)	< 0.001	
	**3**	5.18	2.03 (1.22 – 3.38)	0.007		5.44	4.37 (2.41 – 7.92)	< 0.001	

**Overweight**	**0**	1.26	1.03 (0.81 – 1.32)	0.797	< 0.001	0.81	1.35 (1.05 – 1.74)	0.019	< 0.001
	**1**	2.46	1.48 (1.22 – 1.80)	< 0.001		1.54	1.76 (1.43 – 2.17)	< 0.001	
	**2**	3.95	1.91 (1.57 – 2.32)	< 0.001		2.82	2.54 (2.05 – 3.14)	< 0.001	
	**3**	7.18	3.02 (2.32 – 3.93)	< 0.001		5.23	4.21 (2.92 – 6.06)	< 0.001	
**Obesity**	**0**	1.90	1.63 (1.14 – 2.32)	0.007	< 0.001	1.04	1.71 (1.22 – 2.39)	0.002	< 0.001
	**1**	3.54	2.34 (1.89 – 2.88)	< 0.001		2.32	2.99 (2.42 – 3.69)	< 0.001	
	**2**	5.52	2.90 (2.38 – 3.52)	< 0.001		3.74	3.75 (3.06 – 4.60)	< 0.001	
	**3**	10.25	4.68 (3.75 – 5.85)	< 0.001		8.14	7.32 (5.73 – 9.36)	< 0.001	
**Cardiovascular Death**									
**Normal BMI**	**0**	0.92	Ref		< 0.001	0.38	Ref		< 0.001
	**1**	1.63	1.30 (1.00 – 1.69)	0.052		0.85	1.48 (1.13 – 1.94)	0.004	
	**2**	3.29	2.14 (1.62 – 2.83)	< 0.001		2.40	3.22 (2.40 – 4.33)	< 0.001	
	**3**	5.51	2.83 (1.65 – 4.85)	< 0.001		2.32	3.14 (1.27 – 7.76)	0.013	
**Overweight**	**0**	0.77	0.88 (0.65 – 1.20)	0.420	< 0.001	0.37	0.92 (0.65 – 1.30)	0.643	< 0.001
	**1**	1.71	1.48 (1.17 – 1.87)	0.001		0.94	1.64 (1.26 – 2.13)	< 0.001	
	**2**	2.17	1.52 (1.20 – 1.93)	< 0.001		1.30	1.84 (1.39 – 2.44)	< 0.001	
	**3**	4.88	2.95 (2.14 – 4.06)	< 0.001		3.24	4.25 (2.69 – 6.72)	< 0.001	

**Obesity**	**0**	1.23	1.49 (0.96 – 2.31)	0.074	< 0.001	0.75	1.86 (1.25 – 2.78)	0.002	< 0.001
	**1**	2.28	2.13 (1.66 – 2.76)	< 0.001		1.04	2.05 (1.54 – 2.71)	< 0.001	
	**2**	2.81	2.09 (1.64 – 2.67)	< 0.001		1.76	2.83 (2.17 – 3.68)	< 0.001	
	**3**	6.89	4.62 (3.54 – 6.04)	< 0.001		4.45	6.74 (4.91 – 9.26)	< 0.001	

†Adjusted for age, smoking, ethnicity, Townsend deprivation quintiles

Table S25 Relationship between BMI and the number of metabolic abnormalities on MASLD, stratified by sex.

BMI	Number of metabolic abnormalities	By Sex							
		Male				Female			
		Event Rate (per 1,000 person-year)	HR (95% CI)	p-value	p for trend	Event Rate (per 1,000 person-year)	HR (95% CI)	p-value	p for trend
Normal BMI	0	0.27	Ref		0.0057	0.22	Ref		< 0.001
	1	0.49	1.58 (0.98 – 2.56)	0.062		0.27	1.06 (0.71 – 1.59)	0.768	
	2	0.77	2.15 (1.25 – 3.71)	0.006		0.64	2.32 (1.45 – 3.73)	< 0.001	
	3	1.19	3.33 (1.16 – 9.59)	0.026		3.60	13.02 (6.15 – 27.57)	< 0.001	
Overweight	0	0.40	1.53 (0.93 – 2.51)	0.091	< 0.001	0.50	2.18 (1.52 – 3.12)	< 0.001	< 0.001
	1	0.71	2.47 (1.62 – 3.75)	< 0.001		0.83	3.26 (2.38 – 4.47)	< 0.001	
	2	1.11	3.40 (2.24 – 5.17)	< 0.001		1.25	4.57 (3.28 – 6.37)	< 0.001	
	3	2.68	7.52 (4.54 – 12.44)	< 0.001		2.68	9.01 (5.29 – 15.33)	< 0.001	
Obesity	0	0.60	2.36 (1.21 – 4.60)	0.011	< 0.001	1.02	4.48 (3.02 – 6.64)	< 0.001	< 0.001
	1	1.49	5.25 (3.44 – 8.03)	< 0.001		1.39	5.55 (4.05 – 7.60)	< 0.001	
	2	2.24	6.95 (4.62 – 10.45)	< 0.001		2.64	9.91 (7.33 – 13.40)	< 0.001	
	3	4.30	12.35 (7.96 – 19.14)	< 0.001		4.99	18.09 (12.74 – 25.70)	< 0.001	

[†]Adjusted for age, smoking, ethnicity, Townsend deprivation quintiles

Table S26 Relationship between BMI and the number of metabolic abnormalities on end-stage renal disease, stratified by sex.

BMI	Number of metabolic abnormalities	By Sex							
		Male				Female			
		Event Rate (per 1,000 person-year)	HR (95% CI)	p-value	p for trend	Event Rate (per 1,000 person-year)	HR (95% CI)	p-value	p for trend
Normal BMI	0	0.11	Ref		< 0.001	0.04	Ref		< 0.001
	1	0.14	1.06 (0.48 – 2.33)	0.890		0.10	2.01 (0.89 – 4.58)	0.095	
	2	0.66	4.18 (2.04 – 8.58)	< 0.001		0.40	6.82 (2.98 – 15.59)	< 0.001	
	3	0.90	4.16 (1.15 – 15.08)	0.030		1.79	23.16 (7.00 – 76.63)	< 0.001	
Overweight	0	0.08	0.71 (0.29 – 1.78)	0.468	< 0.001	0.06	1.45 (0.56 – 3.77)	0.441	< 0.001
	1	0.28	2.17 (1.14 – 4.16)	0.019		0.13	2.70 (1.25 – 5.84)	0.012	
	2	0.49	3.25 (1.71 – 6.18)	< 0.001		0.41	7.19 (3.44 – 15.04)	< 0.001	
	3	2.11	11.16 (5.55 – 22.45)	< 0.001		0.79	10.45 (3.65 – 29.92)	< 0.001	
Obesity	0	0.09	0.82 (0.18 – 3.70)	0.795	< 0.001	0.11	2.63 (0.88 – 7.84)	0.084	< 0.001
	1	0.39	3.14 (1.58 – 6.24)	0.0011		0.26	5.16 (2.42 – 10.97)	< 0.001	
	2	0.70	4.90 (2.58 – 9.29)	< 0.001		0.41	7.17 (3.43 – 14.97)	< 0.001	
	3	2.51	15.45 (8.05 – 29.65)	< 0.001		2.06	31.74 (15.03 – 67.04)	< 0.001	

†Adjusted for age, smoking, ethnicity, Townsend deprivation quintiles

Table S27 Relationship between BMI and the number of metabolic abnormalities on all-cause mortality, stratified by sex.

BMI	Number of metabolic abnormalities	By Sex							
		Men				Women			
		Event Rate (per 1,000 person-year)	HR (95% CI)	p-value	p for trend	Event Rate (per 1,000 person-year)	HR (95% CI)	p-value	p for trend
Normal BMI	**0**	4.07	Ref		< 0.001	2.39	Ref		< 0.001
	**1**	6.42	1.19 (1.05 – 1.36)	0.007		4.32	1.25 (1.12 – 1.40)	< 0.001	
	**2**	10.39	1.60 (1.38 – 1.85)	< 0.001		6.72	1.58 (1.36 – 1.83)	< 0.001	
	**3**	13.95	1.94 (1.40 – 2.68)	< 0.001		12.05	2.94 (1.98 – 4.37)	< 0.001	
Overweight	**0**	3.46	0.89 (0.77 – 1.03)	0.128	< 0.001	2.75	1.08 (0.95 – 1.23)	0.243	< 0.001
	**1**	5.70	1.13 (1.01 – 1.27)	0.037		4.15	1.22 (1.09 – 1.36)	< 0.001	
	**2**	7.32	1.19 (1.06 – 1.34)	0.004		5.61	1.37 (1.21 – 1.54)	< 0.001	
	**3**	11.11	1.68 (1.39 – 2.02)	< 0.001		8.37	1.96 (1.50 – 2.56)	< 0.001	
Obesity	**0**	5.00	1.36 (1.10 – 1.69)	0.005	< 0.001	3.18	1.27 (1.06 – 1.53)	0.011	< 0.001
	**1**	7.03	1.51 (1.32 – 1.72)	< 0.001		4.38	1.43 (1.27 – 1.62)	< 0.001	
	**2**	8.67	1.50 (1.33 – 1.69)	< 0.001		6.26	1.72 (1.53 – 1.93)	< 0.001	
	**3**	15.96	2.55 (2.20 – 2.96)	< 0.001		10.92	2.90 (2.44 – 3.43)	< 0.001	

†Adjusted for age, smoking, ethnicity, Townsend deprivation quintiles

Table S28 Interaction between sex and obesity, and its effect on ASCVD.

**Clinical Variables**[†]	**HR (95% CI)**	**P-value**
Female	0.54 (0.51 – 0.57)	< 0.001
Age	1.06 (1.05 – 1.06)	< 0.001
Obesity	1.23 (1.16 – 1.30)	< 0.001
1 metabolic abnormality	1.46 (1.38 – 1.55)	< 0.001
2 metabolic abnormalities	2.06 (1.93 – 2.20)	< 0.001
3 metabolic abnormalities	3.08 (2.82 – 3.36)	< 0.001
Female: Obesity*	1.08 (0.99 – 1.17)	0.071

[¥] The cox-proportional regression model generated above represents: ASCVD (Y) = α + β1 x Sex + β2 × BMI + β3 × Sex*BMI + β4 x metabolic abnormalities (covariate) + β5 x age (covariate)
[¥] Overweight individuals were excluded from this interaction analysis.
*Non-significant ($p > 0.05$)

Table S29 Interaction between sex and obesity, and its effect on coronary heart disease.

**Clinical Variables**[†]	**HR (95% CI)**	**P-value**
Female	0.46 (0.42 – 0.49)	< 0.001
Age	1.05 (1.04 – 1.05)	< 0.001
Obesity	1.34 (1.25 – 1.44)	< 0.001
1 metabolic abnormality	1.60 (1.48 – 1.73)	< 0.001
2 metabolic abnormalities	2.50 (2.30 – 2.72)	< 0.001
3 metabolic abnormalities	3.79 (3.40 – 4.22)	< 0.001
Female: Obesity	1.17 (1.06 – 1.29)	0.003

[¥] The cox-proportional regression model generated above represents: CHD (Y) = α + β1 x Sex + β2 × Obesity + β3 × Sex*Obesity + β4 x metabolic abnormalities (covariate) + β5 x age (covariate)
[¥] Overweight individuals were excluded from this interaction analysis.

Table S30 Interaction between sex and obesity, and its effect on ischaemic stroke.

Clinical Variables†	HR (95% CI)	P-value
Female	0.54 (0.47 – 0.62)	< 0.001
Age	1.08 (1.07 – 1.09)	< 0.001
Obesity	1.02 (0.89 – 1.17)	0.77
1 metabolic abnormality	1.90 (1.63 – 2.22)	< 0.001
2 metabolic abnormalities	2.10 (1.78 – 2.49)	< 0.001
3 metabolic abnormalities	3.35 (2.69 – 4.16)	< 0.001
Female: Obesity	1.33 (1.09 – 1.61)	0.004

¥ The cox-proportional regression model generated above represents: IS (Y) = α + β1 x Sex + β2 × Obesity + β3 × Sex*Obesity + β4 x metabolic abnormalities (covariate) + β5 x age (covariate)
¥ Overweight individuals were excluded from this interaction analysis.

Table S31 Interaction between sex and obesity, and its effect on heart failure.

Clinical Variables†	HR (95% CI)	P-value
Female	0.56 (0.49 – 0.63)	< 0.001
Age	1.10 (1.09 – 1.11)	< 0.001
Obesity	1.65 (1.47 – 1.84)	< 0.001
1 metabolic abnormality	1.53 (1.35 – 1.73)	< 0.001
2 metabolic abnormalities	2.05 (1.80 – 2.34)	< 0.001
3 metabolic abnormalities	3.64 (3.10 – 4.26)	< 0.001
Female: Obesity	1.19 (1.02 – 1.39)	0.031

¥ The cox-proportional regression model generated above represents: HF (Y) = α + β1 x Sex + β2 × Obesity + β3 × Sex*Obesity + β4 x metabolic abnormalities (covariate) + β5 x age (covariate)
¥ Overweight individuals were excluded from this interaction analysis.

Table S32 Interaction between sex and obesity, and its effect on cardiovascular death.

**Clinical Variables**†	**HR (95% CI)**	**P-value**
Female	0.52 (0.45 – 0.60)	< 0.001
Age	1.09 (1.08 – 1.10)	< 0.001
Obesity	1.25 (1.09 – 1.44)	0.001
1 metabolic abnormality	1.47 (1.26 – 1.72)	< 0.001
2 metabolic abnormalities	2.06 (1.75 – 2.43)	< 0.001
3 metabolic abnormalities	4.27 (3.51 – 5.21)	< 0.001
Female: Obesity*	1.07 (0.88 – 1.30)	0.50

¥ The cox-proportional regression model generated above represents: CV death (Y) = α + β1 x Sex + β2 × Obesity + β3 × Sex*Obesity + β4 x metabolic abnormalities (covariate) + β5 x age (covariate)
¥ Overweight individuals were excluded from this interaction analysis.
*Non-significant (p > 0.05)

Table S33 Interaction between sex and obesity, and its effect on MASLD.

**Clinical Variables**†	**HR (95% CI)**	**P-value**
Female	0.74 (0.57 – 0.96)	0.023
Age	1.03 (1.02 – 1.04)	< 0.001
Obesity	3.09 (2.48 – 3.85)	< 0.001
1 metabolic abnormality	1.41 (1.15 – 1.73)	< 0.001
2 metabolic abnormalities	2.31 (1.88 – 2.83)	< 0.001
3 metabolic abnormalities	4.38 (3.45 – 5.56)	< 0.001
Female: Obesity	1.49 (1.12 – 1.98)	0.007

¥ The cox-proportional regression model generated above represents: MASLD (Y) = α + β1 x Sex + β2 × Obesity + β3 × Sex*Obesity + β4 x metabolic abnormalities (covariate) + β5 x age (covariate)
¥ Overweight individuals were excluded from this interaction analysis.

Table S34 Interaction between sex and obesity, and its effect on end-stage renal disease.

**Clinical Variables**†	**HR (95% CI)**	**P-value**
Female	0.57 (0.38 – 0.86)	0.007
Age	1.04 (1.02 – 1.05)	< 0.001
Obesity	1.38 (1.04 – 1.82)	0.003
1 metabolic abnormality	2.13 (1.37 – 3.30)	< 0.001
2 metabolic abnormalities	4.40 (2.82 – 6.86)	< 0.001
3 metabolic abnormalities	15.43 (9.64 – 24.69)	< 0.001
Female: Obesity*	1.19 (0.74 – 1.89)	0.47

¥ The cox-proportional regression model generated above represents: ESRD (Y) = α + β1 x Sex + β2 × Obesity + β3 × Sex*Obesity + β4 x metabolic abnormalities (covariate) + β5 x age (covariate)
¥ Overweight individuals were excluded from this interaction analysis.
*Non-significant ($p > 0.05$)

Table S35 Interaction between sex and obesity, and its effect on all-cause mortality.

**Clinical Variables**†	**HR (95% CI)**	**P-value**
Female	0.63 (0.59 – 0.68)	< 0.001
Age	1.09 (1.08 – 1.09)	< 0.001
Obesity	1.14 (1.06 – 1.23)	< 0.001
1 metabolic abnormality	1.23 (1.14 – 1.32)	< 0.001
2 metabolic abnormalities	1.45 (1.34 – 1.57)	< 0.001
3 metabolic abnormalities	2.36 (2.12 – 2.63)	< 0.001
Female: Obesity*	1.03 (0.93 – 1.14)	0.57

¥ The cox-proportional regression model generated above represents: All-cause mortality (Y) = α + β1 x Sex + β2 × Obesity + β3 × Sex*Obesity + β4 x metabolic abnormalities (covariate) + β5 x age (covariate)
¥ Overweight individuals were excluded from this interaction analysis.
*Non-significant ($p > 0.05$)

Table S36 Interaction between sex and central obesity, and its effect on ASCVD.

**Clinical Variables†**	**HR (95% CI)**	**p-value**
Female	0.52 (0.49 – 0.54)	< 0.001
Age	1.06 (1.06 – 1.07)	< 0.001
Overweight	1.05 (1.01 – 1.10)	0.012
Central Obesity	1.13 (1.05 – 1.20)	< 0.001
Metabolically unhealthy (≥1 metabolic abnormality)	1.63 (1.56 – 1.71)	< 0.001
Female: Central Obesity	1.10 (1.01 – 1.21)	0.032

¥ The cox-proportional regression model generated above represents: ASCVD (Y) = α + β1 x Sex + β2 × Central Obesity + β3 × Sex*Central Obesity + β4 x metabolic health status + β5 x age (covariate)

¥ Individuals with obesity (BMI ≥30 kg/m^2) were excluded from this analysis.

¥ Metabolically unhealthy refers to having ≥1 metabolic abnormality

Table S37 Interaction between sex and central obesity, and its effect on heart failure.

**Clinical Variables†**	**HR (95% CI)**	**p-value**
Female	0.55 (0.50 – 0.60)	< 0.001
Age	1.13 (1.12 – 1.14)	< 0.001
Overweight	1.10 (1.02 – 1.20)	0.021
Central Obesity	1.25 (1.10 – 1.41)	< 0.001
Metabolically unhealthy (≥1 metabolic abnormality)	1.53 (1.39 – 1.70)	< 0.001
Female: Central Obesity*	1.12 (0.94 – 1.33)	0.20

¥ The cox-proportional regression model generated above represents: HF (Y) = α + β1 x Sex + β2 × Central Obesity + β3 × Sex*Central Obesity + β4 x metabolic health status + β5 x age (covariate)

¥ Individuals with obesity (BMI ≥30 kg/m^2) were excluded from this analysis.

¥ Metabolically unhealthy refers to having ≥1 metabolic abnormality

* Non-significant (p-value > 0.05)

Table S38 Interaction between sex and central obesity, and its effect on cardiovascular death.

**Clinical Variables**[†]	**HR (95% CI)**	**P-value**
Female	0.48 (0.43 – 0.54)	< 0.001
Age	1.11 (1.10 – 1.12)	< 0.001
Overweight	0.85 (0.76 – 0.94)	0.002
Central Obesity	1.41 (1.21 – 1.65)	< 0.001
Metabolically unhealthy (≥1 metabolic abnormality)	1.74 (1.53 – 1.98)	< 0.001
Female: Central Obesity*	1.00 (0.80 – 1.24)	0.97

[¥] The cox-proportional regression model generated above represents: CV death (Y) = α + β1 x Sex + β2 × Central Obesity + β3 × Sex*Central Obesity + β4 x metabolic health status + β5 x age (covariate)

[¥] Individuals with obesity (BMI ≥30 kg/m^2) were excluded from this analysis.

[¥] Metabolically unhealthy refers to having ≥1 metabolic abnormality

* Non-significant (p-value > 0.05)

Table S39 Interaction between sex and central obesity, and its effect on MASLD.

**Clinical Variables**[†]	**HR (95% CI)**	**P-value**
Female	0.74 (0.63 – 0.87)	< 0.001
Age	1.04 (1.03 – 1.05)	< 0.001
Overweight	1.59 (1.35 – 1.87)	< 0.001
Central Obesity	1.65 (1.33 – 2.04)	< 0.001
Metabolically unhealthy (≥1 metabolic abnormality)	1.62 (1.37 – 1.92)	< 0.001
Female: Central Obesity	1.46 (1.10 – 1.92)	0.008

[¥] The cox-proportional regression model generated above represents: MASLD (Y) = α + β1 x Sex + β2 × Central Obesity + β3 × Sex*Central Obesity + β4 x metabolic health status + β5 x age (covariate)

[¥] Individuals with obesity (BMI ≥30 kg/m^2) were excluded from this analysis.

[¥] Metabolically unhealthy refers to having ≥1 metabolic abnormality

Table S40 Interaction between sex and central obesity, and its effect on end-stage renal disease.

**Clinical Variables**†	**HR (95% CI)**	**P-value**
Female	0.53 (0.40 – 0.69)	< 0.001
Age	1.05 (1.04 – 1.07)	< 0.001
Overweight	1.24 (0.96 – 1.60)	0.10
Central Obesity	1.53 (1.10 – 2.13)	0.012
Metabolically unhealthy (≥1 metabolic abnormality)	3.19 (2.24 – 4.56)	< 0.001
Female: Central Obesity*	0.85 (0.52 – 1.41)	0.54

¥ The cox-proportional regression model generated above represents: ESRD (Y) = α + β1 x Sex + β2 × Central Obesity + β3 × Sex*Central Obesity + β4 x metabolic health status + β5 x age (covariate)

¥ Individuals with obesity (BMI ≥30 kg/m^2) were excluded from this analysis.

¥ Metabolically unhealthy refers to having ≥1 metabolic abnormality

* Non-significant (p-value > 0.05)

Table S41 Interaction between sex and central obesity, and its effect on all-cause mortality.

**Clinical Variables**†	**HR (95% CI)**	**P-value**
Female	0.64 (0.61 – 0.68)	< 0.001
Age	1.09 (1.09 – 1.10)	< 0.001
Overweight	0.87 (0.82 – 0.91)	< 0.001
Central Obesity	1.26 (1.16 – 1.37)	< 0.001
Metabolically unhealthy (≥1 metabolic abnormality)	1.27 (1.20 – 1.35)	< 0.001
Female: Central Obesity*	0.99 (0.89 – 1.11)	0.89

¥ The cox-proportional regression model generated above represents: all-cause mortality (Y) = α + β1 x Sex + β2 × Central Obesity + β3 × Sex*Central Obesity + β4 x metabolic health status + β5 x age (covariate)

¥ Individuals with obesity (BMI ≥30 kg/m^2) were excluded from this analysis.

¥ Metabolically unhealthy refers to having ≥1 metabolic abnormality

* Non-significant (p-value > 0.05)

Table S42 Interaction between sex and the number of metabolic abnormalities, and its effect on ASCVD.

**Clinical Variables**[†]	**HR (95% CI)**	**P-value**
Female	0.47 (0.42 – 0.51)	< 0.001
Age	1.06 (1.05 – 1.06)	< 0.001
Obesity	1.27 (1.21 – 1.33)	< 0.001
1 metabolic abnormality	1.35 (1.24 – 1.47)	< 0.001
2 metabolic abnormalities	1.81 (1.66 – 1.97)	< 0.001
3 metabolic abnormalities	2.65 (2.37 – 2.96)	< 0.001
Female: 1 metabolic abnormality	1.15 (1.03 – 1.30)	0.017
Female: 2 metabolic abnormalities	1.31 (1.16 – 1.47)	< 0.001
Female: 3 metabolic abnormalities	1.40 (1.19 – 1.65)	< 0.001

[¥] The cox-proportional regression model generated above represents: ASCVD (Y) = α + β1 x Sex + β2 × number of metabolic abnormalities + β3 × Sex*number of metabolic abnormalities + β4 x BMI (covariate) + β5 x age (covariate)

[¥] Overweight individuals were excluded from this interaction analysis.

Table S43 Interaction between sex and the number of metabolic abnormalities, and its effect on heart failure.

**Clinical Variables†**	**HR (95% CI)**	**P-value**
Female	0.48 (0.39 – 0.59)	< 0.001
Age	1.10 (1.09 – 1.11)	< 0.001
Obesity	1.78 (1.64 – 1.94)	< 0.001
1 metabolic abnormality	1.36 (1.14 – 1.63)	< 0.001
2 metabolic abnormalities	1.74 (1.46 – 2.08)	< 0.001
3 metabolic abnormalities	2.89 (2.35 – 3.55)	< 0.001
Female: 1 metabolic abnormality*	1.23 (0.96 – 1.57)	0.097
Female: 2 metabolic abnormalities	1.39 (1.09 – 1.77)	0.008
Female: 3 metabolic abnormalities	1.66 (1.24 – 2.23)	< 0.001

¥ The cox-proportional regression model generated above represents: HF (Y) = α + β1 x Sex + β2 × number of metabolic abnormalities + β3 × Sex*number of metabolic abnormalities + β4 x BMI (covariate) + β5 x age (covariate)

¥ Overweight individuals were excluded from this interaction analysis.

*Non-significant (p > 0.05)

Table S44 Sensitivity analysis of the relationship between BMI and metabolic health status (absence or presence of metabolic abnormalities) on cardiovascular outcomes, adjusting for biomarkers of metabolic health profile.

Metabolic Health Status	BMI	By Sex							
		Male				Female			
		Event Rate (per 1,000 person-year)	HR (95% CI)	p-value	p for trend	Event Rate (per 1,000 person-year)	HR (95% CI)	p-value	p for trend
**Composite of Incident ASCVD (AMI, ischemic stroke, PAD)**									
**Metabolically healthy**	**Normal BMI**	6.37	Ref		0.011	2.91	Ref		0.149
	**Overweight**	6.67	1.06 (0.94 – 1.21)	0.310		3.36	1.03 (0.90 – 1.18)	0.634	
	**Obesity**	8.47	1.38 (1.13 – 1.67)	0.001		4.00	1.21 (0.99 – 1.47)	0.062	
**Metabolically unhealthy**	**Normal BMI**	12.27	1.35 (1.21 – 1.51)	< 0.001	< 0.001	6.85	1.42 (1.27 – 1.60)	< 0.001	< 0.001
	**Overweight**	13.19	1.44 (1.30 – 1.61)	< 0.001		7.85	1.50 (1.35 – 1.69)	< 0.001	
	**Obesity**	16.64	1.76 (1.59 – 1.99)	< 0.001		9.54	1.78 (1.61 – 2.04)	< 0.001	
**Coronary Heart Disease**									
**Metabolically healthy**	**Normal BMI**	3.75	Ref		< 0.001	1.34	Ref		0.021
	**Overweight**	4.44	1.21 (1.04 – 1.43)	0.015		1.72	1.10 (0.90 – 1.33)	0.344	
	**Obesity**	6.00	1.72 (1.35 – 2.15)	< 0.001		2.40	1.60 (1.22 – 2.05)	< 0.001	
**Metabolically unhealthy**	**Normal BMI**	7.90	1.49 (1.30 – 1.73)	< 0.001	< 0.001	3.83	1.76 (1.50 – 2.06)	< 0.001	< 0.001
	**Overweight**	9.23	1.72 (1.52 – 1.99)	< 0.001		5.02	2.07 (1.77 – 2.42)	< 0.001	
	**Obesity**	12.25	2.15 (1.88 – 2.49)	< 0.001		6.40	2.57 (2.18 – 3.00)	< 0.001	

**Myocardial Infarction**									
**Metabolically healthy**	**Normal BMI**	1.42	Ref		0.595	0.45	Ref		0.542
	**Overweight**	1.80	1.16 (0.90 – 1.49)	0.263		0.60	1.02 (0.72 – 1.43)	0.927	
	**Obesity**	2.19	1.28 (0.87 – 1.90)	0.211		0.69	1.22 (0.76 – 1.96)	0.406	
**Metabolically unhealthy**	**Normal BMI**	2.90	1.28 (1.02 – 1.61)	0.036	0.029	1.38	1.54 (1.17 – 2.02)	0.002	0.786
	**Overweight**	3.58	1.47 (1.19 – 1.83)	< 0.001		1.64	1.59 (1.21 – 2.08)	< 0.001	
	**Obesity**	4.16	1.51 (1.20 – 1.90)	< 0.001		1.65	1.53 (1.15 – 2.03)	0.003	
**Strokes (All)**									
**Metabolically healthy**	**Normal BMI**	2.03	Ref		0.129	1.18	Ref		0.698
	**Overweight**	2.12	1.16 (0.92 – 1.44)	0.201		1.39	1.15 (0.94 – 1.42)	0.181	
	**Obesity**	2.23	1.09 (0.74 – 1.60)	0.628		1.39	0.97 (0.69 – 1.37)	0.857	
**Metabolically unhealthy**	**Normal BMI**	4.65	1.43 (1.18 – 1.74)	< 0.001	0.049	3.29	1.47 (1.24 – 1.75)	< 0.001	0.069
	**Overweight**	4.19	1.33 (1.10 – 1.61)	0.003		3.49	1.48 (1.25 – 1.77)	< 0.001	
	**Obesity**	4.86	1.59 (1.31 – 1.94)	< 0.001		3.73	1.64 (1.37 – 2.00)	< 0.001	
**Ischemic Strokes**									
**Metabolically healthy**	**Normal BMI**	0.91	Ref		0.067	0.37	Ref		0.405
	**Overweight**	1.01	1.12 (0.81 – 1.59)	0.539		0.52	1.28 (0.91 – 1.81)	0.158	
	**Obesity**	1.30	1.42 (0.86 – 2.41)	0.166		0.52	1.11 (0.64 – 1.91)	0.705	
**Metabolically unhealthy**	**Normal BMI**	2.28	1.59 (1.21 – 2.16)	0.001	0.728	1.31	1.56 (1.17 – 2.07)	0.003	0.055

	**Overweight**	2.06	1.43 (1.10 – 1.92)	0.008		1.44	1.51 (1.17 – 2.07)	0.005	
	**Obesity**	2.33	1.59 (1.22 – 2.19)	0.002		1.70	1.84 (1.36 – 2.47)	< 0.001	
**Peripheral Artery Disease**									
**Metabolically healthy**	**Normal BMI**	2.22	Ref		0.345	1.43	Ref		0.447
	**Overweight**	1.93	0.86 (0.69 – 1.07)	0.179		1.44	0.93 (0.75 – 1.14)	0.502	
	**Obesity**	2.09	0.86 (0.58 – 1.25)	0.455		1.37	0.85 (0.61 – 1.18)	0.321	
**Metabolically unhealthy**	**Normal BMI**	3.80	1.12 (0.93 – 1.36)	0.226	0.092	2.48	1.11 (0.93 – 1.32)	0.245	0.439
	**Overweight**	3.54	1.01 (0.85 – 1.21)	0.907		2.24	0.95 (0.79 – 1.13)	0.563	
	**Obesity**	4.18	1.22 (1.01 – 1.48)	0.038		2.34	1.04 (0.86 – 1.26)	0.710	
**Heart Failure**									
**Metabolically healthy**	**Normal BMI**	1.28	Ref		0.109	0.55	Ref		0.003
	**Overweight**	1.26	1.30 (0.98 – 1.73)	0.071		0.81	1.39 (0.99 – 1.79)	0.032	
	**Obesity**	1.90	1.67 (1.08 – 2.57)	0.021		1.04	1.88 (1.22 – 2.67)	0.002	
**Metabolically unhealthy**	**Normal BMI**	2.82	1.42 (1.09 – 1.79)	0.009	< 0.001	1.62	1.50 (1.18 – 1.93)	< 0.001	< 0.001
	**Overweight**	3.24	1.79 (1.41 – 2.25)	< 0.001		2.05	1.88 (1.49 – 2.40)	< 0.001	
	**Obesity**	5.23	2.73 (2.15 – 3.47)	< 0.001		3.37	3.02 (2.36 – 3.85)	< 0.001	
**Cardiovascular Death**									
**Metabolically healthy**	**Normal BMI**	0.92	Ref		0.790	0.38	Ref		0.425
	**Overweight**	0.77	0.87 (0.61 – 1.24)	0.441		0.37	0.77 (0.52 – 1.16)	0.218	

	**Obesity**	1.23	1.17 (0.68 – 2.01)	0.576		0.75	1.59 (0.98 – 2.58)	0.058	
**Metabolically unhealthy**	**Normal BMI**	2.16	1.35 (1.02 – 1.79)	0.039	< 0.001	1.16	1.35 (1.01 – 1.82)	0.050	0.002
	**Overweight**	2.02	1.31 (0.99 – 1.71)	0.052		1.12	1.28 (0.95 – 1.73)	0.103	
	**Obesity**	3.03	1.89 (1.43 – 2.50)	< 0.001		1.61	1.81 (1.33 – 2.46)	< 0.001	

†Adjusted for age, smoking, ethnicity, Townsend deprivation quintiles, systolic blood pressure, HbA1C, history of diabetes, HDL, LDL, and triglycerides.

Table S45 Sensitivity analysis of the relationship between BMI and metabolic health status (absence or presence of metabolic abnormalities) on MASLD, adjusting for biomarkers of metabolic health profile.

Metabolic Health Status	BMI	By Sex							
		Male				Female			
		Event Rate (per 1,000 person-year)	HR (95% CI)	p-value	p for trend	Event Rate (per 1,000 person-year)	HR (95% CI)	p-value	p for trend
Metabolically healthy	Normal BMI	0.27	Ref		< 0.001	0.22	Ref		< 0.001
	Overweight	0.40	1.67 (0.90 – 3.08)	0.101		0.50	2.02 (1.32 – 3.08)	0.001	
	Obesity	0.60	2.85 (1.27 – 6.42)	0.011		1.02	3.89 (2.41 – 6.29)	< 0.001	
Metabolically unhealthy	Normal BMI	0.58	2.04 (1.18 – 3.53)	0.010	< 0.001	0.38	1.18 (0.77 – 1.79)	0.441	< 0.001
	Overweight	0.95	3.63 (2.19 – 6.03)	< 0.001		1.02	2.62 (1.81 – 3.80)	< 0.001	
	Obesity	2.16	7.40 (4.44 – 12.34)	< 0.001		2.19	5.25 (3.63 – 7.60)	< 0.001	

†Adjusted for age, smoking, ethnicity, Townsend deprivation quintiles, systolic blood pressure, HbA1C, history of diabetes, HDL, LDL and triglycerides.

Table S46 Sensitivity analysis of the relationship between BMI and metabolic health status (absence or presence of metabolic abnormalities) on end-stage renal disease, adjusting for biomarkers of metabolic health profile.

Metabolic Health Status	BMI	By Sex							
		Male				Female			
		Event Rate (per 1,000 person-year)	HR (95% CI)	p-value	p for trend	Event Rate (per 1,000 person-year)	HR (95% CI)	p-value	p for trend
Metabolically healthy	Normal BMI	0.11	Ref		0.453	0.04	Ref		0.373
	Overweight	0.08	1.03 (0.30 – 3.56)	0.963		0.06	1.62 (0.57 – 4.64)	0.366	
	Obesity	0.09	1.92 (0.37 – 9.90)	0.438		0.11	2.11 (0.54 – 8.21)	0.281	
Metabolically unhealthy	Normal BMI	0.30	2.67 (1.03 – 6.94)	0.044	< 0.001	0.17	3.02 (1.29 – 7.06)	0.011	0.024
	Overweight	0.44	3.50 (1.39 – 8.79)	0.008		0.24	2.77 (1.19 – 6.47)	0.018	
	Obesity	0.78	5.36 (2.11 – 13.59)	< 0.001		0.46	4.59 (1.97 – 10.71)	< 0.001	

†Adjusted for age, smoking, ethnicity, Townsend deprivation quintiles, systolic blood pressure, HbA1C, history of diabetes, HDL, LDL and triglycerides.

Table S47 Sensitivity analysis of the relationship between BMI and metabolic health status (absence or presence of metabolic abnormalities) on all-cause mortality, adjusting for biomarkers of metabolic health profile.

Metabolic Health Status	BMI	By Sex							
		Men				Women			
		Event Rate (per 1,000 person-year)	HR (95% CI)	p-value	p for trend	Event Rate (per 1,000 person-year)	HR (95% CI)	p-value	p for trend
Metabolically healthy	Normal BMI	4.07	Ref		0.423	2.39	Ref		0.011
	Overweight	3.46	0.94 (0.79 – 1.11)	0.449		2.75	1.08 (0.93 – 1.26)	0.326	
	Obesity	5.00	1.25 (0.96 – 1.62)	0.093		3.18	1.28 (1.02 – 1.59)	0.031	
Metabolically unhealthy	Normal BMI	7.65	1.29 (1.13 – 1.50)	< 0.001	< 0.001	4.87	1.25 (1.10 – 1.43)	< 0.001	< 0.001
	Overweight	6.57	1.14 (1.01 – 1.32)	0.049		4.74	1.21 (1.06 – 1.38)	0.0045	
	Obesity	8.79	1.49 (1.29 – 1.72)	< 0.001		5.68	1.50 (1.31 – 1.72)	< 0.001	

†Adjusted for age, smoking, ethnicity, Townsend deprivation quintiles, systolic blood pressure, HbA1C, history of diabetes, HDL, LDL, and triglycerides.

Table S48 Sensitivity analysis of the relationship between BMI and metabolic health status on cardiovascular outcomes among men, adjusting for lifestyle and biological factors.

Men											
**Metabolic health status**	**BMI**	**Physical Activity**		**Alcohol Intake Frequency**		**Sleep Duration**		**hsCRP**		**ASI (≥ 10 m/s)**	
		**HR (95% CI)**	**p-value**	**HR (95% CI)**	**p-value**	**HR (95% CI)**	**p-value**	**HR (95% CI)**	**p-value**	**HR (95% CI)**	**p-value**
**Composite of ASCVD (CHD, Ischemic Stroke, PAD)**											
**Metabolically healthy**	**Normal BMI**	Ref		Ref		Ref		Ref		Ref	
	**Overweight**	1.10 (0.98 – 1.24)	0.107	1.11 (0.98 – 1.25)	0.096	1.11 (0.98 – 1.25)	0.097	1.08 (0.95 – 1.22)	0.246	1.07 (0.94 – 1.21)	0.316
	**Obesity**	1.47 (1.22 – 1.76)	< 0.001	1.46 (1.21 – 1.76)	< 0.001	1.47 (1.22 – 1.77)	< 0.001	1.37 (1.13 – 1.68)	0.002	1.35 (1.11 – 1.65)	0.003
**Metabolically unhealthy**	**Normal BMI**	1.49 (1.34 – 1.65)	< 0.001	1.49 (1.34 – 1.65)	< 0.001	1.49 (1.35 – 1.66)	< 0.001	1.46 (1.31 – 1.63)	< 0.001	1.45 (1.31 – 1.62)	< 0.001
	**Overweight**	1.72 (1.57 – 1.89)	< 0.001	1.72 (1.57 – 1.89)	< 0.001	1.73 (1.57 – 1.90)	< 0.001	1.67 (1.52 – 1.84)	< 0.001	1.64 (1.49 – 1.81)	< 0.001
	**Obesity**	2.25 (2.05 – 2.48)	< 0.001	2.24 (2.03 – 2.46)	< 0.001	2.24 (2.03 – 2.46)	< 0.001	2.11 (1.91– 2.33)	< 0.001	2.07 (1.87– 2.29)	< 0.001
**Coronary Heart Disease**											
**Metabolically healthy**	**Normal BMI**	Ref		Ref		Ref		Ref		Ref	
	**Overweight**	1.24 (1.07 – 1.45)	0.005	1.25 (1.07 – 1.45)	0.004	1.25 (1.07 – 1.45)	0.004	1.24 (1.06 – 1.45)	0.008	1.22 (1.04 – 1.43)	0.012
	**Obesity**	1.65 (1.31 – 2.07)	< 0.001	1.64 (1.31 – 2.06)	< 0.001	1.65 (1.31 – 2.07)	< 0.001	1.60 (1.25 – 2.04)	< 0.001	1.57 (1.23 – 2.01)	< 0.001
**Metabolically unhealthy**	**Normal BMI**	1.67 (1.47 – 1.91)	< 0.001	1.68 (1.47 – 1.91)	< 0.001	1.68 (1.47 – 1.92)	< 0.001	1.66 (1.45 – 1.91)	< 0.001	1.65 (1.44 – 1.90)	< 0.001
	**Overweight**	2.07 (1.83 – 2.33)	< 0.001	2.07 (1.83 – 2.33)	< 0.001	2.07 (1.84 – 2.34)	< 0.001	2.04 (1.80 – 2.31)	< 0.001	2.00 (1.76 – 2.26)	< 0.001
	**Obesity**	2.83 (2.51 – 3.20)	< 0.001	2.80 (2.48 – 3.16)	< 0.001	2.80 (2.47 – 3.16)	< 0.001	2.67 (2.35 – 3.04)	< 0.001	2.61 (2.29 – 2.97)	< 0.001

**Myocardial Infarction**											
**Metabolically healthy**	**Normal BMI**	Ref		Ref		Ref		Ref		Ref	
	**Overweight**	1.27 (1.00 – 1.61)	0.054	1.27 (1.00 – 1.61)	0.054	1.27 (1.00 – 1.61)	0.054	1.23 (0.95 – 1.57)	0.112	1.21 (0.94 – 1.56)	0.135
	**Obesity**	1.67 (1.17 – 2.39)	0.005	1.67 (1.17 – 2.39)	0.005	1.67 (1.17 – 2.39)	0.005	1.52 (1.03 – 2.24)	0.033	1.50 (1.02 – 2.20)	0.040
**Metabolically unhealthy**	**Normal BMI**	1.68 (1.36 – 2.07)	< 0.001	1.67 (1.35 – 2.07)	< 0.001	1.67 (1.35 – 2.07)	< 0.001	1.64 (1.32 – 2.05)	< 0.001	1.63 (1.31 – 2.03)	< 0.001
	**Overweight**	2.16 (1.78 – 2.61)	< 0.001	2.16 (1.78 – 2.61)	< 0.001	2.16 (1.78 – 2.61)	< 0.001	2.07 (1.70 – 2.53)	< 0.001	2.03 (1.66 – 2.47)	< 0.001
	**Obesity**	2.49 (2.05 – 3.04)	< 0.001	2.49 (2.05 – 3.04)	< 0.001	2.49 (2.05 – 3.04)	< 0.001	2.26 (1.84 – 2.78)	< 0.001	2.21 (1.79 – 2.72)	< 0.001
**All strokes**											
**Metabolically healthy**	**Normal BMI**	Ref		Ref		Ref		Ref		Ref	
	**Overweight**	1.03 (0.84 – 1.28)	0.750	1.04 (0.84 – 1.29)	0.704	1.04 (0.84 – 1.28)	0.740	0.95 (0.76 – 1.19)	0.655	0.95 (0.76 – 1.19)	0.661
	**Obesity**	1.27 (0.91 - 1.78)	0.162	1.29 (0.92 - 1.81)	0.143	1.29 (0.92 - 1.81)	0.139	1.13 (0.78 – 1.63)	0.509	1.13 (0.79 – 1.63)	0.505
**Metabolically unhealthy**	**Normal BMI**	1.52 (1.28 – 1.81)	< 0.001	1.52 (1.27 – 1.81)	< 0.001	1.52 (1.27 – 1.81)	< 0.001	1.45 (1.22 – 1.74)	< 0.001	1.46 (1.22 – 1.74)	< 0.001
	**Overweight**	1.48 (1.26 – 1.74)	< 0.001	1.49 (1.27 – 1.75)	< 0.001	1.49 (1.26 – 1.75)	< 0.001	1.38 (1.17 – 1.63)	< 0.001	1.38 (1.17 – 1.63)	< 0.001
	**Obesity**	1.80 (1.52 – 2.12)	< 0.001	1.80 (1.52 – 2.13)	< 0.001	1.79 (1.52 – 2.12)	< 0.001	1.64 (1.38 – 1.95)	< 0.001	1.64 (1.38 – 1.95)	< 0.001
**Ischemic Stroke**											
**Metabolically healthy**	**Normal BMI**	Ref		Ref		Ref		Ref		Ref	
	**Overweight**	1.13 (0.83 – 1.54)	0.449	1.13 (0.83 – 1.54)	0.431	1.12 (0.82 – 1.53)	0.474	1.01 (0.73 – 1.41)	0.939	1.01 (0.73 – 1.40)	0.939
	**Obesity**	1.78 (1.14 – 2.78)	0.011	1.79 (1.14 – 2.80)	0.011	1.80 (1.15 – 2.81)	0.010	1.62 (1.01 – 2.62)	0.049	1.62 (1.01 – 2.62)	0.049

**Metabolically unhealthy**	**Normal BMI**	1.77 (1.37 – 2.30)	< 0.001	1.76 (1.36 – 2.29)	< 0.001	1.76 (1.36 – 2.28)	< 0.001	1.72 (1.32 – 2.25)	< 0.001	1.72 (1.32 – 2.25)	< 0.001
	**Overweight**	1.78 (1.40 – 2.26)	< 0.001	1.78 (1.40 – 2.27)	< 0.001	1.78 (1.40 – 2.27)	< 0.001	1.65 (1.29 – 2.12)	< 0.001	1.65 (1.28 – 2.11)	< 0.001
	**Obesity**	2.09 (1.63 – 2.68)	< 0.001	2.09 (1.63 – 2.68)	< 0.001	2.08 (1.62 – 2.68)	< 0.001	1.92 (1.48 – 2.49)	< 0.001	1.90 (1.47 – 2.47)	< 0.001
**PAD**											
**Metabolically healthy**	**Normal BMI**	Ref		Ref		Ref		Ref		Ref	
	**Overweight**	0.94 (0.76 – 1.16)	0.568	0.94 (0.76 – 1.16)	0.592	0.95 (0.76 – 1.16)	0.625	0.91 (0.73 – 1.13)	0.625	0.90 (0.72 – 1.13)	0.368
	**Obesity**	1.23 (0.88 – 1.72)	0.224	1.24 (0.88 – 1.72)	0.211	1.24 (0.88 – 1.72)	0.199	1.11 (0.77 – 1.59)	0.199	1.11 (0.77 – 1.59)	0.591
**Metabolically unhealthy**	**Normal BMI**	1.26 (1.06 – 1.51)	0.010	1.25 (1.05 – 1.50)	0.012	1.26 (1.05 – 1.50)	0.010	1.22 (1.01 – 1.47)	0.034	1.22 (1.01 – 1.47)	0.037
	**Overweight**	1.25 (1.07 – 1.47)	0.006	1.25 (1.07 – 1.47)	0.006	1.26 (1.07 – 1.47)	0.005	1.20 (1.02 – 1.42)	0.031	1.19 (1.01 – 1.42)	0.039
	**Obesity**	1.56 (1.32 – 1.84)	< 0.001	1.56 (1.32 – 1.84)	< 0.001	1.57 (1.32 – 1.84)	< 0.001	1.44 (1.21 – 1.72)	< 0.001	1.43 (1.20 – 1.70)	< 0.001
**Heart Failure**											
**Metabolically healthy**	**Normal BMI**	Ref		Ref		Ref		Ref		Ref	
	**Overweight**	1.10 (0.84 – 1.44)	0.479	1.11 (0.85 – 1.46)	0.435	1.14 (0.87 – 1.49)	0.357	1.17 (0.89 – 1.54)	0.289	1.16 (0.87 – 1.54)	0.302
	**Obesity**	1.88 (1.28 – 2.74)	0.0012	1.89 (1.29 – 2.76)	0.0011	1.93 (1.32 – 2.82)	0.0008	1.62 (1.06 – 2.48)	0.026	1.61 (1.05 – 2.47)	0.027
**Metabolically unhealthy**	**Normal BMI**	1.45 (1.16 – 1.82)	0.0012	1.45 (1.16 – 1.82)	0.0011	1.48 (1.16 – 1.82)	< 0.001	1.49 (1.17 – 1.89)	0.001	1.49 (1.17 – 1.89)	0.001
	**Overweight**	1.84 (1.50 – 2.26)	< 0.001	1.85 (1.50 – 2.26)	< 0.001	1.88 (1.53 – 2.31)	< 0.001	1.84 (1.48 – 2.29)	< 0.001	1.83 (1.47 – 2.28)	< 0.001
	**Obesity**	3.03 (2.47 – 3.72)	< 0.001	3.02 (2.45 – 3.71)	< 0.001	3.07 (2.49 – 3.78)	< 0.001	2.86 (2.29 – 3.57)	< 0.001	2.84 (2.27 – 3.54)	< 0.001

Cardiovascular Death											
**Metabolically healthy**	**Normal BMI**	Ref		Ref		Ref		Ref		Ref	
	**Overweight**	0.89 (0.64 – 1.24)	0.497	0.90 (0.65 – 1.25)	0.519	0.90 (0.65 – 1.25)	0.521	0.87 (0.62 – 1.23)	0.425	0.86 (0.61 – 1.21)	0.377
	**Obesity**	1.42 (0.88 – 2.31)	0.152	1.43 (0.88 – 2.32)	0.151	1.45 (0.89 – 2.35)	0.133	1.42 (0.86 – 2.33)	0.172	1.39 (0.85 – 2.29)	0.193
**Metabolically unhealthy**	**Normal BMI**	1.53 (1.18 – 1.99)	0.001	1.54 (1.18 – 1.99)	0.001	1.55 (1.19 – 2.01)	0.001	1.49 (1.14 – 1.96)	0.004	1.48 (1.13 – 1.94)	0.005
	**Overweight**	1.56 (1.23 – 1.99)	< 0.001	1.56 (1.23 – 1.99)	< 0.001	1.58 (1.24 – 2.01)	< 0.001	1.45 (1.13 – 1.86)	0.004	1.41 (1.10 – 1.82)	0.007
	**Obesity**	2.25 (1.76 – 2.88)	< 0.001	2.22 (1.73 – 2.84)	< 0.001	2.25 (1.76 – 2.88)	< 0.001	1.92 (1.49 – 2.49)	< 0.001	1.88 (1.45 – 2.43)	< 0.001

†Main model was adjusted for age, smoking, ethnicity, Townsend deprivation quintiles.

Table S49 Sensitivity analysis of the relationship between BMI and metabolic health status on cardiovascular outcomes among women, adjusting for lifestyle and biological factors.

Women											
Metabolic Health Status	BMI	Physical Activity		Alcohol Intake Frequency		Sleep Duration		hs-CRP		ASI (≥ 10 m/s)	
		HR (95% CI)	p-value	HR (95% CI)	p-value	HR (95% CI)	p-value	HR (95% CI)	p-value	HR (95% CI)	p-value
**Composite of ASCVD (CHD, Ischemic Stroke, PAD)**											
**Metabolically healthy**	**Normal BMI**	Ref		Ref		Ref		Ref		Ref	
	**Overweight**	1.13 (0.99 – 1.29)	0.106	1.12 (0.99 – 1.28)	0.083	1.13 (0.99 – 1.28)	0.076	1.04 (0.91 – 1.20)	0.563	1.04 (0.91 – 1.19)	0.574
	**Obesity**	1.29 (1.07 – 1.56)	0.007	1.26 (1.04 – 1.52)	0.017	1.26 (1.04 – 1.52)	0.017	1.11 (0.90 – 1.36)	0.323	1.11 (0.90 – 1.36)	0.338
**Metabolically unhealthy**	**Normal BMI**	1.73 (1.56 – 1.92)	< 0.001	1.71 (1.54 – 1.90)	< 0.001	1.71 (1.54 – 1.89)	< 0.001	1.62 (1.45 – 1.81)	< 0.001	1.62 (1.45 – 1.80)	< 0.001
	**Overweight**	1.89 (1.71 – 2.09)	< 0.001	1.85 (1.68 – 2.05)	< 0.001	1.85 (1.68 – 2.05)	< 0.001	1.70 (1.53 – 1.89)	< 0.001	1.70 (1.53 – 1.88)	< 0.001
	**Obesity**	2.45 (2.21 – 2.70)	< 0.001	2.33 (2.10 – 2.57)	< 0.001	2.32 (2.10 – 2.57)	< 0.001	2.02 (1.81 – 2.25)	< 0.001	2.01 (1.80 – 2.24)	< 0.001
**Coronary Heart Disease**											
**Metabolically healthy**	**Normal BMI**	Ref		Ref		Ref		Ref		Ref	
	**Overweight**	1.18 (0.97 – 1.42)	0.093	1.16 (0.96 – 1.40)	0.124	1.17 (0.96 – 1.41)	0.113	1.02 (0.83 – 1.24)	0.883	1.01 (0.83 – 1.24)	0.899
	**Obesity**	1.73 (1.35 – 2.21)	< 0.001	1.65 (1.29 – 2.11)	< 0.001	1.66 (1.29 – 2.12)	< 0.001	1.47 (1.13 – 1.91)	< 0.001	1.46 (1.12 – 1.90)	< 0.001
**Metabolically unhealthy**	**Normal BMI**	2.09 (1.80 – 2.42)	< 0.001	2.07 (1.79 – 2.40)	< 0.001	2.07 (1.79 – 2.40)	< 0.001	1.92 (1.65 – 2.23)	< 0.001	1.91 (1.65 – 2.23)	< 0.001
	**Overweight**	2.57 (2.24 – 2.96)	< 0.001	2.49 (2.17 – 2.86)	< 0.001	2.50 (2.17 – 2.87)	< 0.001	2.25 (1.95 – 2.60)	0.004	2.24 (1.93 – 2.58)	0.005
	**Obesity**	3.53 (3.07 – 4.06)	< 0.001	3.29 (2.86 – 3.78)	< 0.001	3.28 (2.85 – 3.78)	< 0.001	2.77 (2.39 – 3.22)	< 0.001	2.75 (2.36 – 3.20)	< 0.001

**Myocardial Infarction**											
**Metabolically healthy**	**Normal BMI**	Ref		Ref		Ref		Ref		Ref	
	**Overweight**	1.07 (0.77 – 1.49)	0.679	1.05 (0.76 – 1.47)	0.753	1.06 (0.76 – 1.47)	0.750	0.92 (0.65 – 1.30)	0.627	0.91 (0.64 – 1.29)	0.604
	**Obesity**	1.66 (1.09 – 2.54)	0.019	1.57 (1.03 – 2.41)	0.037	1.57 (1.03 – 2.40)	0.038	1.47 (0.94 – 2.30)	0.088	1.44 (0.93 – 2.25)	0.105
**Metabolically unhealthy**	**Normal BMI**	2.11 (1.64 – 2.71)	< 0.001	2.09 (1.63 – 2.69)	< 0.001	2.09 (1.63 – 2.68)	< 0.001	1.91 (1.48 – 2.47)	< 0.001	1.89 (1.46 – 2.44)	< 0.001
	**Overweight**	2.48 (1.96 – 3.15)	< 0.001	2.39 (1.88 – 3.03)	< 0.001	2.39 (1.88 – 3.03)	< 0.001	2.17 (1.70 – 2.78)	< 0.001	2.12 (1.66 – 2.72)	< 0.001
	**Obesity**	2.60 (2.04 – 3.33)	< 0.001	2.39 (1.87 – 3.06)	< 0.001	2.39 (1.86 – 3.06)	< 0.001	2.03 (1.56 – 2.65)	< 0.001	1.97 (1.51 – 2.57)	< 0.001
**All strokes**											
**Metabolically healthy**	**Normal BMI**	Ref		Ref		Ref		Ref		Ref	
	**Overweight**	1.18 (0.96 – 1.46)	0.114	1.18 (0.96 – 1.46)	0.123	1.18 (0.95 – 1.45)	0.133	1.20 (0.96 – 1.49)	0.104	1.20 (0.96 – 1.49)	0.106
	**Obesity**	1.13 (0.82 – 1.56)	0.452	1.10 (0.80 – 1.52)	0.553	1.10 (0.80 – 1.52)	0.553	0.96 (0.67 – 1.38)	0.821	0.96 (0.67 – 1.37)	0.807
**Metabolically unhealthy**	**Normal BMI**	1.84 (1.56 – 2.17)	< 0.001	1.83 (1.55 – 2.15)	< 0.001	1.82 (1.55 – 2.15)	< 0.001	1.77 (1.49 – 2.10)	< 0.001	1.77 (1.49 – 2.10)	< 0.001
	**Overweight**	1.89 (1.61 – 2.21)	< 0.001	1.85 (1.58 – 2.17)	< 0.001	1.86 (1.59 – 2.18)	< 0.001	1.78 (1.50 – 2.10)	< 0.001	1.77 (1.50 – 2.09)	< 0.001
	**Obesity**	2.19 (1.87 – 2.58)	< 0.001	2.09 (1.78 – 2.46)	< 0.001	2.09 (1.77 – 2.46)	< 0.001	1.97 (1.65 – 2.35)	< 0.001	1.96 (1.64 – 2.34)	< 0.001
**Ischemic Stroke**											
**Metabolically healthy**	**Normal BMI**	Ref		Ref		Ref		Ref		Ref	
	**Overweight**	1.66 (1.18 – 2.35)	0.004	1.66 (1.18 – 2.35)	0.004	1.67 (1.18 – 2.36)	0.004	1.54 (1.08 – 2.20)	0.018	1.53 (1.07 – 2.19)	0.018
	**Obesity**	1.53 (0.92 – 2.56)	0.103	1.52 (0.91 – 2.54)	0.113	1.52 (0.91 – 2.54)	0.113	1.52 (0.68 – 2.14)	0.514	1.20 (0.68 – 2.12)	0.527

**Metabolically unhealthy**	**Normal BMI**	2.41 (1.81 – 3.21)	< 0.001	2.38 (1.79 – 3.17)	< 0.001	2.38 (1.79 – 3.17)	< 0.001	2.16 (1.62 – 2.90)	< 0.001	2.16 (1.62 – 2.89)	< 0.001
	**Overweight**	2.61 (1.98 – 3.44)	< 0.001	2.58 (1.96 – 3.40)	< 0.001	2.59 (1.96 – 3.41)	< 0.001	2.29 (1.73 – 3.04)	< 0.001	2.28 (1.72 – 3.02)	< 0.001
	**Obesity**	3.23 (2.44 – 4.28)	< 0.001	3.13 (2.36 – 4.14)	< 0.001	3.12 (2.36 – 4.14)	< 0.001	2.68 (1.99 – 3.60)	< 0.001	2.66 (1.98 – 3.58)	< 0.001
**Peripheral Artery Disease**											
**Metabolically healthy**	**Normal BMI**	Ref		Ref		Ref		Ref		Ref	
	**Overweight**	0.99 (0.82 – 1.20)	0.917	0.99 (0.82 – 1.20)	0.903	0.99 (0.82 – 1.20)	0.940	0.96 (0.78 – 1.17)	0.660	0.96 (0.78 – 1.17)	0.666
	**Obesity**	0.83 (0.60 – 1.14)	0.244	0.82 (0.59 – 1.12)	0.210	0.82 (0.59 – 1.12)	0.216	0.76 (0.48 – 1.02)	0.057	0.76 (0.48 – 1.03)	0.060
**Metabolically unhealthy**	**Normal BMI**	1.25 (1.07 – 1.46)	0.006	1.24 (1.06 – 1.45)	0.008	1.24 (1.06 – 1.45)	0.008	1.20 (1.02 – 1.42)	0.030	1.20 (1.02 – 1.42)	0.030
	**Overweight**	1.12 (0.96 – 1.30)	0.152	1.10 (0.95 – 1.29)	0.204	1.11 (0.95 – 1.29)	0.185	1.01 (0.86 – 1.19)	0.893	1.01 (0.86 – 1.19)	0.871
	**Obesity**	1.22 (1.04 – 1.43)	0.014	1.18 (1.01 – 1.39)	0.043	1.19 (1.01 – 1.39)	0.038	1.01 (0.85 – 1.21)	0.900	1.01 (0.85 – 1.21)	0.900
**Heart Failure**											
**Metabolically healthy**	**Normal BMI**	Ref		Ref		Ref		Ref		Ref	
	**Overweight**	1.26 (0.94 – 1.68)	0.118	1.26 (0.94 – 1.68)	0.122	1.26 (0.94 – 1.68)	0.117	1.28 (0.94 – 1.73)	0.115	1.26 (0.94 – 1.68)	0.115
	**Obesity**	1.56 (1.05 – 2.32)	0.028	1.52 (1.03 – 2.27)	0.037	1.53 (1.03 – 2.27)	0.036	1.49 (0.98 – 2.28)	0.062	1.53 (1.03 – 2.28)	0.035
**Metabolically unhealthy**	**Normal BMI**	1.68 (1.33 – 2.11)	< 0.001	1.67 (1.33 – 2.10)	< 0.001	1.66 (1.32 – 2.09)	< 0.001	1.64 (1.28 – 2.09)	< 0.001	1.66 (1.32 – 2.10)	< 0.001
	**Overweight**	1.99 (1.60 – 2.47)	< 0.001	1.95 (1.57 – 2.42)	< 0.001	1.95 (1.57 – 2.43)	< 0.001	1.89 (1.50 – 2.39)	< 0.001	1.89 (1.50 – 2.39)	< 0.001
	**Obesity**	3.49 (2.82 – 4.32)	< 0.001	3.32 (2.68 – 4.12)	< 0.001	3.33 (2.69 – 4.13)	< 0.001	3.04 (2.40 – 3.84)	< 0.001	3.34 (2.69 – 4.15)	< 0.001

**Cardiovascular Death**											
**Metabolically healthy**	**Normal BMI**	Ref		Ref		Ref		Ref		Ref	
	**Overweight**	1.14 (0.78 – 1.67)	0.507	1.12 (0.77 – 1.65)	0.556	1.12 (0.77 – 1.65)	0.547	1.03 (0.69 – 1.55)	0.547	1.03 (0.68 – 1.55)	0.892
	**Obesity**	1.92 (1.20 – 3.08)	0.007	1.80 (1.13 – 2.89)	0.014	1.81 (1.13 – 2.90)	0.014	1.59 (0.95 – 2.67)	0.0143	1.58 (0.95 – 2.65)	0.081
**Metabolically unhealthy**	**Normal BMI**	1.95 (1.46 – 2.62)	< 0.001	1.94 (1.45 – 2.59)	< 0.001	1.92 (1.44 – 2.58)	< 0.001	1.83 (1.35 – 2.49)	< 0.001	1.83 (1.35 – 2.48)	< 0.001
	**Overweight**	1.75 (1.32 – 2.34)	< 0.001	1.69 (1.27 – 2.25)	< 0.001	1.69 (1.27 – 2.25)	< 0.001	1.61 (1.19 – 2.17)	< 0.001	1.60 (1.18 – 2.16)	0.002
	**Obesity**	2.81 (2.12 – 3.73)	< 0.001	2.57 (1.93 – 3.41)	< 0.001	2.57 (1.93 – 3.43)	< 0.001	2.26 (1.66 – 3.09)	< 0.001	2.25 (1.65 – 3.07)	< 0.001

†Main model was adjusted for age, smoking, ethnicity, Townsend deprivation quintiles.

Table S50 Sensitivity analysis of the relationship between BMI and metabolic health status on MASLD among men, adjusting for lifestyle and biological factors.

	Male										
**Metabolic health status**	**BMI**	**Physical Activity**		**Alcohol Intake Frequency**		**Sleep Duration**		**hs-CRP**		**ASI (≥ 10 m/s)**	
		**HR (95% CI)**	**p-value**	**HR (95% CI)**	**p-value**	**HR (95% CI)**	**p-value**	**HR (95% CI)**	**p-value**	**HR (95% CI)**	**p-value**
**Metabolic healthy**	**Normal BMI**	Ref		Ref		Ref		Ref		Ref	
	**Overweight**	1.51 (0.87 – 2.63)	0.140	1.54 (0.89 – 2.66)	0.127	1.54 (0.89 – 2.67)	0.125	1.45 (0.81 – 2.60)	0.211	1.45 (0.81 – 2.60)	0.209
	**Obesity**	2.30 (1.08 – 4.88)	0.030	2.38 (1.12 – 5.06)	0.024	2.39 (1.13 – 5.08)	0.023	2.26 (1.02 – 5.01)	0.044	2.27 (1.02 – 5.02)	0.043
**Metabolic unhealthy**	**Normal BMI**	1.71 (1.04 – 2.82)	0.035	1.69 (1.03 – 2.79)	0.039	1.69 (1.02 – 2.78)	0.040	1.69 (1.00 – 2.84)	0.050	1.69 (1.01 – 2.85)	0.049
	**Overweight**	3.09 (1.98 – 4.83)	< 0.001	3.11 (1.99 – 4.86)	< 0.001	3.11 (1.99 – 4.85)	< 0.001	2.97 (1.86 – 4.75)	< 0.001	2.98 (1.87 – 4.76)	< 0.001
	**Obesity**	7.35 (4.72 – 11.43)	< 0.001	7.44 (4.78 – 11.58)	< 0.001	7.43 (4.78 – 11.56)	< 0.001	6.50 (4.07 – 10.38)	< 0.001	6.52 (4.08 – 10.42)	< 0.001

†Main model was adjusted for age, smoking, ethnicity, Townsend deprivation quintiles.

Table S51 Sensitivity analysis of the relationship between BMI and metabolic health status on MASLD among women, adjusting for lifestyle and biological factors.

		Female									
**Metabolic health status**	**BMI**	**Physical Activity**		**Alcohol Intake Frequency**		**Sleep Duration**		**hs-CRP**		**ASI (≥ 10 m/s)**	
		**HR (95% CI)**	**p-value**	**HR (95% CI)**	**p-value**	**HR (95% CI)**	**p-value**	**HR (95% CI)**	**p-value**	**HR (95% CI)**	**p-value**
**Metabolic healthy**	**Normal BMI**	Ref		Ref		Ref		Ref		Ref	
	**Overweight**	2.15 (1.45 – 3.17)	< 0.001	2.17 (1.46 – 3.21)	< 0.001	2.12 (1.44 – 3.15)	< 0.001	1.79 (1.18 – 2.72)	0.006	1.78 (1.17 – 2.70)	0.007
	**Obesity**	4.45 (2.89 – 6.84)	< 0.001	4.34 (2.81 – 6.69)	< 0.001	4.32 (2.80 – 6.66)	< 0.001	2.93 (1.81 – 4.74)	< 0.001	2.87 (1.78 – 4.65)	< 0.001
**Metabolic unhealthy**	**Normal BMI**	1.34 (0.91 – 1.97)	0.142	1.33 (0.90 – 1.96)	0.157	1.32 (0.89 – 1.96)	0.160	1.19 (0.79 – 1.80)	0.394	1.18 (0.79 – 1.78)	0.424
	**Overweight**	3.30 (2.37 – 4.57)	< 0.001	3.26 (2.34 – 4.53)	< 0.001	3.24 (2.33 – 4.52)	< 0.001	2.75 (1.94 – 3.89)	< 0.001	2.68 (1.90 – 3.79)	< 0.001
	**Obesity**	7.30 (5.33 – 10.01)	< 0.001	6.89 (5.00 – 9.50)	< 0.001	6.89 (5.00 – 9.50)	< 0.001	4.94 (3.49 – 6.98)	< 0.001	4.79 (3.39 – 6.77)	< 0.001

†Main model was adjusted for age, smoking, ethnicity, Townsend deprivation quintiles.

Table S52 Sensitivity analysis of the relationship between BMI and metabolic health status on end-stage renal disease among men, adjusting for lifestyle and biological factors.

		Male											
**Metabolic Health Status**	**BMI**	**Physical Activity**		**Alcohol Intake Frequency**		**Sleep Duration**		**hs-CRP**		**eGFR**		**ASI (≥ 10 m/s)**	
		**HR (95% CI)**	**p-value**	**HR (95% CI)**	**p-value**	**HR (95% CI)**	**p-value**	**HR (95% CI)**	**p-value**	**HR (95% CI)**	**p-value**	**HR (95% CI)**	**p-value**
**Metabolic healthy**	**Normal BMI**	Ref		Ref		Ref		Ref		Ref		Ref	
	**Overweight**	0.98 (0.37 – 2.61)	0.969	0.99 (0.37 – 2.65)	0.990	1.00 (0.37 – 2.66)	0.994	0.97 (0.36 – 2.59)	0.954	0.68 (0.25 – 1.89)	0.464	0.69 (0.25 – 1.90)	0.471
	**Obesity**	1.14 (0.24 – 5.35)	0.872	1.13 (0.24 – 5.32)	0.879	1.14 (0.24 – 5.36)	0.871	1.09 (0.23 – 5.15)	0.913	0.93 (0.20 – 4.42)	0.932	0.94 (0.20 – 4.45)	0.939
**Metabolic unhealthy**	**Normal BMI**	2.04 (0.93 – 4.47)	0.074	1.99 (0.91 – 4.37)	0.086	1.98 (0.90 – 4.36)	0.088	1.67 (0.75 – 3.71)	0.205	1.44 (0.65 – 3.20)	0.372	1.44 (0.65 – 3.21)	0.367
	**Overweight**	3.42 (1.67 – 7.00)	< 0.001	3.43 (1.67 – 7.03)	< 0.001	3.42 (1.67 – 7.02)	< 0.001	2.78 (1.35 – 5.72)	0.006	1.94 (0.94 – 4.00)	0.075	1.96 (0.95 – 4.04)	0.070
	**Obesity**	5.62 (2.74 – 11.55)	< 0.001	5.51 (2.68 – 11.32)	< 0.001	5.52 (2.68 – 11.34)	< 0.001	4.23 (2.04 – 8.76)	< 0.001	2.89 (1.39 – 6.01)	0.005	2.93 (1.41 – 6.10)	0.004

†Main model was adjusted for age, smoking, ethnicity, Townsend deprivation quintiles.

Table S53 Sensitivity analysis of the relationship between BMI and metabolic health status on end-stage renal disease among women, adjusting for lifestyle and biological factors.

Female													
Metabolic health status	BMI	Physical Activity		Alcohol Intake Frequency		Sleep Duration		hs-CRP		eGFR		ASI (≥ 10 m/s)	
		HR (95% CI)	p-value	HR (95% CI)	p-value	HR (95% CI)	p-value	HR (95% CI)	p-value	HR (95% CI)	p-value	HR (95% CI)	p-value
Metabolic healthy	Normal BMI	Ref		Ref		Ref		Ref		Ref		Ref	
	Overweight	1.25 (0.46 – 3.34)	0.664	1.22 (0.46 – 3.30)	0.685	1.22 (0.46 – 3.29)	0.686	1.06 (0.37 – 3.07)	0.912	0.89 (0.31 – 2.57)	0.827	0.89 (0.31 – 2.57)	0.827
	Obesity	2.07 (0.64 – 6.75)	0.227	1.94 (0.60 – 6.34)	0.271	1.95 (0.60 – 6.36)	0.269	1.34 (0.35 – 5.14)	0.669	0.91 (0.24 – 3.52)	0.895	0.93 (0.24 – 3.58)	0.916
Metabolic unhealthy	Normal BMI	2.52 (1.18 – 5.39)	0.017	2.45 (1.15 – 5.24)	0.020	2.45 (1.15 – 5.24)	0.020	2.43 (1.09 – 5.40)	0.029	2.20 (0.99 – 4.91)	0.054	2.23 (1.01 – 4.98)	0.049
	Overweight	3.28 (1.60 – 6.73)	0.001	3.11 (1.52 – 6.40)	0.002	3.11 (1.52 – 6.39)	0.002	2.59 (1.20 – 5.59)	0.016	2.11 (0.97 – 4.58)	0.061	2.15 (0.99 – 4.68)	0.054
	Obesity	5.15 (2.53 – 10.48)	< 0.001	4.61 (2.26 – 9.43)	< 0.001	4.62 (2.26 – 9.45)	< 0.001	3.46 (1.58 – 7.57)	0.002	2.24 (1.01 – 4.94)	0.046	2.29 (1.04 – 5.07)	0.040

†Main model was adjusted for age, smoking, ethnicity, Townsend deprivation quintiles.

Table S54 Sensitivity analysis of the relationship between BMI and metabolic health status on all-cause mortality among men, adjusting for lifestyle and biological factors.

Men											
Metabolic Health Status	BMI	Physical Activity		Alcohol Intake Frequency		Sleep Duration		hs-CRP		ASI (≥ 10 m/s)	
		HR (95% CI)	p-value	HR (95% CI)	p-value	HR (95% CI)	p-value	HR (95% CI)	p-value	HR (95% CI)	p-value
Metabolically healthy	Normal BMI	Ref		Ref		Ref		Ref		Ref	
	Overweight	0.93 (0.80 – 1.09)	0.379	0.94 (0.81 – 1.10)	0.465	0.94 (0.81 – 1.11)	0.472	0.91 (0.77 – 1.07)	0.251	0.90 (0.76 – 1.06)	0.221
	Obesity	1.41 (1.11 – 1.78)	0.005	1.43 (1.13 – 1.81)	0.003	1.42 (1.12 – 1.80)	0.004	1.28 (0.99 – 1.65)	0.059	1.27 (0.98 – 1.64)	0.066
Metabolically unhealthy	Normal BMI	1.33 (1.18 – 1.49)	< 0.001	1.33 (1.17 – 1.52)	< 0.001	1.33 (1.17 – 1.52)	< 0.001	1.31 (1.15 – 1.51)	< 0.001	1.31 (1.14 – 1.50)	< 0.001
	Overweight	1.21 (1.07 – 1.36)	0.002	1.21 (1.07 – 1.36)	0.002	1.21 (1.07 – 1.36)	0.001	1.15 (1.02 – 1.31)	0.025	1.14 (1.01 – 1.30)	0.037
	Obesity	1.60 (1.42 – 1.81)	< 0.001	1.60 (1.41 – 1.81)	< 0.001	1.61 (1.41 – 1.81)	< 0.001	1.44 (1.26 – 1.64)	< 0.001	1.42 (1.25 – 1.62)	< 0.001

†Main model was adjusted for age, smoking, ethnicity, Townsend deprivation quintiles.

Table S55 Sensitivity analysis of the relationship between BMI and metabolic health status on all-cause mortality among women, adjusting for lifestyle and biological factors.

Women											
**Metabolic Health Status**	**BMI**	**Physical Activity**		**Alcohol Intake Frequency**		**Sleep Duration**		**hs-CRP**		**ASI (≥ 10 m/s)**	
		**HR (95% CI)**	**p-value**	**HR (95% CI)**	**p-value**	**HR (95% CI)**	**p-value**	**HR (95% CI)**	**p-value**	**HR (95% CI)**	**p-value**
**Metabolically healthy**	**Normal BMI**	Ref		Ref		Ref		Ref		Ref	
	**Overweight**	1.07 (0.92 – 1.24)	0.374	1.06 (0.92 – 1.23)	0.374	1.06 (0.92 – 1.23)	0.426	1.02 (0.87 – 1.19)	0.850	1.01 (0.87 – 1.19)	0.858
	**Obesity**	1.23 (0.99 – 1.52)	0.058	1.19 (0.96 – 1.47)	0.115	1.19 (0.96 – 1.47)	0.116	0.94 (0.74 – 1.20)	0.640	0.94 (0.74 – 1.20)	0.625
**Metabolically unhealthy**	**Normal BMI**	1.33 (1.18 – 1.50)	< 0.001	1.32 (1.17 – 1.48)	< 0.001	1.31 (1.17 – 1.48)	< 0.001	1.30 (1.15 – 1.47)	< 0.001	1.30 (1.14 – 1.47)	< 0.001
	**Overweight**	1.26 (1.12 – 1.41)	< 0.001	1.23 (1.09 – 1.38)	< 0.001	1.22 (1.09 – 1.37)	< 0.001	1.15 (1.02 – 1.30)	0.025	1.15 (1.01 – 1.30)	0.029
	**Obesity**	1.61 (1.43 – 1.81)	< 0.001	1.52 (1.35 – 1.71)	< 0.001	1.52 (1.35 – 1.71)	< 0.001	1.32 (1.16 – 1.50)	< 0.001	1.31 (1.15 – 1.50)	< 0.001

†Main model was adjusted for age, smoking, ethnicity, Townsend deprivation quintiles.

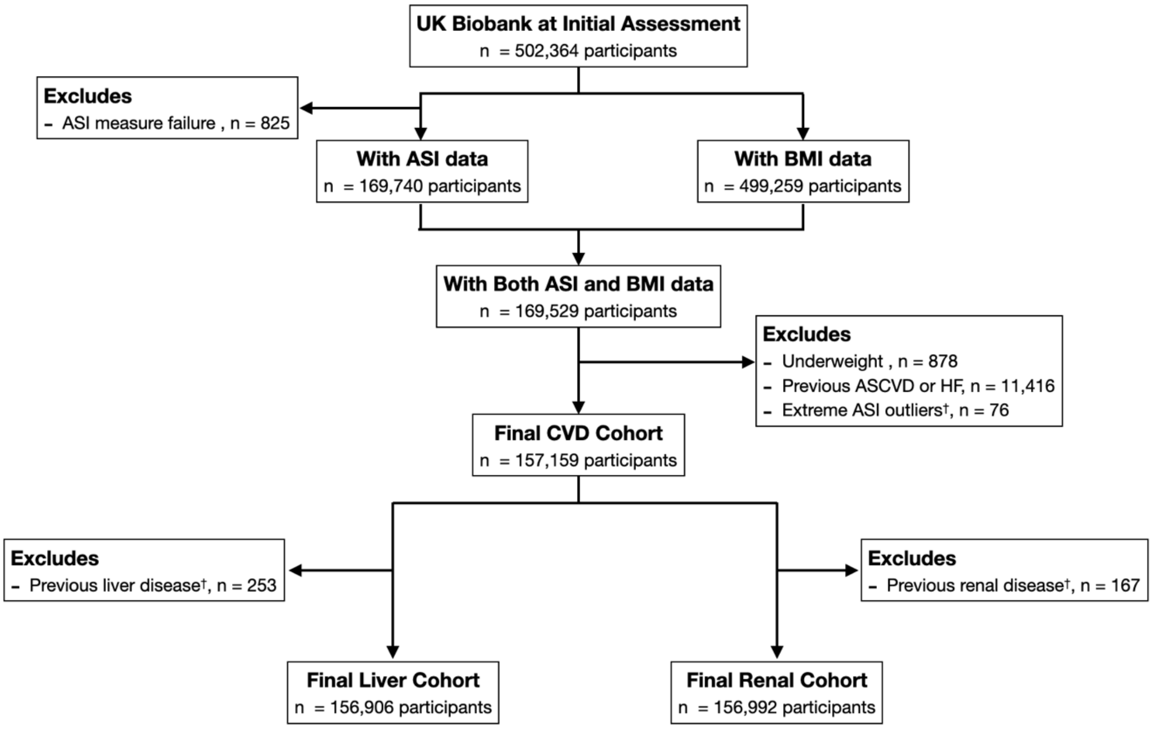

Figure S1: Flowchart of participant selection for analysis

† Outliers were defined as mean +/- 5 standard deviations (ASI ≥ 29.59 m/s).

Figure S2 Relationship between BMI and the risk of CHD, ischaemic stroke, PAD, MI, and CV death, in men and women by metabolic health status.

¥ Metabolically unhealthy defined as presence any of hypertension, diabetes or dyslipidaemia.

¥ Analyses adjusted for age, smoking status, ethnicity, and Townsend deprivation quintiles.

<table>
<tr><th></th><th>Men</th><th>Women</th></tr>
<tr><td>Coronary Heart Disease (CHD)</td><td>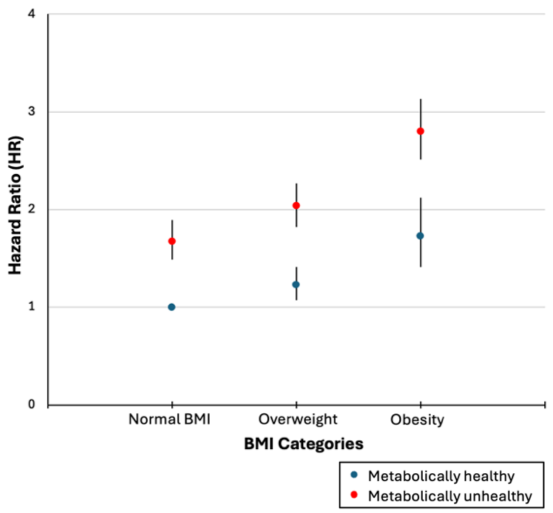
</td><td>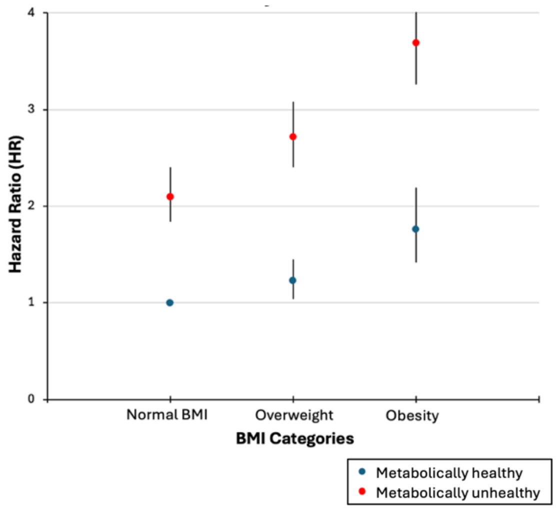
</td></tr>
<tr><td>Ischaemic Stroke (IS)</td><td>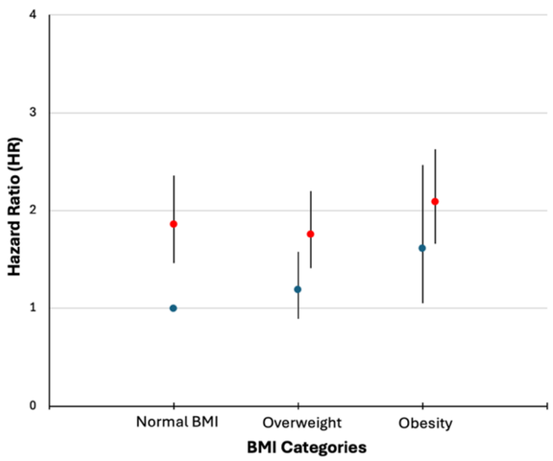
</td><td>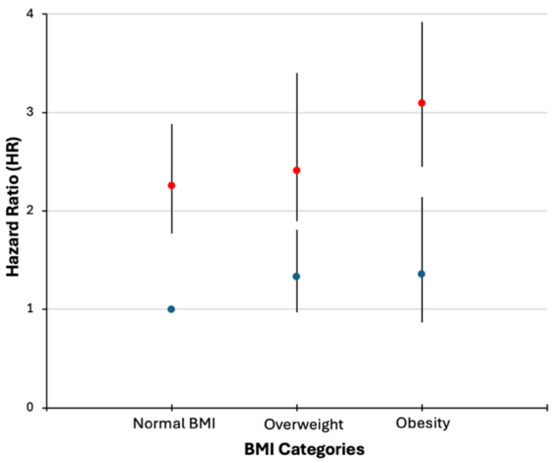
</td></tr>
<tr><td>Peripheral Artery Disease (PAD)</td><td>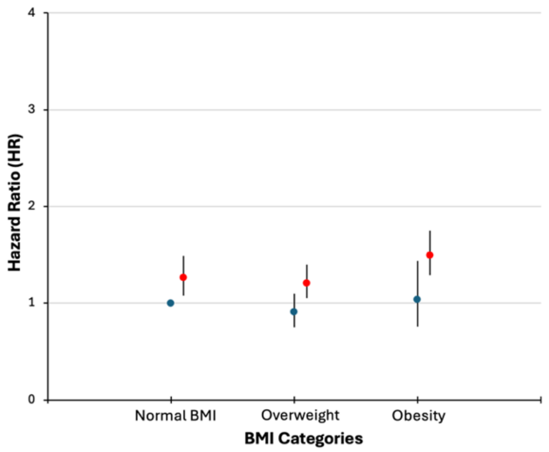
</td><td>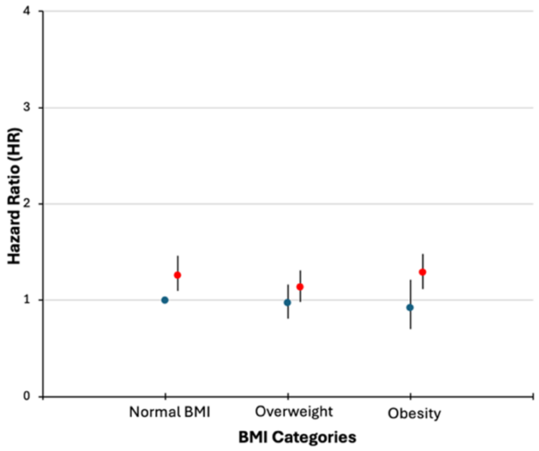
</td></tr>
</table>

<table>
<tr>
<td>Myocardial Infarction (MI)</td>
<td>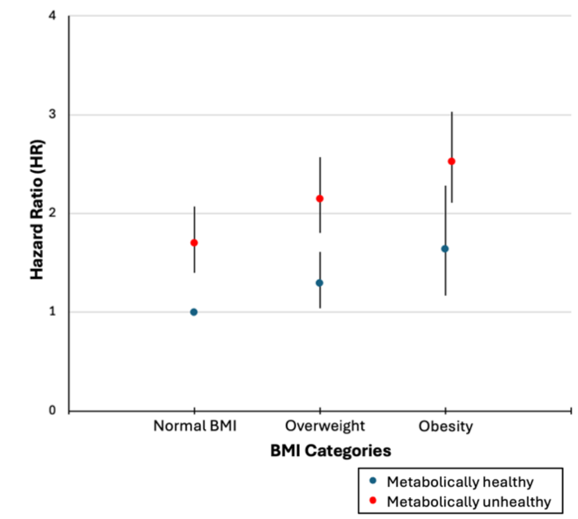
</td>
<td>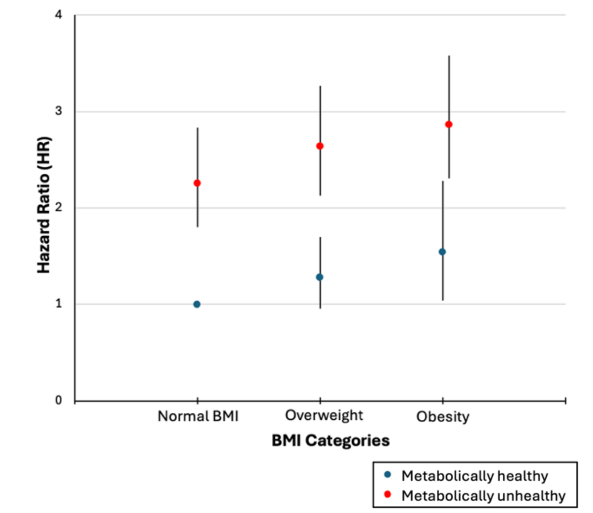
</td>
</tr>
<tr>
<td>Cardiovascular Death</td>
<td>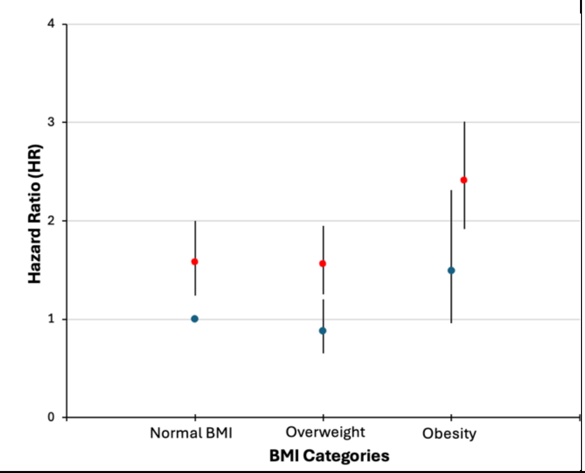
</td>
<td>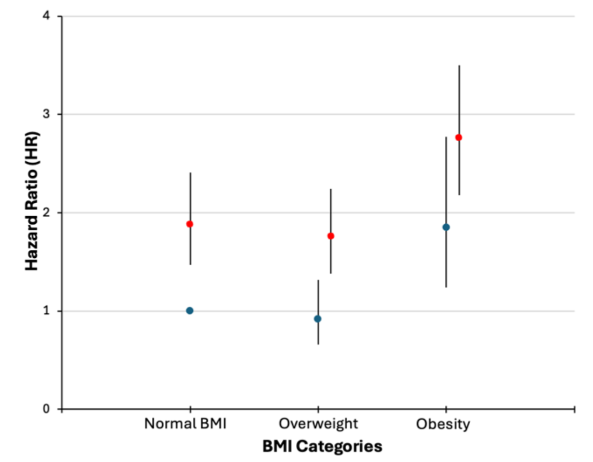
</td>
</tr>
</table>

Figure S3 Relationship between BMI and the risk of strokes (all) and haemorrhagic strokes, in men and women by metabolic health status.

¥ Metabolically unhealthy defined as presence any of hypertension, diabetes or dyslipidaemia.

¥ Analyses adjusted for age, smoking status, ethnicity, and Townsend deprivation quintiles.

	Men	Women
**Strokes (All)**	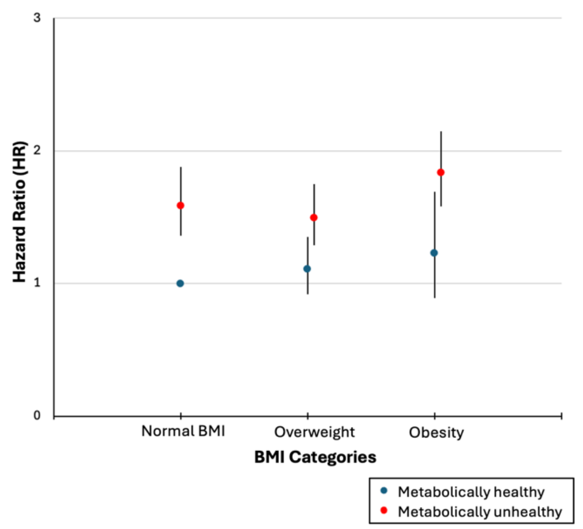	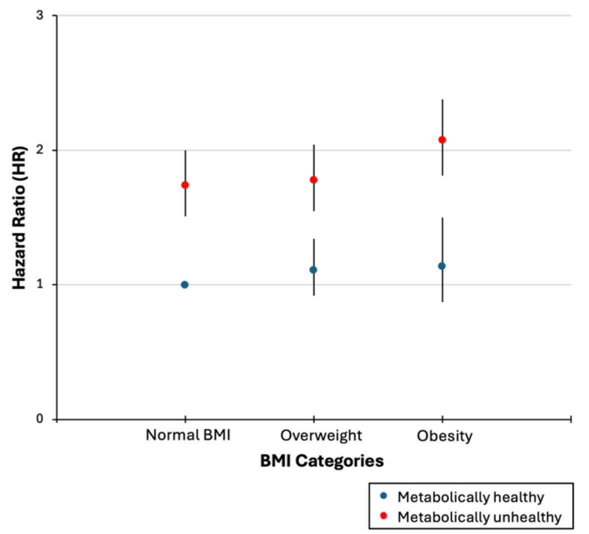
**Haemorrhagic Strokes**	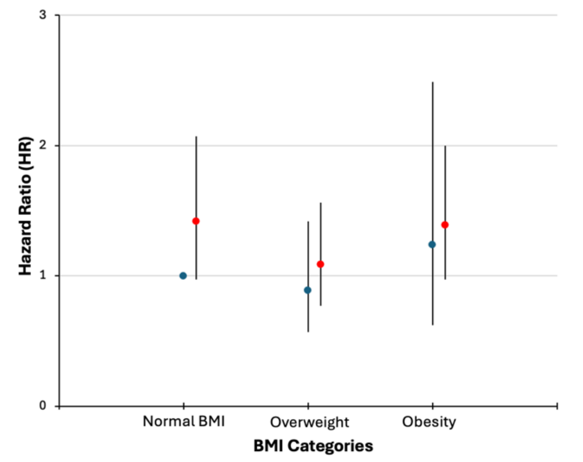	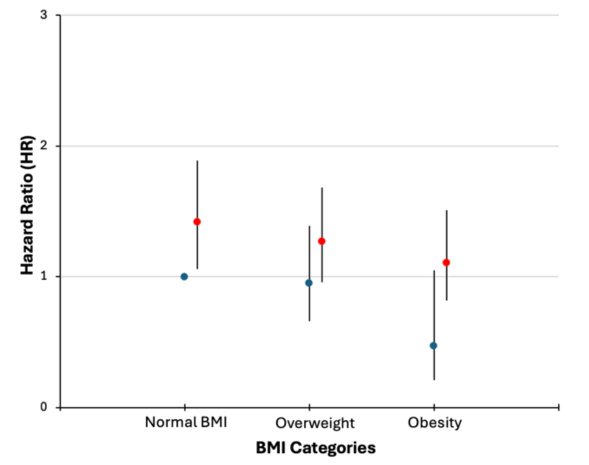

Figure S4 Relationship between severity of obesity and metabolic health status on the risk of CHD, ischaemic stroke, PAD, MI, and CV death, in men and women.
¥ Metabolically unhealthy defined as presence any of hypertension, diabetes or dyslipidaemia.
¥ Analyses adjusted for age, smoking status, ethnicity, and Townsend deprivation quintiles.

	Men	Women

Coronary Heart Disease (CHD)

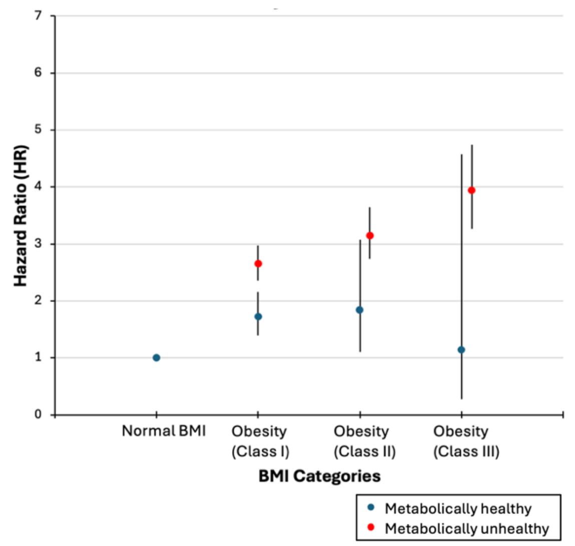

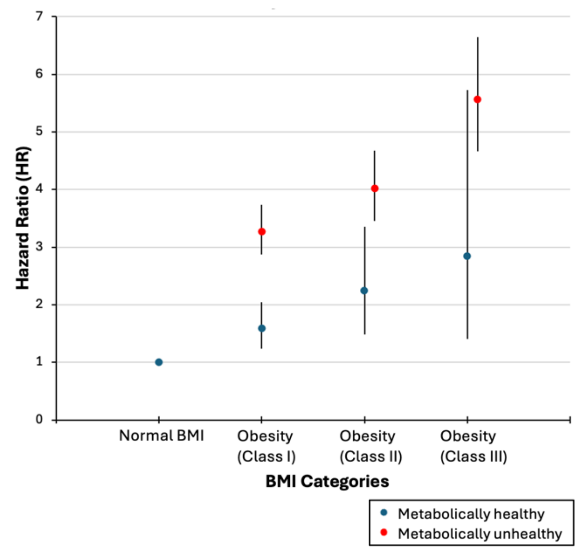

Ischaemic Stroke

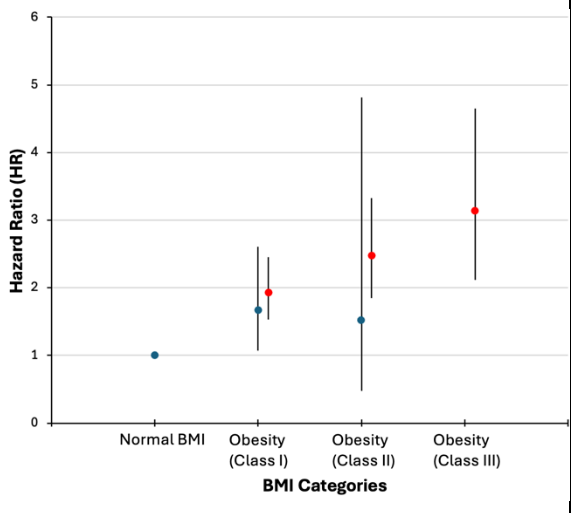

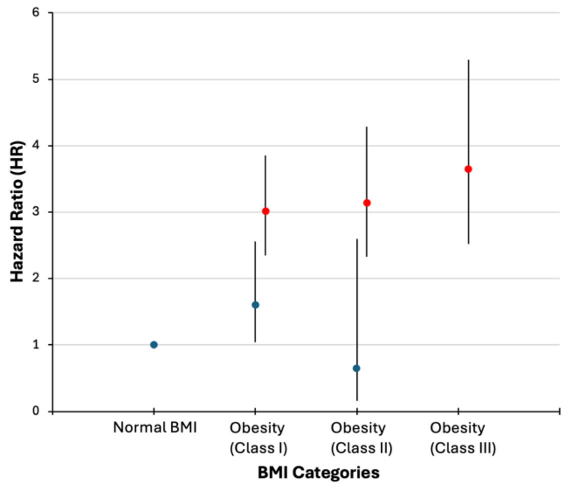

Peripheral Artery Disease (PAD)

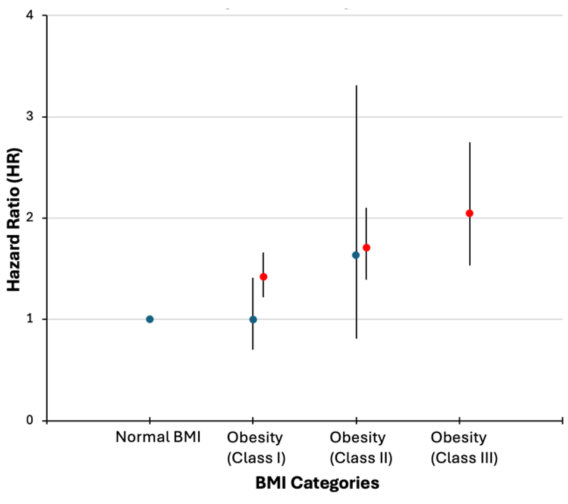

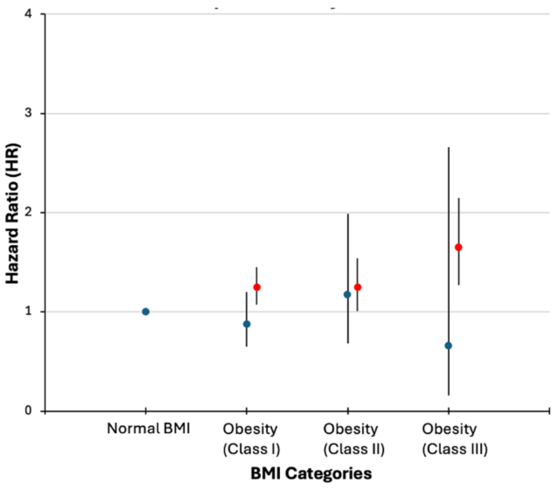

Myocardial Infarction (MI)

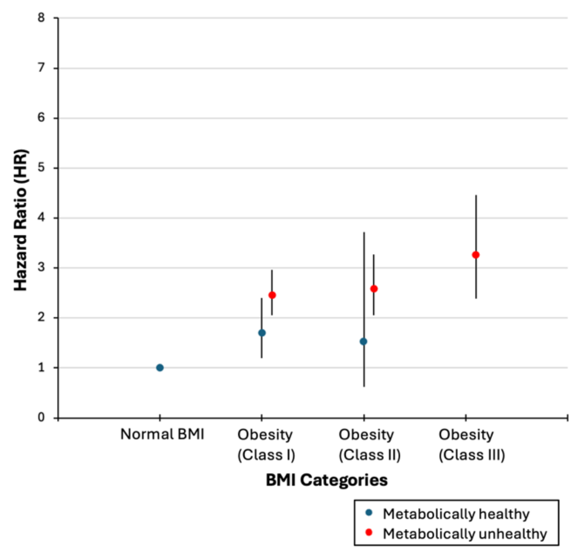

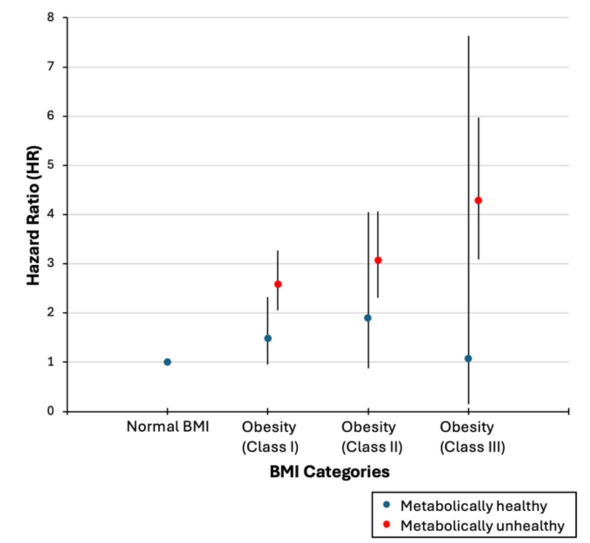

Cardiovascular Death

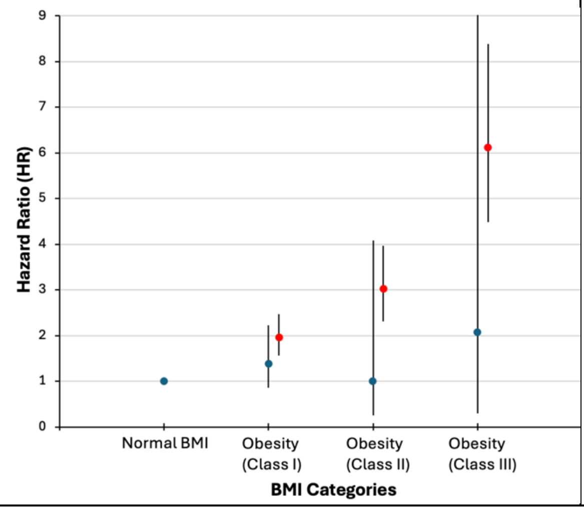

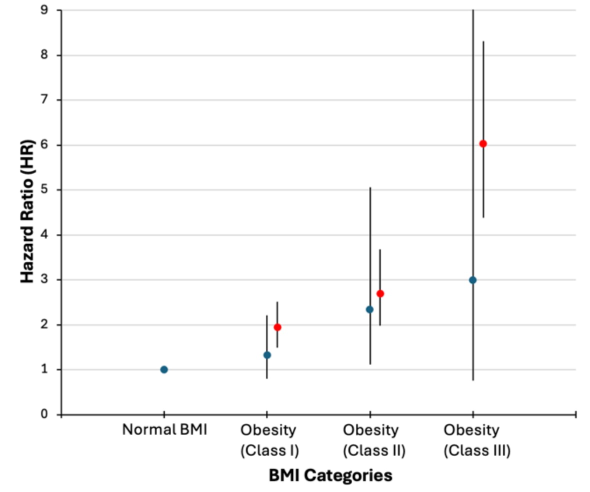

Figure S5 Relationship between the presence of central obesity within BMI categories and metabolic health status on the risk of CHD, MI, ischaemic stroke, and CV death, in men and women.

¥ Metabolically unhealthy defined as presence any of hypertension, diabetes or dyslipidaemia.

¥ Analyses adjusted for age, smoking status, ethnicity, and Townsend deprivation quintiles.

<table>
<tr><th></th><th>Men</th><th>Women</th></tr>
<tr><td>Coronary Heart Disease (CHD)</td><td>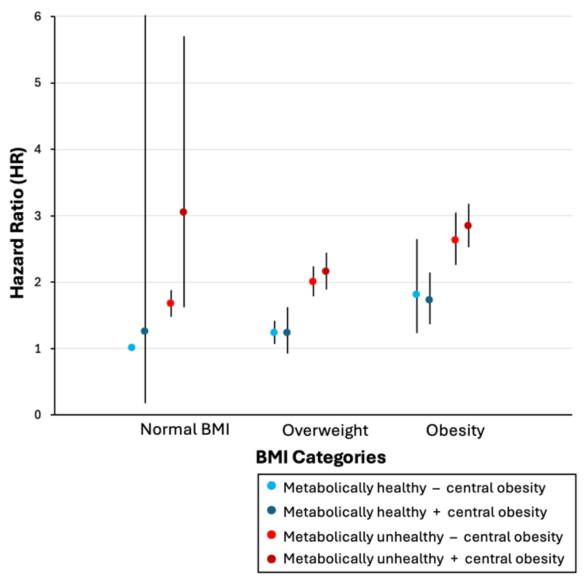
</td><td>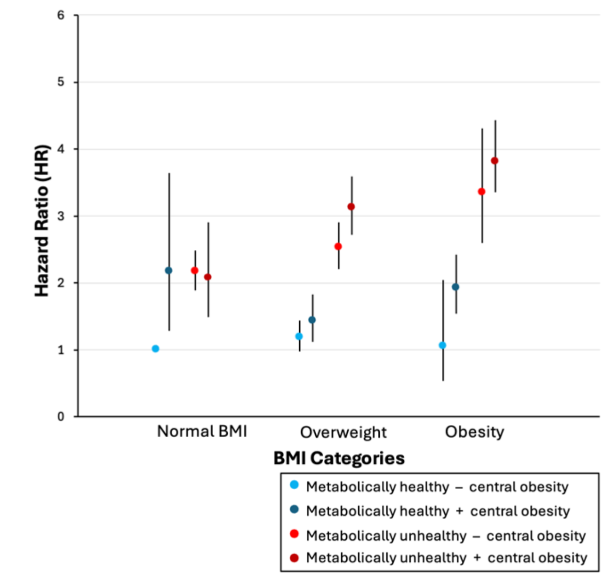
</td></tr>
<tr><td>Myocardial Infarction (MI)</td><td>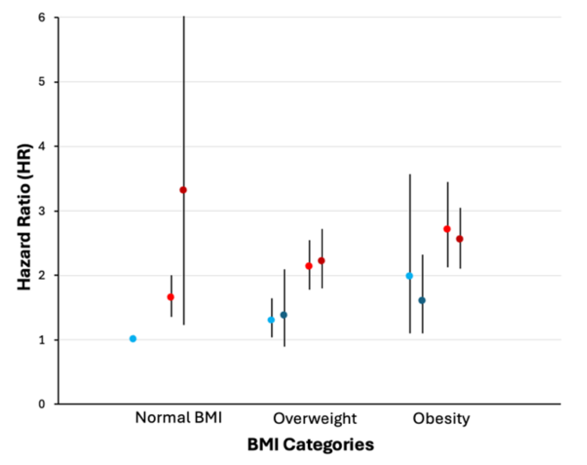
</td><td>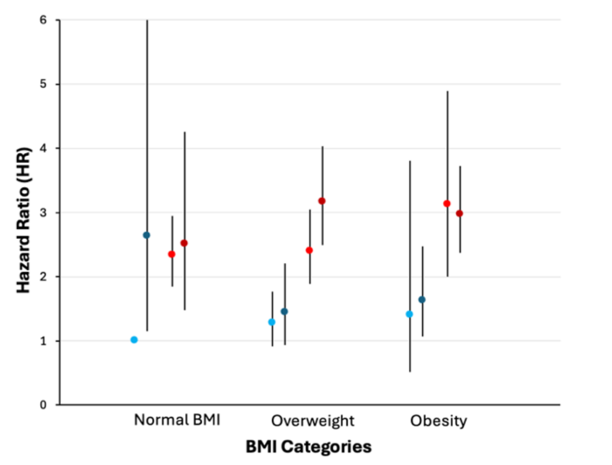
</td></tr>
<tr><td>Ischaemic Stroke (IS)</td><td>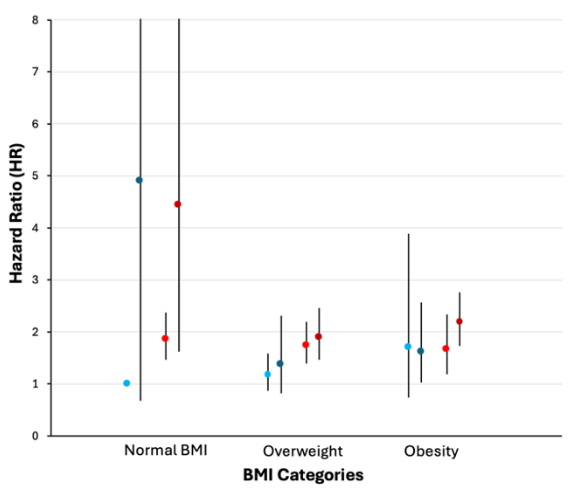
</td><td>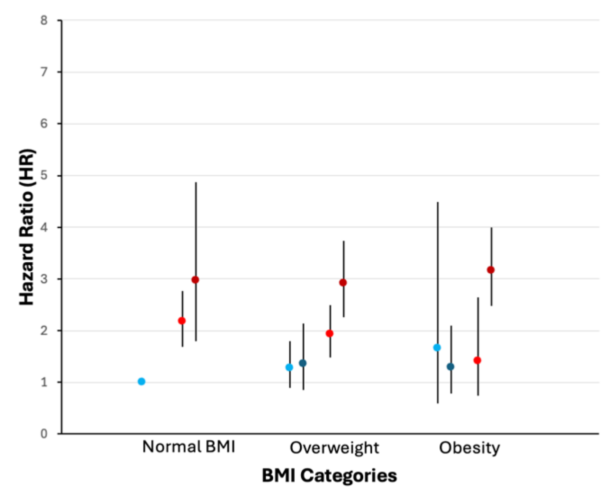
</td></tr>
</table>

Cardiovascular Death

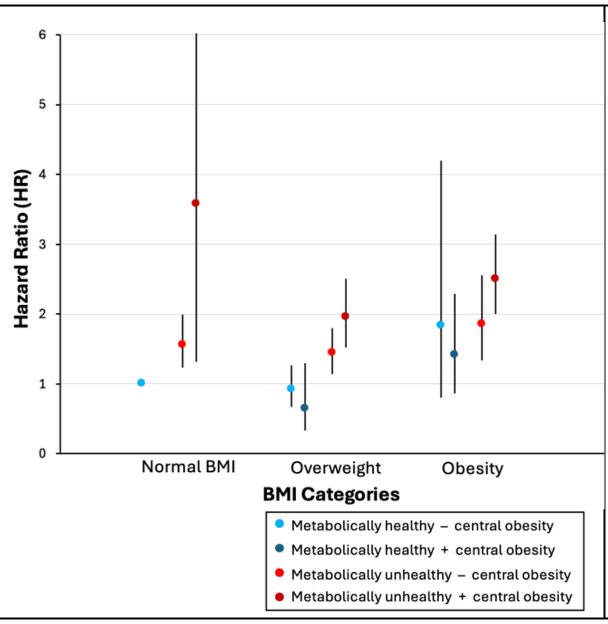

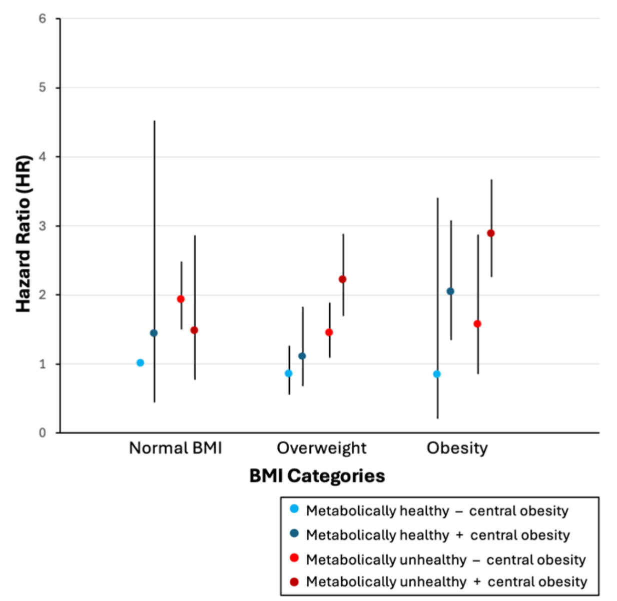

Figure S6 Relationship between the number of metabolic abnormalities present within different categories of BMI and the risk of CHD, ischaemic stroke, PAD, MI, and CV death, in men and women.
¥ Analyses adjusted for age, smoking status, ethnicity, and Townsend deprivation quintiles.

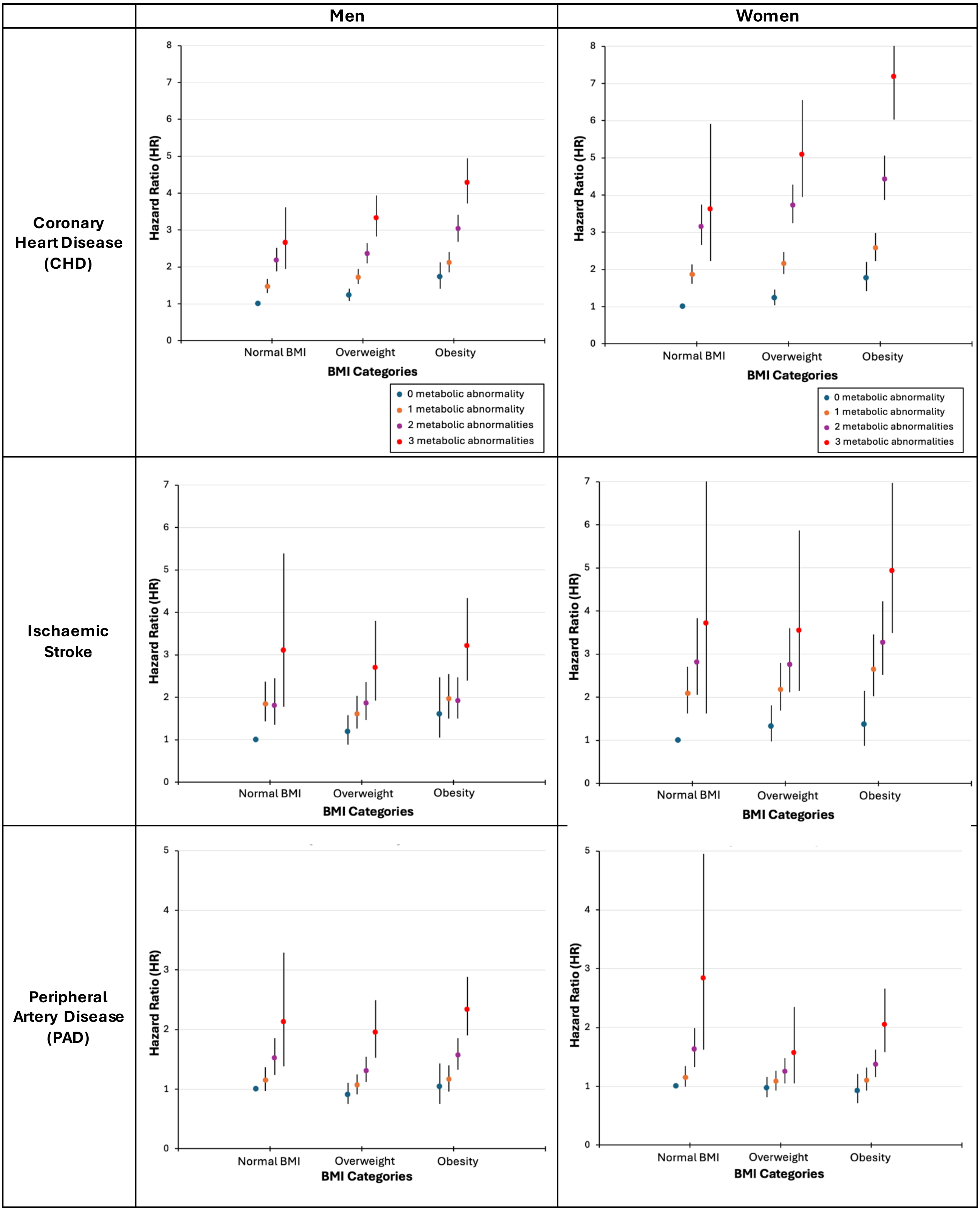

Myocardial Infarction (MI)

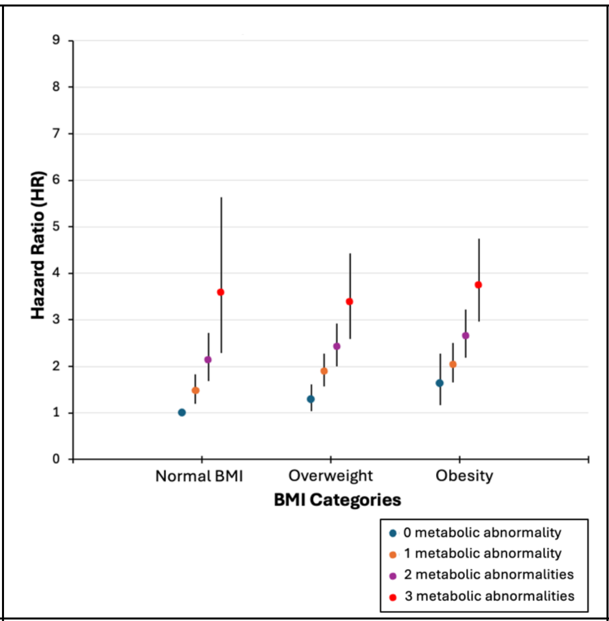

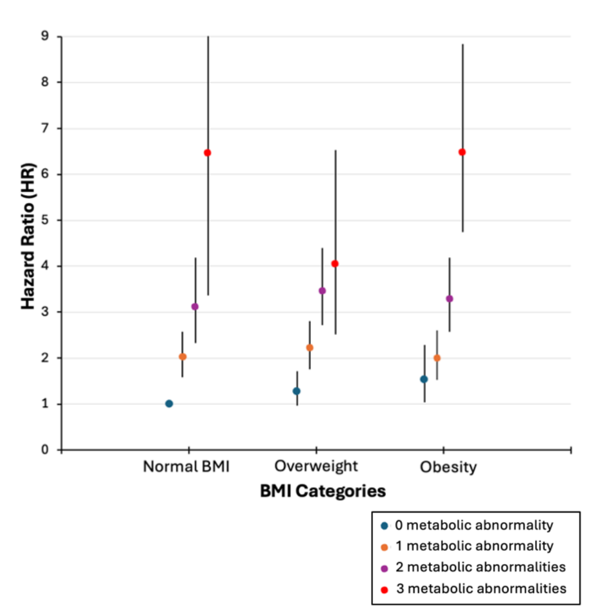

Cardiovascular Death

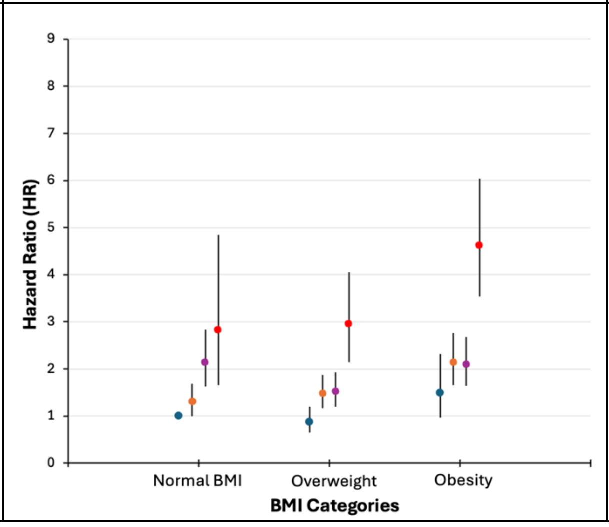

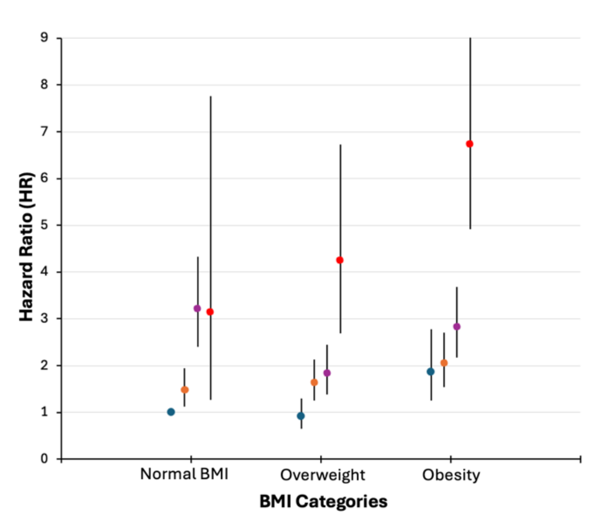

Figure S7 Sensitivity analysis of the relationship between BMI and metabolic health after adjusting for biomarkers of metabolic health profile in men and women with the risk of cardiovascular, hepatic, renal and mortality outcomes.

¥ Analyses adjusted for age, smoking status, ethnicity, Townsend deprivation quintiles, systolic blood pressure, HbA1C, history of diabetes, HDL, LDL, and triglycerides levels.

	Men	Women
**Atherosclerotic Cardiovascular Disease (ASCVD)**	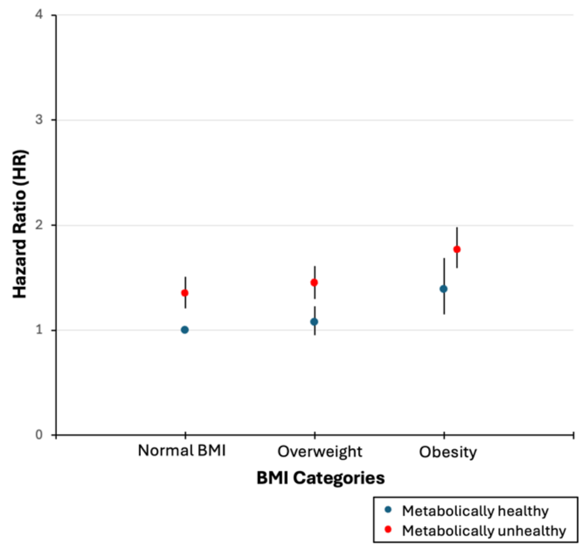	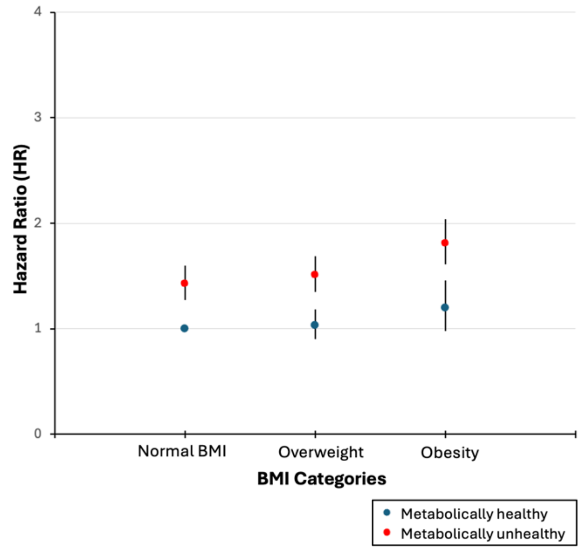
**Coronary Heart Disease (CHD)**	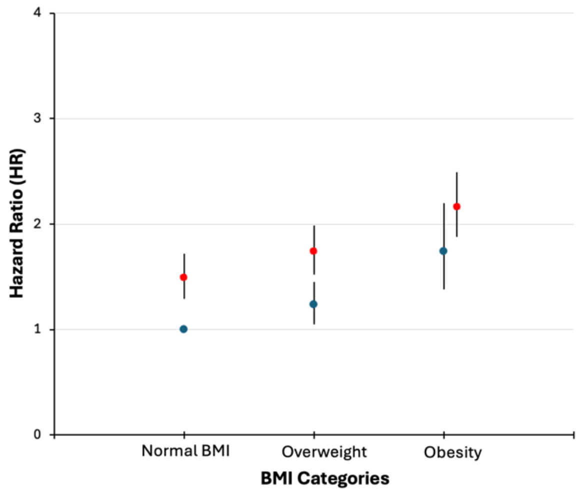	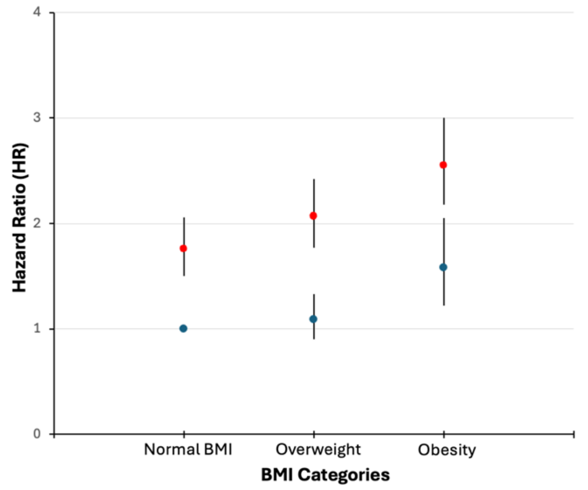
**Ischaemic Stroke (IS)**	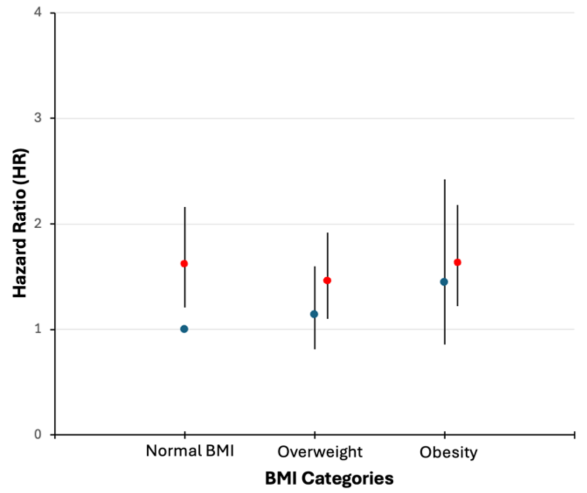	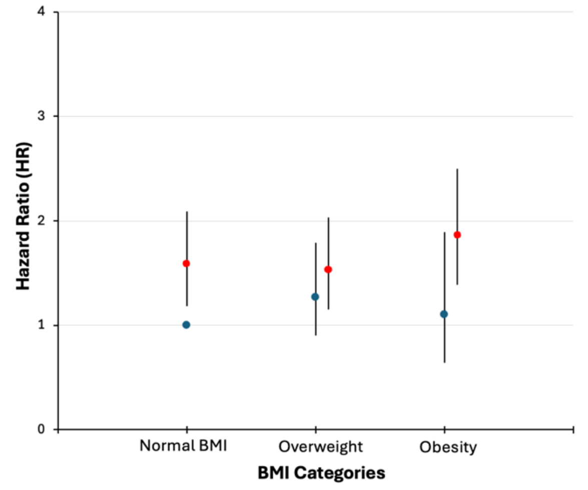

Heart Failure (HF)

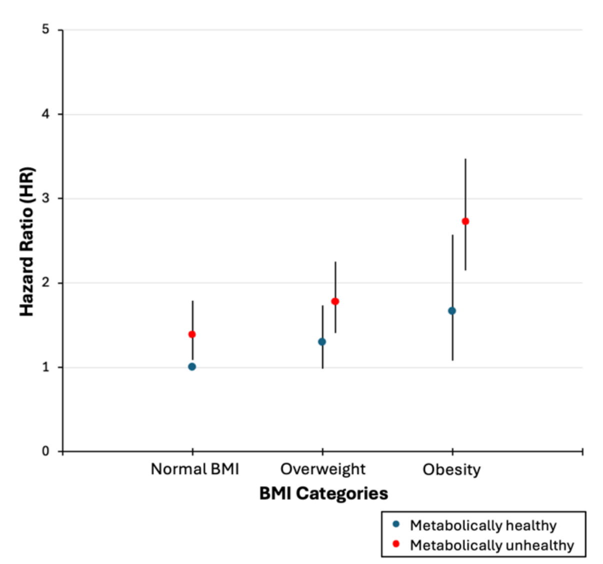

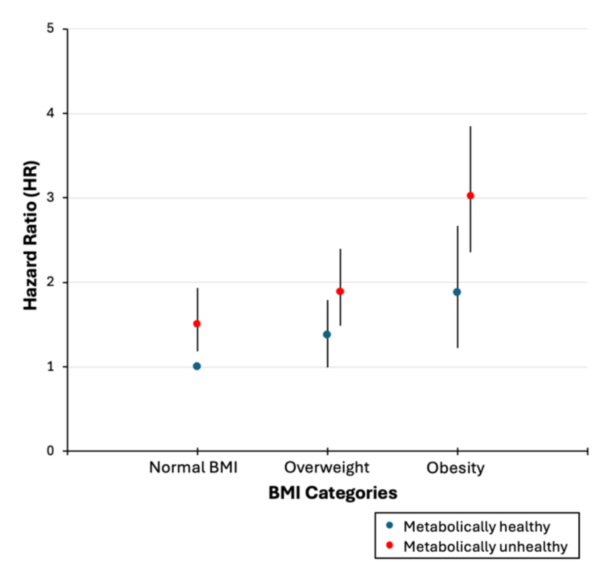

Cardiovascular Death

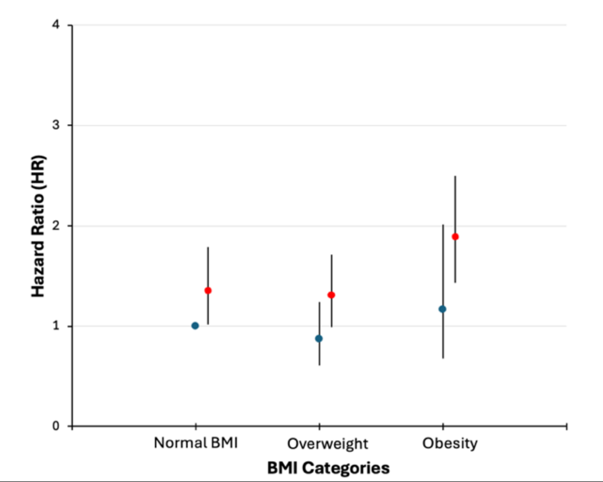

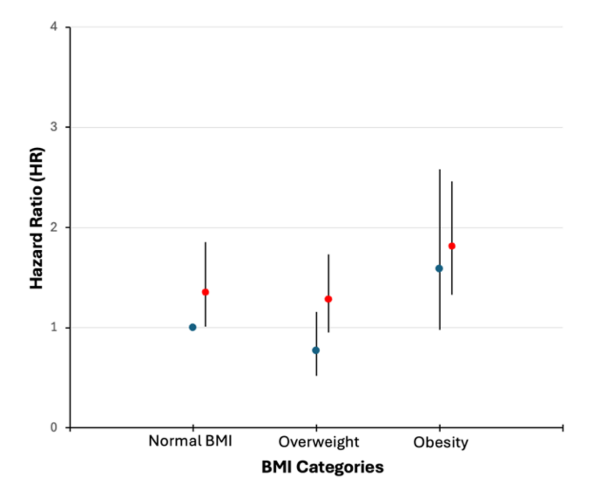

Metabolic Dysfunction Associated Steatotic Liver Disease (MASLD)

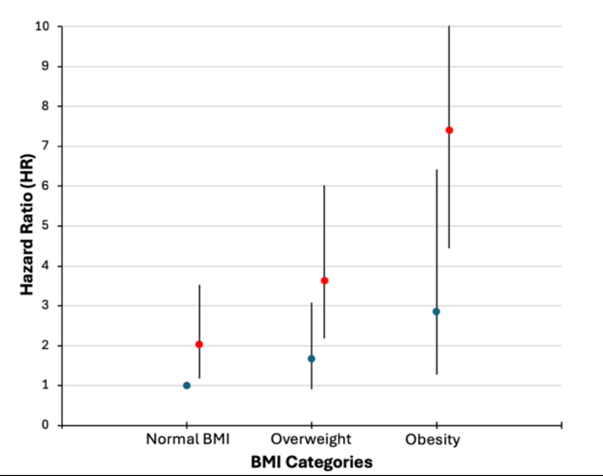

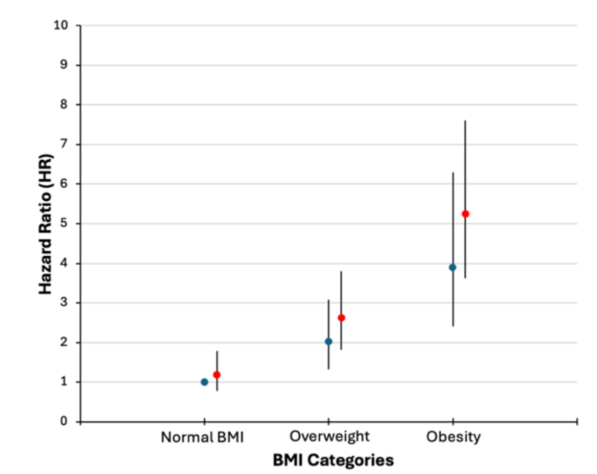

End-Stage Renal Disease (ESRD)

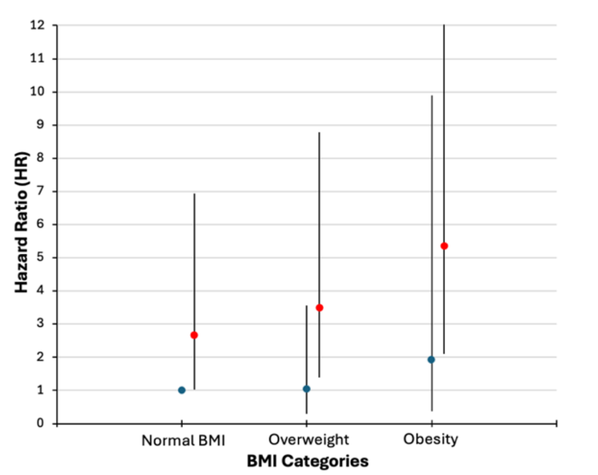

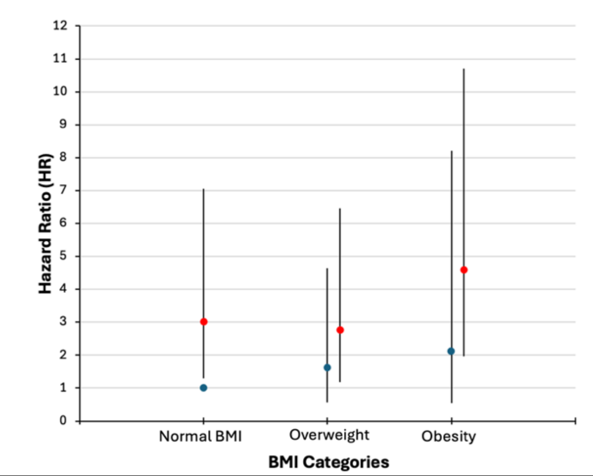

All-Cause Mortality

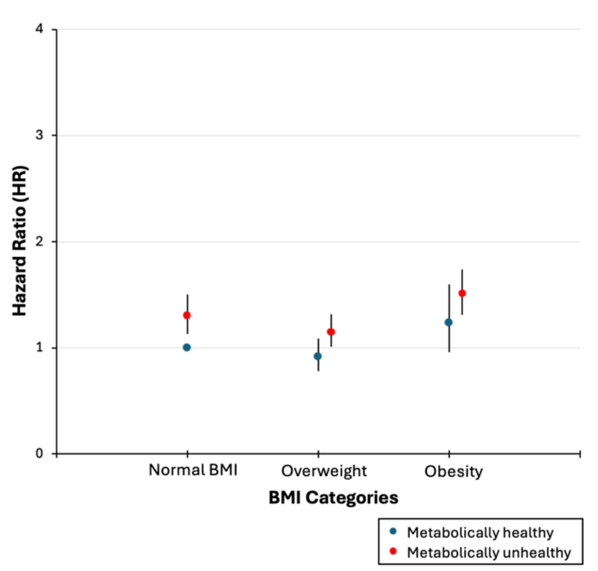

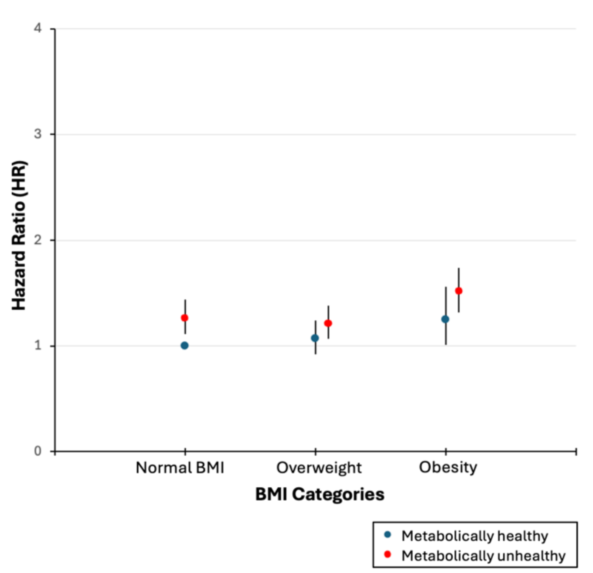

Figure S8: Sensitivity analysis of the relationship between obesity and metabolic health status after adjusting for lifestyle and biological factors in stepwise manner in Men with the risk of atherosclerotic cardiovascular disease **(A)**, coronary heart disease **(B)**, ischaemic stroke **(C)**, peripheral artery disease **(D)**, myocardial infarction **(E)**, heart failure **(F)**, metabolic dysfunction-associated steatotic liver disease **(G)**, end stage renal disease **(H)**, cardiovascular death **(I)** and all-cause mortality **(J)**.

¥ Main model adjusted for age, smoking status, ethnicity, and Townsend deprivation quintiles.
¥ Reference group was metabolically healthy participants with normal BMI.
¥ The cox-regression model generated above includes all six main metabolic categories. Only two metabolic categories representing obesity were included here to allow comparison.

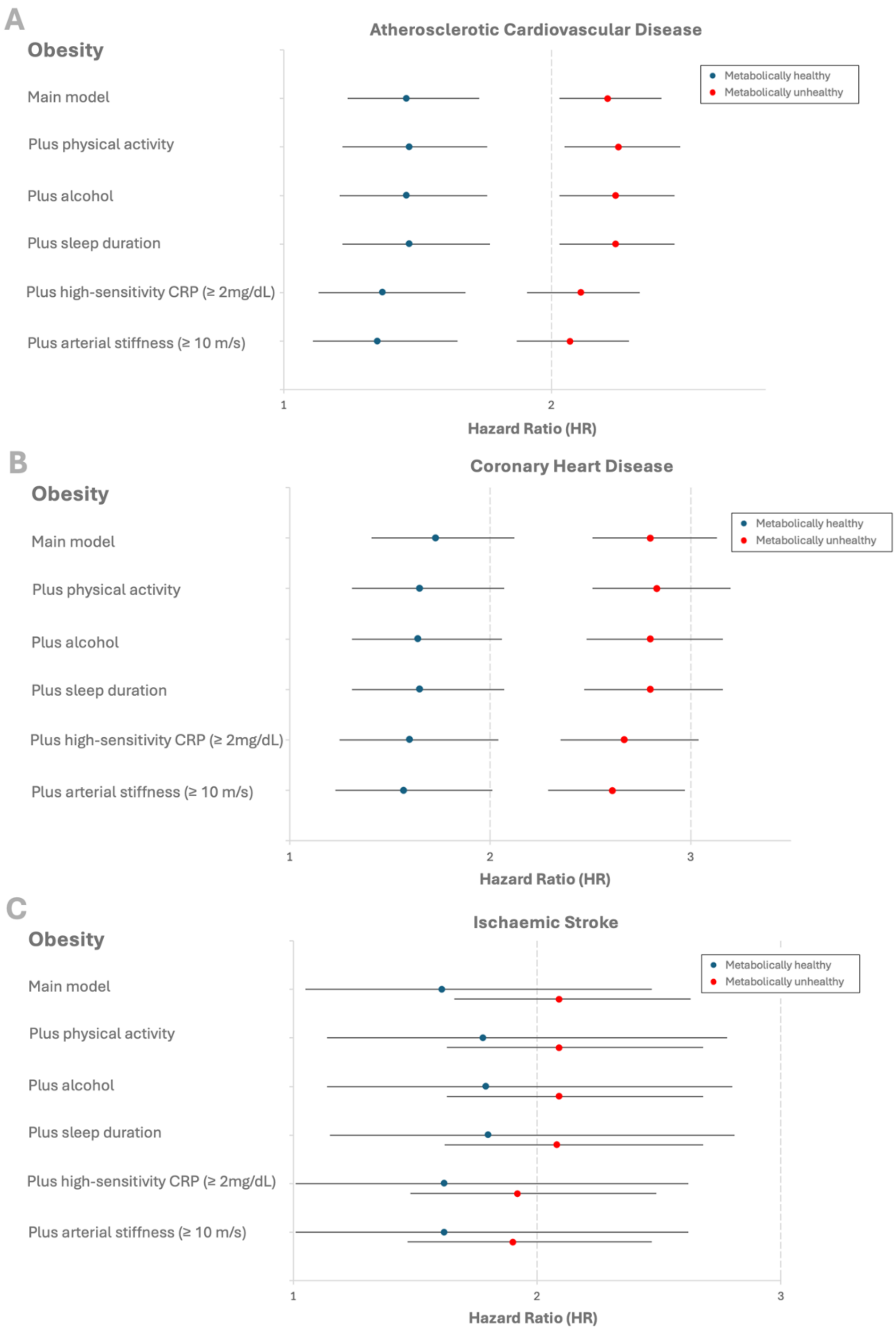

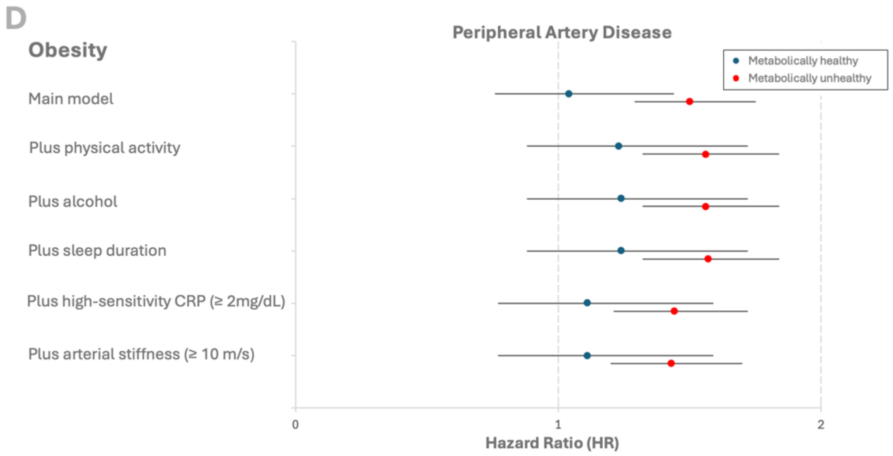
D
Obesity
Peripheral Artery Disease
Metabolically healthy
Metabolically unhealthy
Main model
Plus physical activity
Plus alcohol
Plus sleep duration
Plus high-sensitivity CRP (≥ 2mg/dL)
Plus arterial stiffness (≥ 10 m/s)
0
1
2
Hazard Ratio (HR)

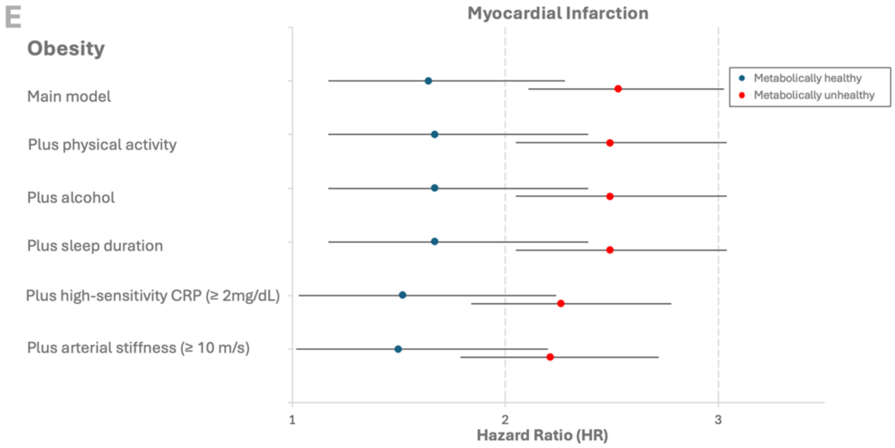
E
Myocardial Infarction
Obesity
Metabolically healthy
Metabolically unhealthy
Main model
Plus physical activity
Plus alcohol
Plus sleep duration
Plus high-sensitivity CRP (≥ 2mg/dL)
Plus arterial stiffness (≥ 10 m/s)
1
2
3
Hazard Ratio (HR)

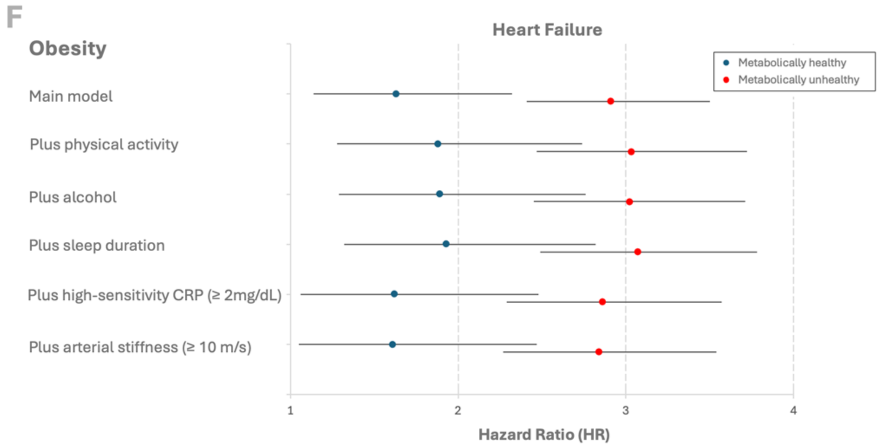
F
Heart Failure
Obesity
Metabolically healthy
Metabolically unhealthy
Main model
Plus physical activity
Plus alcohol
Plus sleep duration
Plus high-sensitivity CRP (≥ 2mg/dL)
Plus arterial stiffness (≥ 10 m/s)
1
2
3
4
Hazard Ratio (HR)

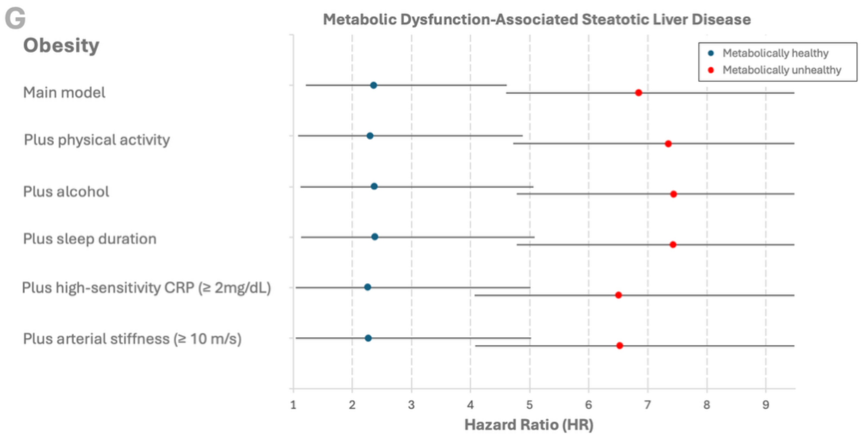
G
Metabolic Dysfunction-Associated Steatotic Liver Disease
Obesity
Metabolically healthy
Metabolically unhealthy
Main model
Plus physical activity
Plus alcohol
Plus sleep duration
Plus high-sensitivity CRP (≥ 2mg/dL)
Plus arterial stiffness (≥ 10 m/s)
1
2
3
4
5
6
7
8
9
Hazard Ratio (HR)

H

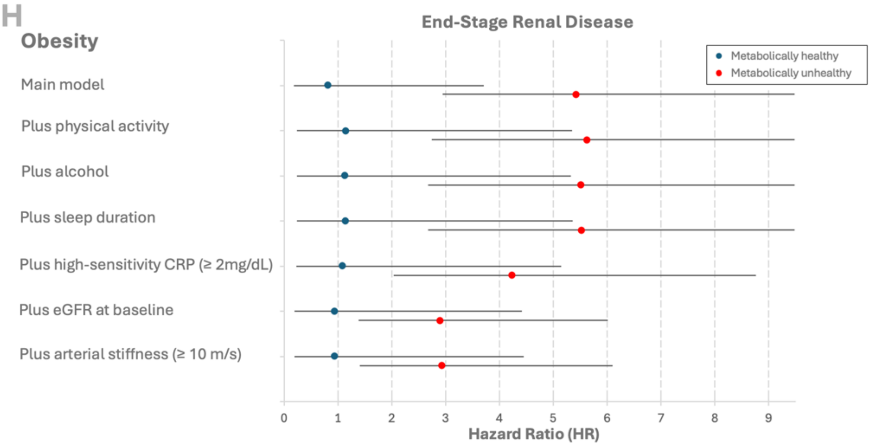
End-Stage Renal Disease
Obesity
Main model
Plus physical activity
Plus alcohol
Plus sleep duration
Plus high-sensitivity CRP (≥ 2mg/dL)
Plus eGFR at baseline
Plus arterial stiffness (≥ 10 m/s)
Metabolically healthy
Metabolically unhealthy
0
1
2
3
4
5
6
7
8
9
Hazard Ratio (HR)

I

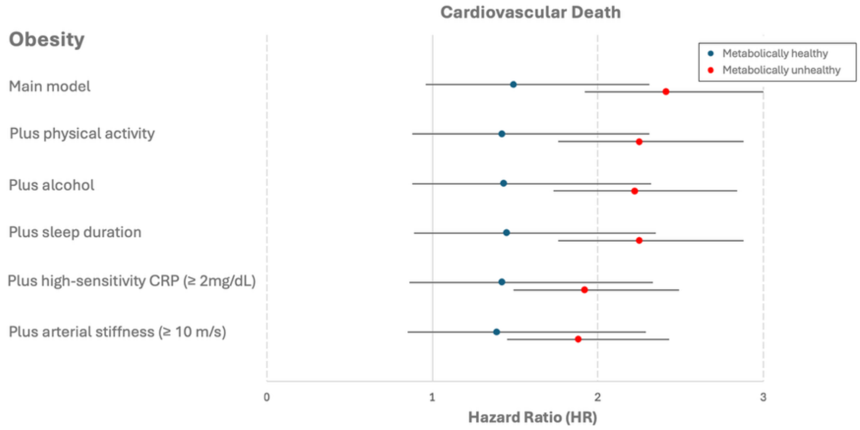
Cardiovascular Death
Obesity
Main model
Plus physical activity
Plus alcohol
Plus sleep duration
Plus high-sensitivity CRP (≥ 2mg/dL)
Plus arterial stiffness (≥ 10 m/s)
Metabolically healthy
Metabolically unhealthy
0
1
2
3
Hazard Ratio (HR)

J

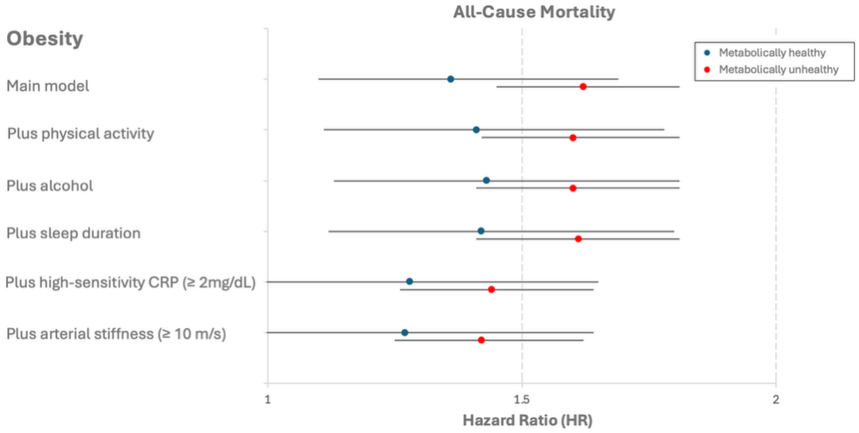
All-Cause Mortality
Obesity
Main model
Plus physical activity
Plus alcohol
Plus sleep duration
Plus high-sensitivity CRP (≥ 2mg/dL)
Plus arterial stiffness (≥ 10 m/s)
Metabolically healthy
Metabolically unhealthy
1
1.5
2
Hazard Ratio (HR)

Figure S9: Sensitivity analysis of the relationship between obesity and metabolic health status after adjusting for lifestyle and biological factors in stepwise manner in Women with the risk of atherosclerotic cardiovascular disease **(A)**, coronary heart disease **(B)**, ischaemic stroke **(C)**, peripheral artery disease **(D)**, myocardial infarction **(E)**, heart failure **(F)**, metabolic dysfunction-associated steatotic liver disease **(G)**, end stage renal disease **(H)**, cardiovascular death **(I)** and all-cause mortality **(J)**.

¥ Main model adjusted for age, smoking status, ethnicity, and Townsend deprivation quintiles.
¥ Reference group was metabolically healthy participants with normal BMI.
¥ The cox-regression model generated above includes all six main metabolic categories. Only two metabolic categories representing obesity were included here to allow comparison.

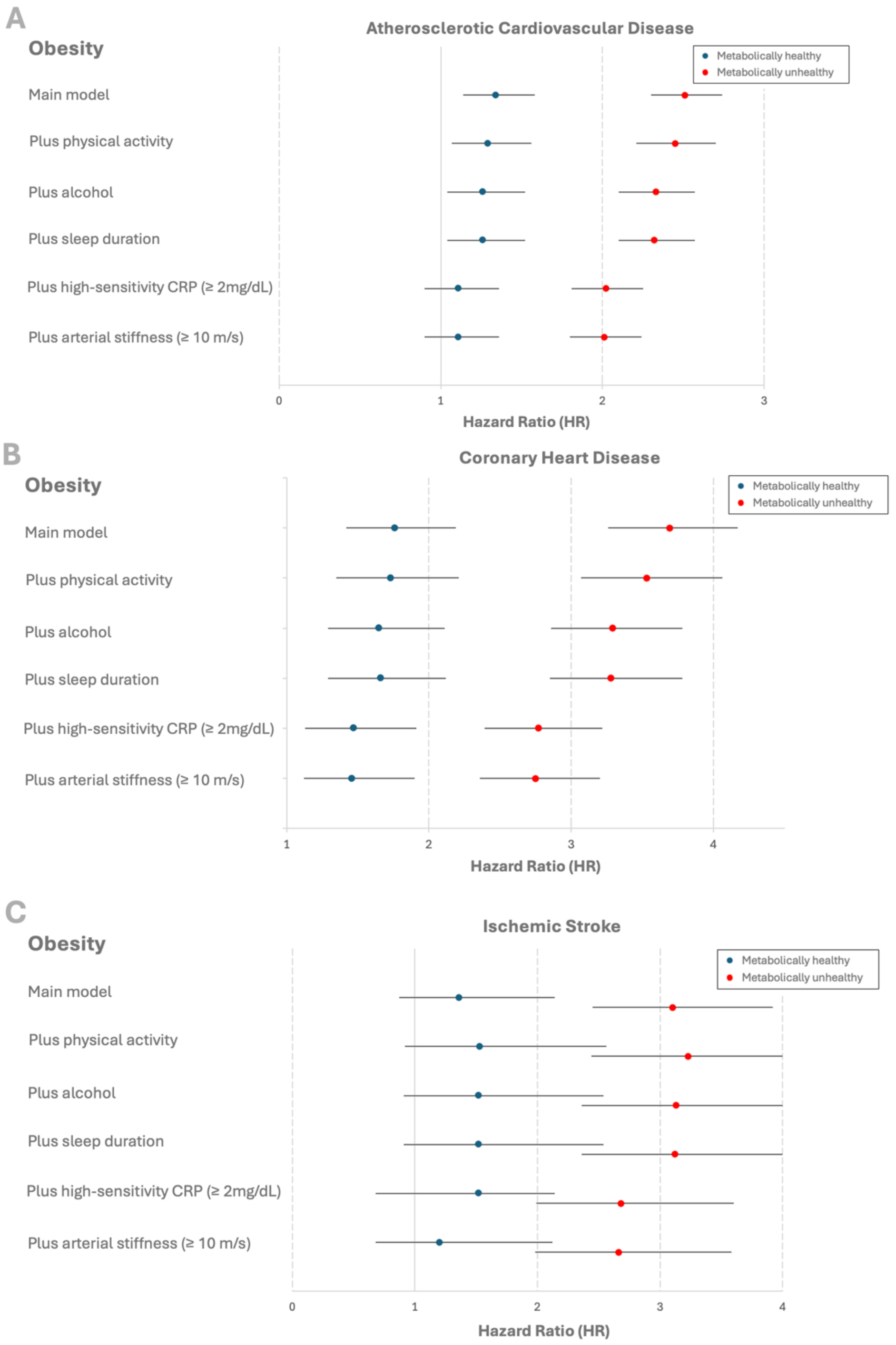

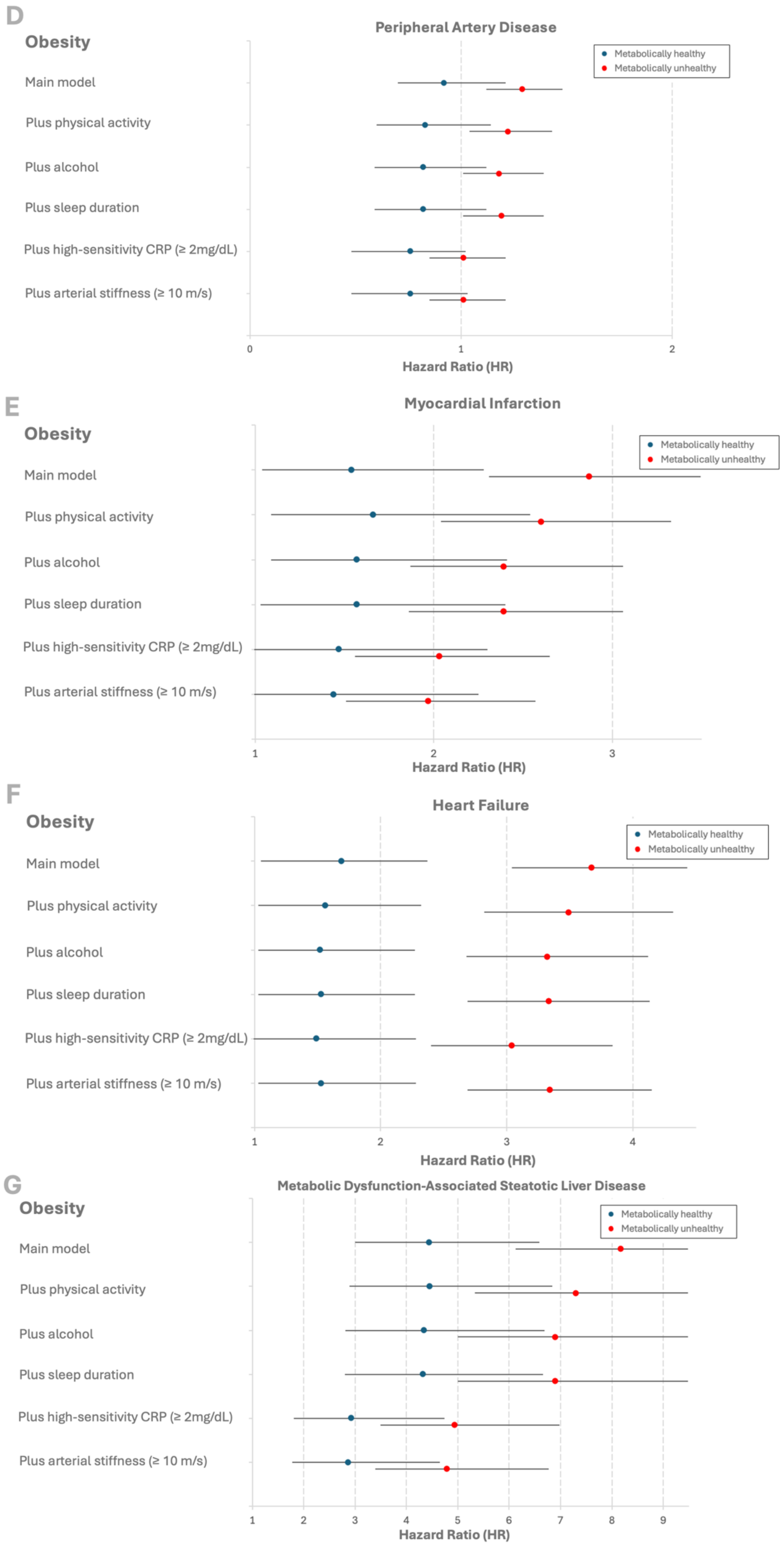
D
Obesity
Peripheral Artery Disease
Metabolically healthy
Metabolically unhealthy
Main model
Plus physical activity
Plus alcohol
Plus sleep duration
Plus high-sensitivity CRP (≥ 2mg/dL)
Plus arterial stiffness (≥ 10 m/s)
0
1
2
Hazard Ratio (HR)
E
Obesity
Myocardial Infarction
Metabolically healthy
Metabolically unhealthy
Main model
Plus physical activity
Plus alcohol
Plus sleep duration
Plus high-sensitivity CRP (≥ 2mg/dL)
Plus arterial stiffness (≥ 10 m/s)
1
2
3
Hazard Ratio (HR)
F
Obesity
Heart Failure
Metabolically healthy
Metabolically unhealthy
Main model
Plus physical activity
Plus alcohol
Plus sleep duration
Plus high-sensitivity CRP (≥ 2mg/dL)
Plus arterial stiffness (≥ 10 m/s)
1
2
3
4
Hazard Ratio (HR)
G
Obesity
Metabolic Dysfunction-Associated Steatotic Liver Disease
Metabolically healthy
Metabolically unhealthy
Main model
Plus physical activity
Plus alcohol
Plus sleep duration
Plus high-sensitivity CRP (≥ 2mg/dL)
Plus arterial stiffness (≥ 10 m/s)
1
2
3
4
5
6
7
8
9
Hazard Ratio (HR)

H

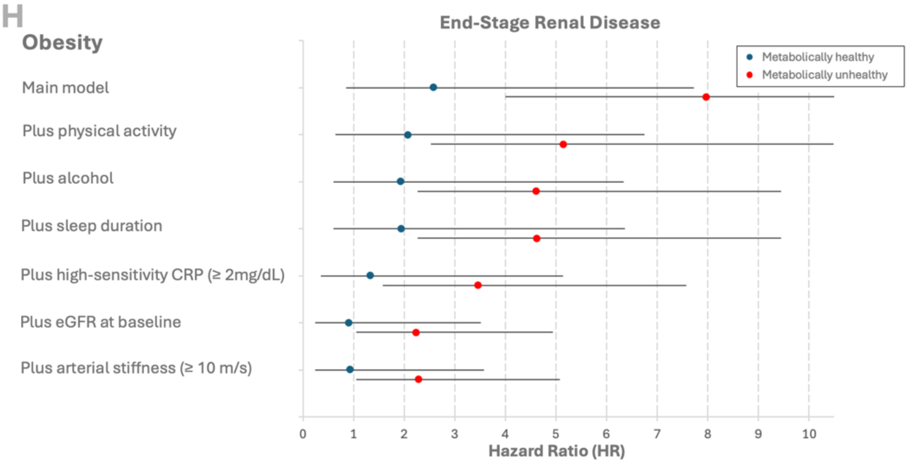
End-Stage Renal Disease
Obesity
Main model
Plus physical activity
Plus alcohol
Plus sleep duration
Plus high-sensitivity CRP (≥ 2mg/dL)
Plus eGFR at baseline
Plus arterial stiffness (≥ 10 m/s)
Metabolically healthy
Metabolically unhealthy
0
1
2
3
4
5
6
7
8
9
10
Hazard Ratio (HR)

I

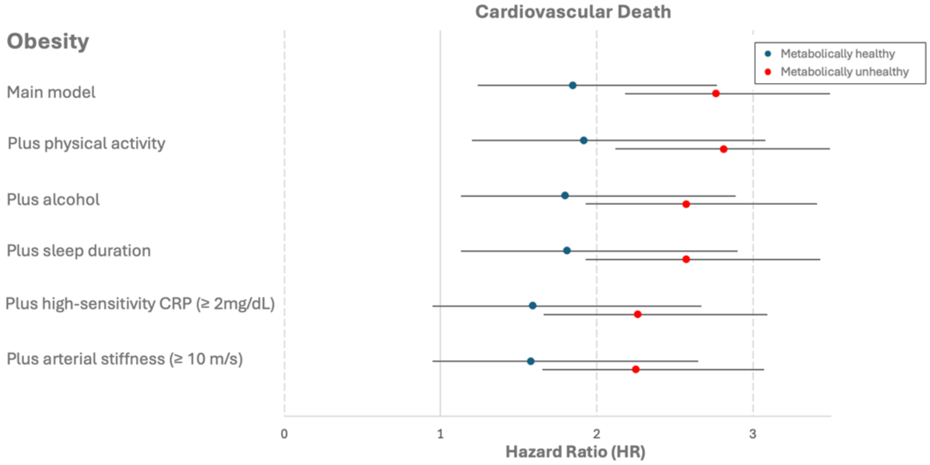
Cardiovascular Death
Obesity
Main model
Plus physical activity
Plus alcohol
Plus sleep duration
Plus high-sensitivity CRP (≥ 2mg/dL)
Plus arterial stiffness (≥ 10 m/s)
Metabolically healthy
Metabolically unhealthy
0
1
2
3
Hazard Ratio (HR)

J

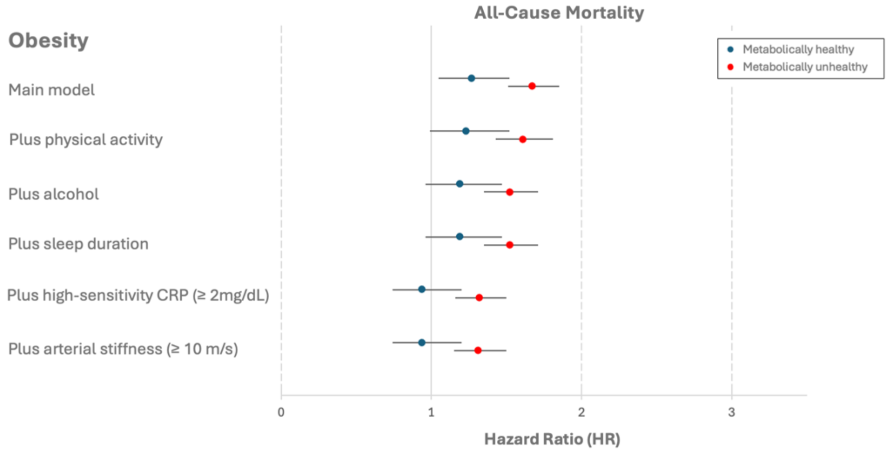
All-Cause Mortality
Obesity
Main model
Plus physical activity
Plus alcohol
Plus sleep duration
Plus high-sensitivity CRP (≥ 2mg/dL)
Plus arterial stiffness (≥ 10 m/s)
Metabolically healthy
Metabolically unhealthy
0
1
2
3
Hazard Ratio (HR)

Figure S10: Sensitivity analysis of the relationship between unhealthy metabolic phenotype and BMI after adjusting for lifestyle and biological factors in Men with the risk of atherosclerotic cardiovascular disease **(A)**, coronary heart disease **(B)**, ischaemic stroke **(C)**, peripheral artery disease **(D)**, myocardial infarction **(E)**, heart failure **(F)**, metabolic dysfunction-associated steatotic liver disease **(G)**, end stage renal disease **(H)**, cardiovascular death **(I)** and all-cause mortality **(J)**.

¥ Main model adjusted for age, smoking status, ethnicity, and Townsend deprivation quintiles.
¥ Reference group was metabolically healthy participants with normal BMI.
¥ The cox-regression model generated above includes all six main metabolic categories. Only three metabolic categories representing unhealthy metabolic profile were included here to allow comparison.

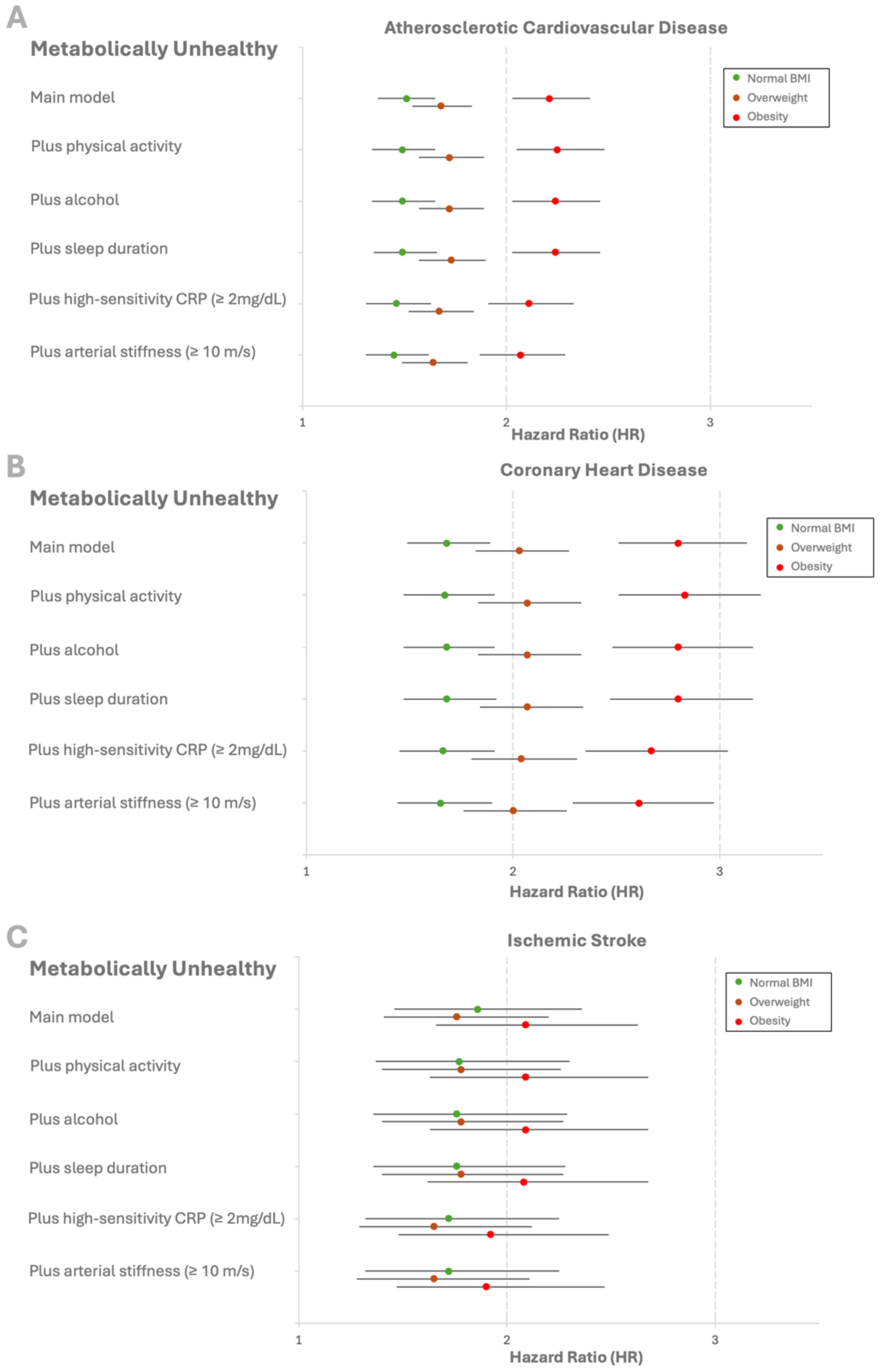

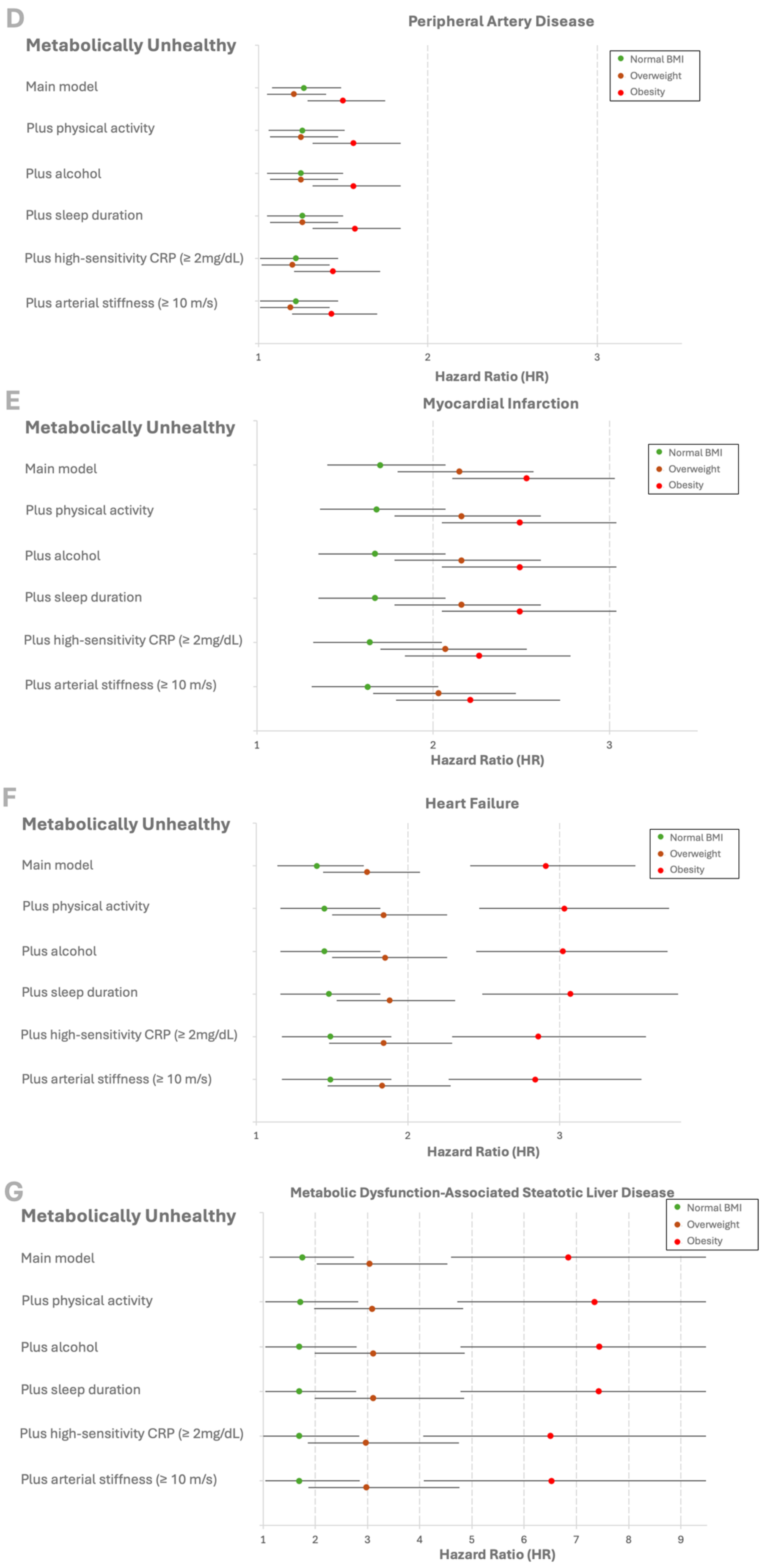
D
Peripheral Artery Disease
Metabolically Unhealthy
Main model
Plus physical activity
Plus alcohol
Plus sleep duration
Plus high-sensitivity CRP (≥ 2mg/dL)
Plus arterial stiffness (≥ 10 m/s)
Normal BMI
Overweight
Obesity
1
2
3
Hazard Ratio (HR)
E
Myocardial Infarction
Metabolically Unhealthy
Main model
Plus physical activity
Plus alcohol
Plus sleep duration
Plus high-sensitivity CRP (≥ 2mg/dL)
Plus arterial stiffness (≥ 10 m/s)
Normal BMI
Overweight
Obesity
1
2
3
Hazard Ratio (HR)
F
Heart Failure
Metabolically Unhealthy
Main model
Plus physical activity
Plus alcohol
Plus sleep duration
Plus high-sensitivity CRP (≥ 2mg/dL)
Plus arterial stiffness (≥ 10 m/s)
Normal BMI
Overweight
Obesity
1
2
3
Hazard Ratio (HR)
G
Metabolic Dysfunction-Associated Steatotic Liver Disease
Metabolically Unhealthy
Main model
Plus physical activity
Plus alcohol
Plus sleep duration
Plus high-sensitivity CRP (≥ 2mg/dL)
Plus arterial stiffness (≥ 10 m/s)
Normal BMI
Overweight
Obesity
1
2
3
4
5
6
7
8
9
Hazard Ratio (HR)

H

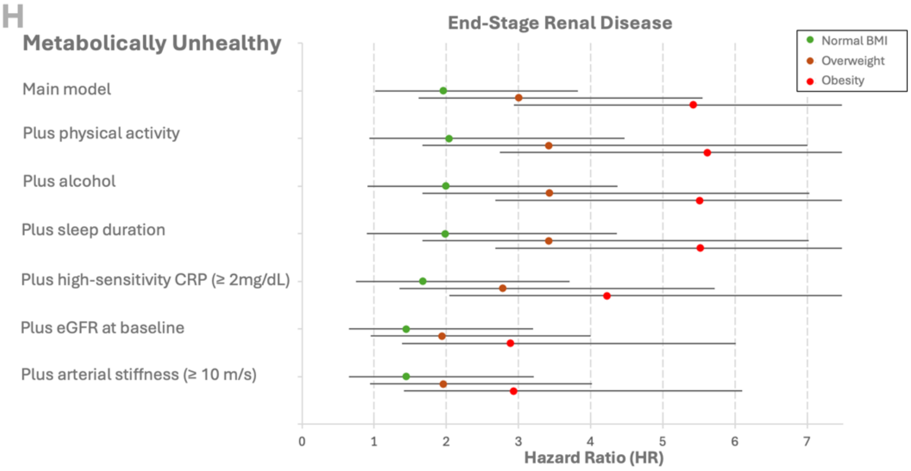

Metabolically Unhealthy
End-Stage Renal Disease
Normal BMI
Overweight
Obesity
Main model
Plus physical activity
Plus alcohol
Plus sleep duration
Plus high-sensitivity CRP (≥ 2mg/dL)
Plus eGFR at baseline
Plus arterial stiffness (≥ 10 m/s)
0
1
2
3
4
5
6
7
Hazard Ratio (HR)

I

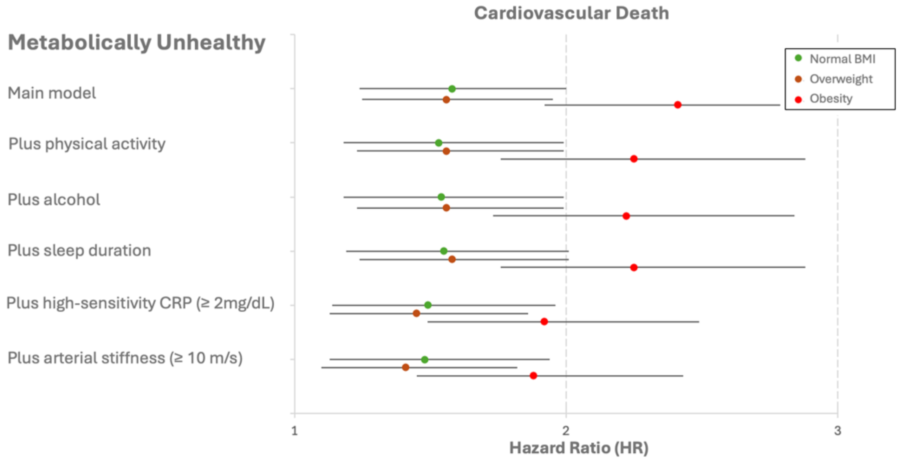

Metabolically Unhealthy
Cardiovascular Death
Normal BMI
Overweight
Obesity
Main model
Plus physical activity
Plus alcohol
Plus sleep duration
Plus high-sensitivity CRP (≥ 2mg/dL)
Plus arterial stiffness (≥ 10 m/s)
1
2
3
Hazard Ratio (HR)

J

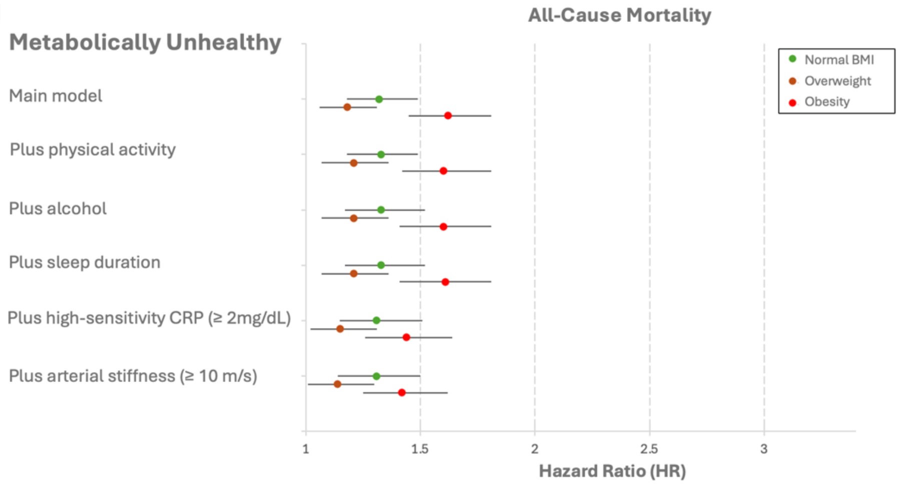

Metabolically Unhealthy
All-Cause Mortality
Normal BMI
Overweight
Obesity
Main model
Plus physical activity
Plus alcohol
Plus sleep duration
Plus high-sensitivity CRP (≥ 2mg/dL)
Plus arterial stiffness (≥ 10 m/s)
1
1.5
2
2.5
3
Hazard Ratio (HR)

Figure S11: Sensitivity analysis of the relationship between unhealthy metabolic phenotype and BMI after adjusting for lifestyle and biological factors in Women with the risk of atherosclerotic cardiovascular disease **(A)**, coronary heart disease **(B)**, ischaemic stroke **(C)**, peripheral artery disease **(D)**, myocardial infarction **(E)**, heart failure **(F)**, metabolic dysfunction-associated steatotic liver disease **(G)**, end stage renal disease **(H)**, cardiovascular death **(I)** and all-cause mortality **(J)**.

¥ Main model adjusted for age, smoking status, ethnicity, and Townsend deprivation quintiles.
¥ Reference group was metabolically healthy participants with normal BMI.
¥ The cox-regression model generated above includes all six main metabolic categories. Only three metabolic categories representing unhealthy metabolic profile were included here to allow comparison.

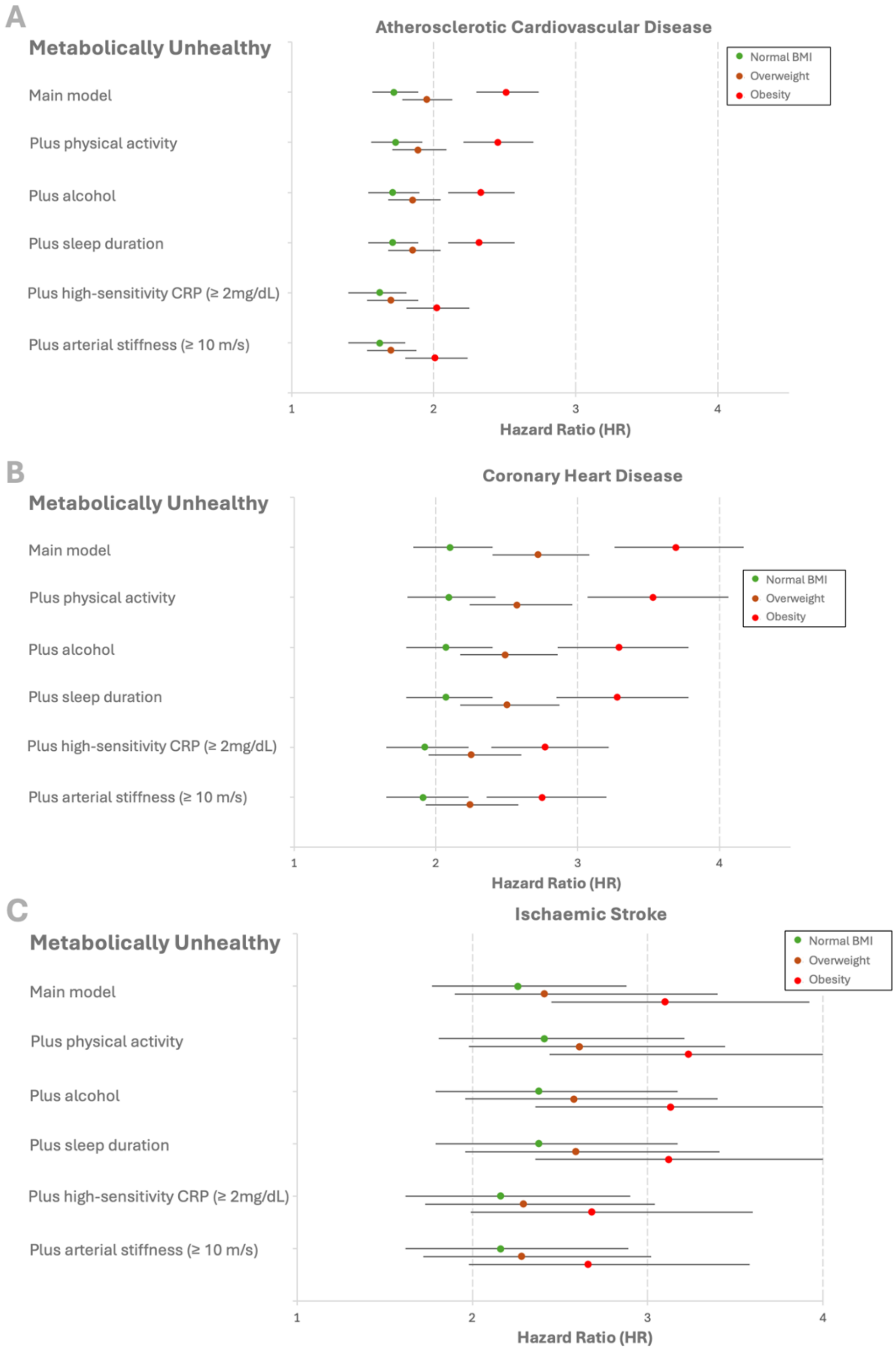

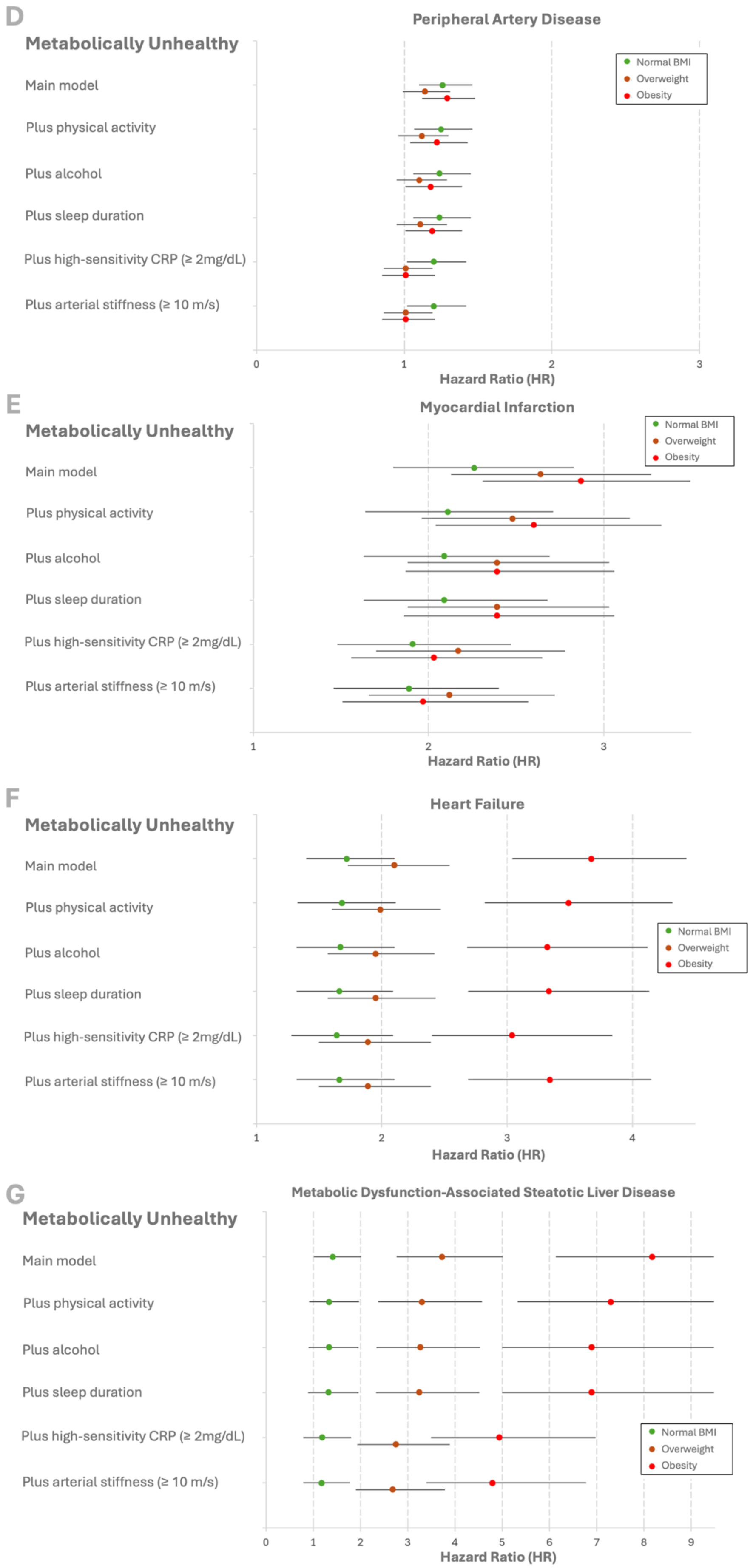
D
Peripheral Artery Disease
Metabolically Unhealthy
Main model
Plus physical activity
Plus alcohol
Plus sleep duration
Plus high-sensitivity CRP (≥ 2mg/dL)
Plus arterial stiffness (≥ 10 m/s)
Normal BMI
Overweight
Obesity
0
1
2
3
Hazard Ratio (HR)
E
Myocardial Infarction
Metabolically Unhealthy
Main model
Plus physical activity
Plus alcohol
Plus sleep duration
Plus high-sensitivity CRP (≥ 2mg/dL)
Plus arterial stiffness (≥ 10 m/s)
Normal BMI
Overweight
Obesity
1
2
3
Hazard Ratio (HR)
F
Heart Failure
Metabolically Unhealthy
Main model
Plus physical activity
Plus alcohol
Plus sleep duration
Plus high-sensitivity CRP (≥ 2mg/dL)
Plus arterial stiffness (≥ 10 m/s)
Normal BMI
Overweight
Obesity
1
2
3
4
Hazard Ratio (HR)
G
Metabolic Dysfunction-Associated Steatotic Liver Disease
Metabolically Unhealthy
Main model
Plus physical activity
Plus alcohol
Plus sleep duration
Plus high-sensitivity CRP (≥ 2mg/dL)
Plus arterial stiffness (≥ 10 m/s)
Normal BMI
Overweight
Obesity
0
1
2
3
4
5
6
7
8
9
Hazard Ratio (HR)

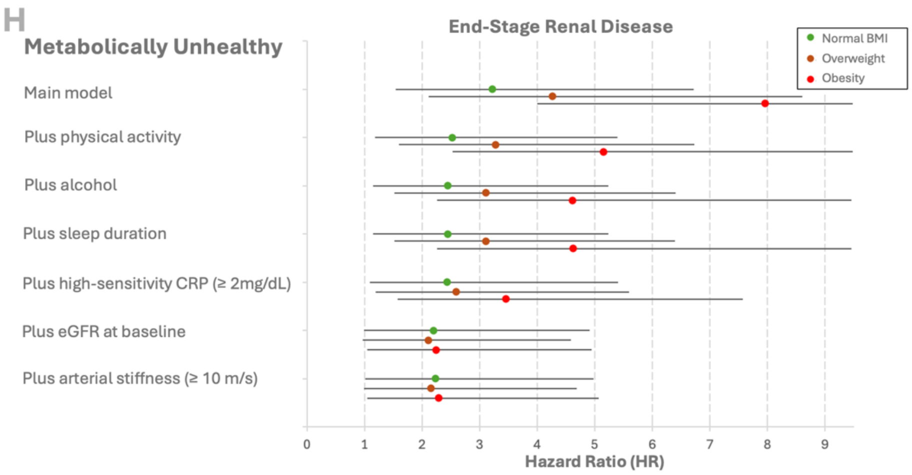
H
Metabolically Unhealthy
End-Stage Renal Disease
Normal BMI
Overweight
Obesity
Main model
Plus physical activity
Plus alcohol
Plus sleep duration
Plus high-sensitivity CRP (≥ 2mg/dL)
Plus eGFR at baseline
Plus arterial stiffness (≥ 10 m/s)
0
1
2
3
4
5
6
7
8
9
Hazard Ratio (HR)

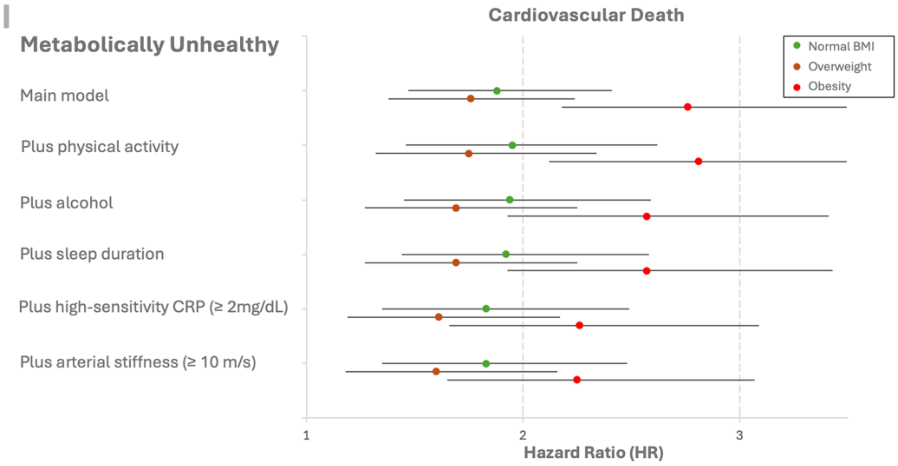
I
Metabolically Unhealthy
Cardiovascular Death
Normal BMI
Overweight
Obesity
Main model
Plus physical activity
Plus alcohol
Plus sleep duration
Plus high-sensitivity CRP (≥ 2mg/dL)
Plus arterial stiffness (≥ 10 m/s)
1
2
3
Hazard Ratio (HR)

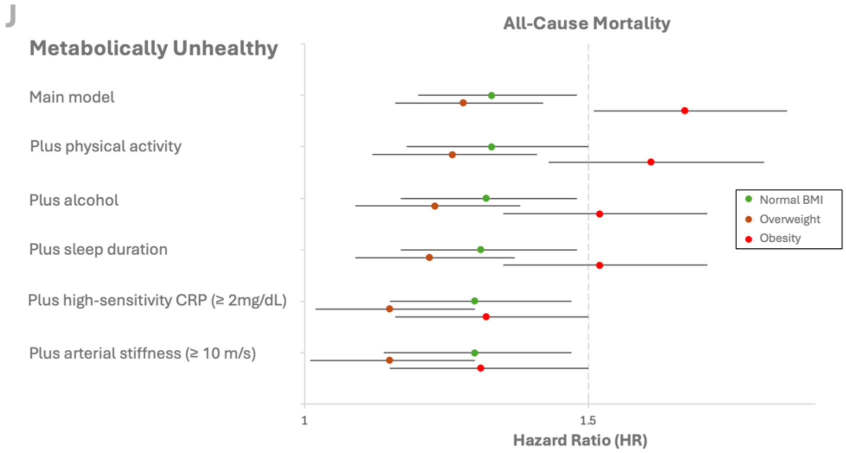
J
Metabolically Unhealthy
All-Cause Mortality
Main model
Plus physical activity
Plus alcohol
Plus sleep duration
Plus high-sensitivity CRP (≥ 2mg/dL)
Plus arterial stiffness (≥ 10 m/s)
Normal BMI
Overweight
Obesity
1
1.5
Hazard Ratio (HR)

Figure S12: Mediation analysis of the elevated high-sensitivity CRP (≥2 mg/L) and metabolically healthy obesity phenotype in Men on the risk of atherosclerotic cardiovascular disease **(A)**, coronary heart disease **(B)**, heart failure **(C)**, metabolic dysfunction-associated steatotic liver disease **(D)**, and all-cause mortality **(E)**. Mediation analysis between the proportion effect of elevated hsCRP in the relationship between metabolically healthy obesity with outcomes.

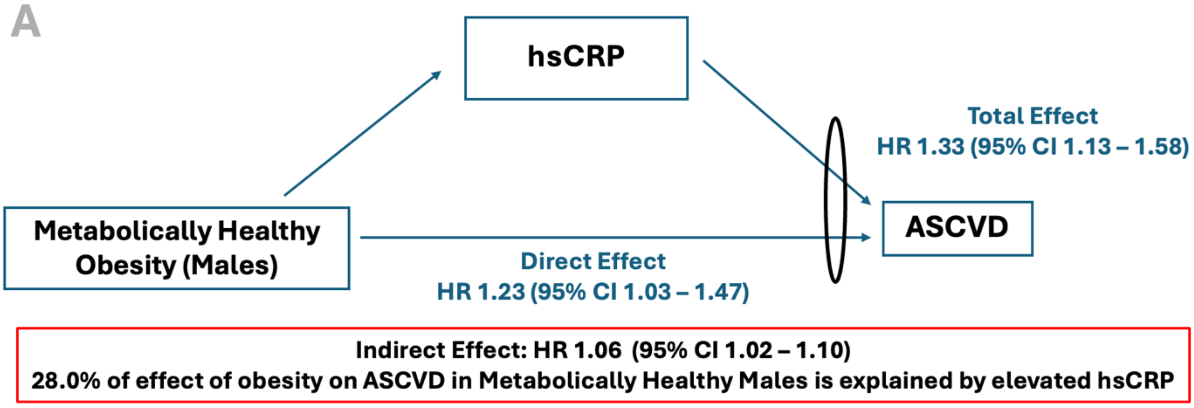

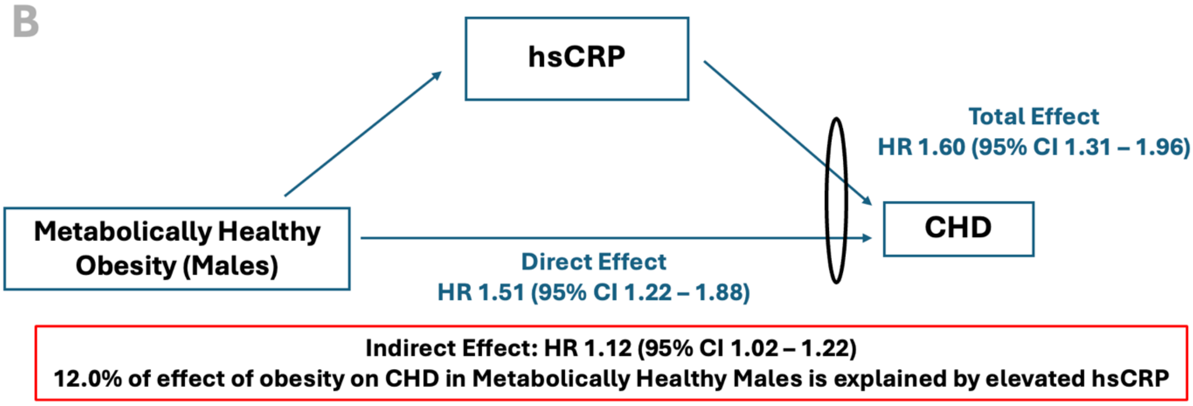

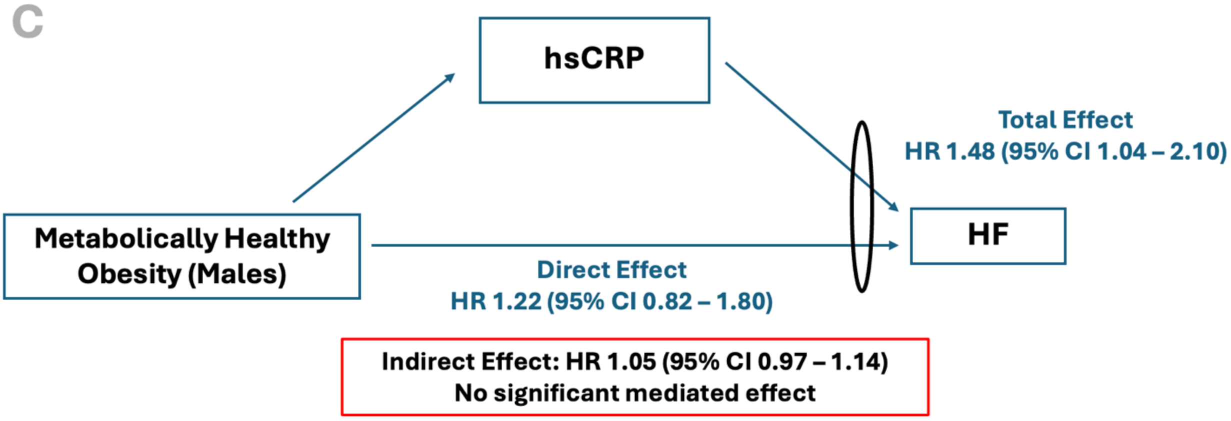

D

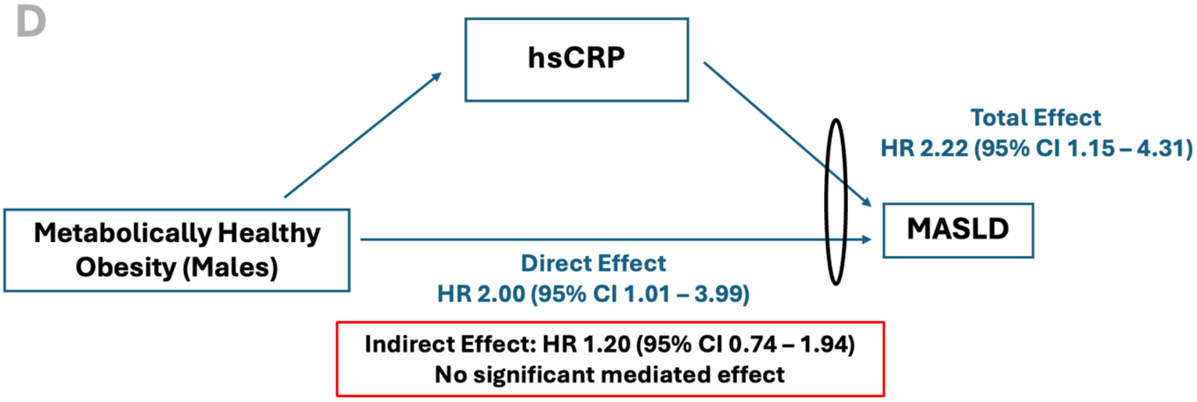
hsCRP
Total Effect
HR 2.22 (95% CI 1.15 – 4.31)
MASLD
Metabolically Healthy Obesity (Males)
Direct Effect
HR 2.00 (95% CI 1.01 – 3.99)
Indirect Effect: HR 1.20 (95% CI 0.74 – 1.94)
No significant mediated effect

E

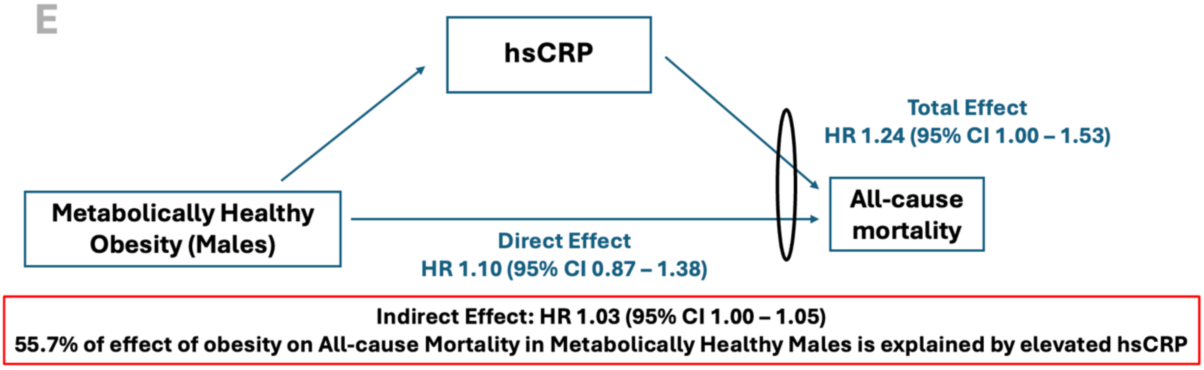
hsCRP
Total Effect
HR 1.24 (95% CI 1.00 – 1.53)
All-cause mortality
Metabolically Healthy Obesity (Males)
Direct Effect
HR 1.10 (95% CI 0.87 – 1.38)
Indirect Effect: HR 1.03 (95% CI 1.00 – 1.05)
55.7% of effect of obesity on All-cause Mortality in Metabolically Healthy Males is explained by elevated hsCRP

Figure S13: Mediation analysis of the elevated high-sensitivity CRP (≥2 mg/L) and metabolically healthy obesity phenotype in Women on the risk of atherosclerotic cardiovascular disease **(A)**, coronary heart disease **(B)**, heart failure **(C)**, metabolic dysfunction-associated steatotic liver disease **(D)**, cardiovascular death **(E)**, and all-cause mortality **(F)**.

Mediation analysis between the proportion effect of elevated hsCRP in the relationship between metabolically healthy obesity with outcomes.

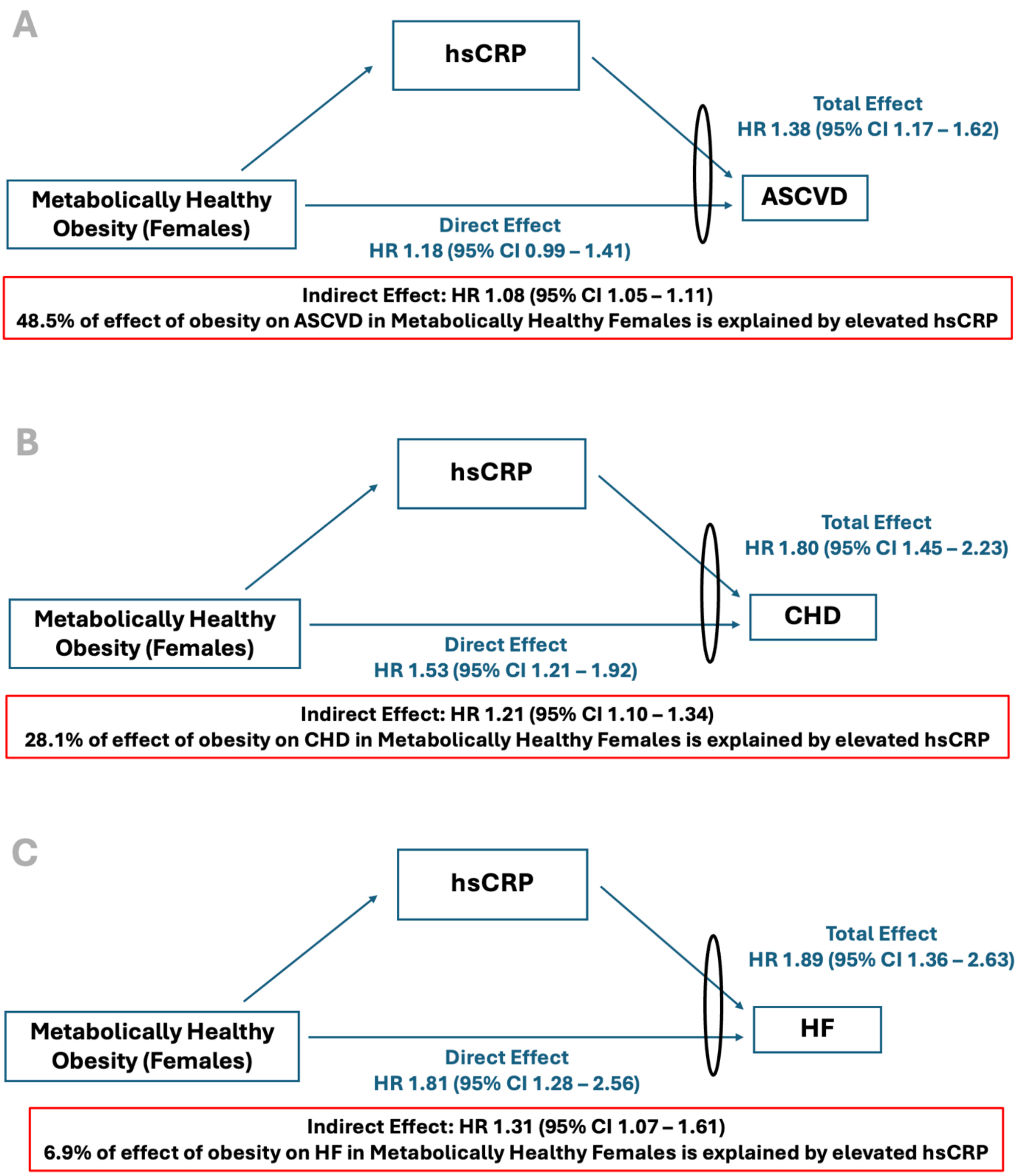

D

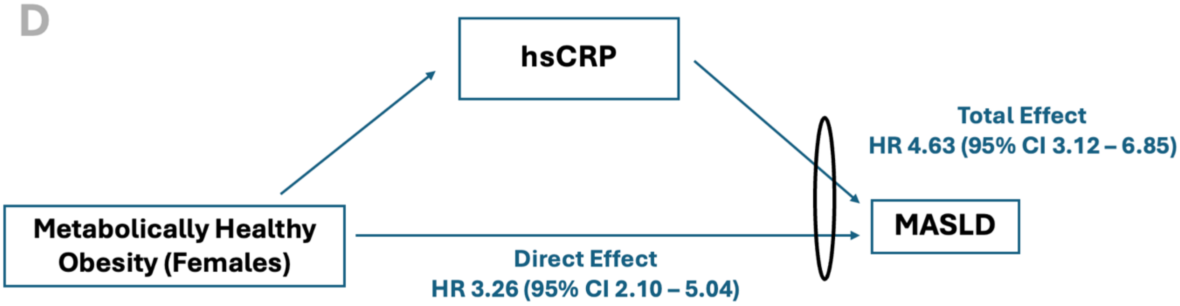

Indirect Effect: HR 1.72 (95% CI 1.03 – 2.88)
22.9% of effect of obesity on MASLD in Metabolically Healthy Females is explained by elevated hsCRP

E

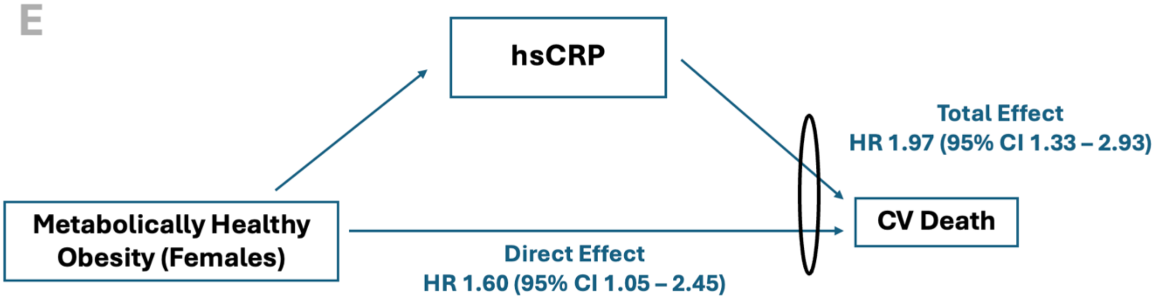

Indirect Effect: HR 1.24 (95% CI 1.02 – 1.52)
30.6% of effect of obesity on CV Death in Metabolically Healthy Females is explained by elevated hsCRP

F

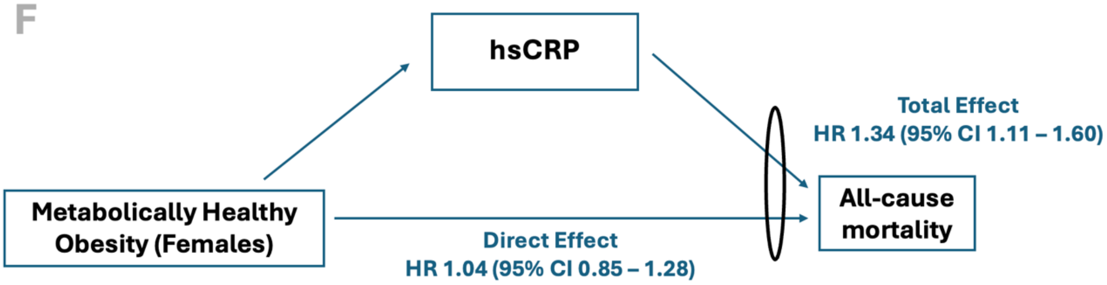

Indirect Effect: HR 1.02 (95% CI 1.01 – 1.03)
85.9% of effect of obesity on All-cause Mortality in Metabolically Healthy Females is explained by elevated hsCRP

Figure S14: Mediation analysis of the elevated high-sensitivity CRP (≥2 mg/L) and metabolically unhealthy obesity phenotype in Men on the risk of atherosclerotic cardiovascular disease **(A)**, coronary heart disease **(B)**, ischaemic stroke **(C),** peripheral artery disease **(D)**, heart failure **(E)**, metabolic dysfunction-associated steatotic liver disease **(F)**, end stage renal disease **(G)**, cardiovascular death **(H)** and all-cause mortality **(I)**.
Mediation analysis between the proportion effect of elevated hsCRP in the relationship between metabolically healthy obesity with outcomes.

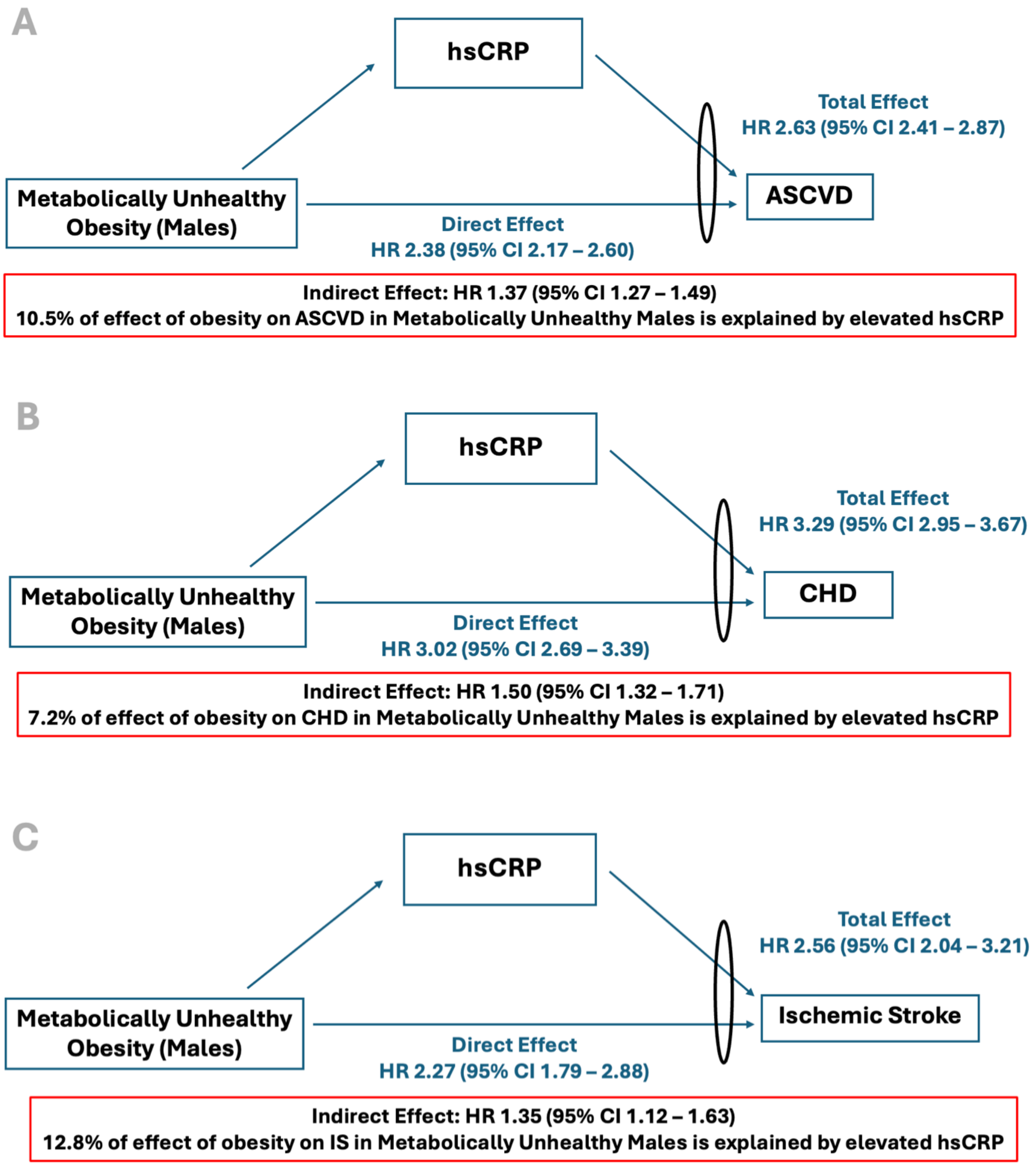

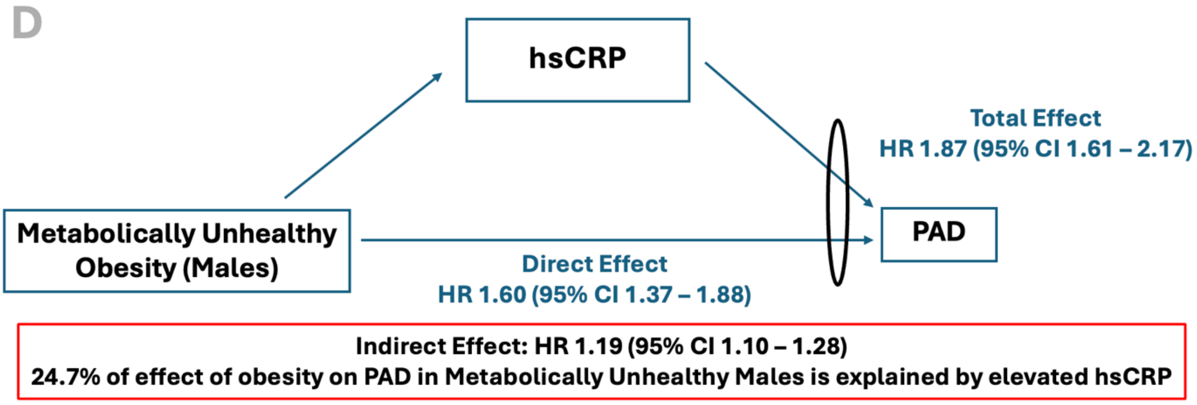
D
hsCRP
Total Effect
HR 1.87 (95% CI 1.61 – 2.17)
Metabolically Unhealthy Obesity (Males)
PAD
Direct Effect
HR 1.60 (95% CI 1.37 – 1.88)
Indirect Effect: HR 1.19 (95% CI 1.10 – 1.28)
24.7% of effect of obesity on PAD in Metabolically Unhealthy Males is explained by elevated hsCRP

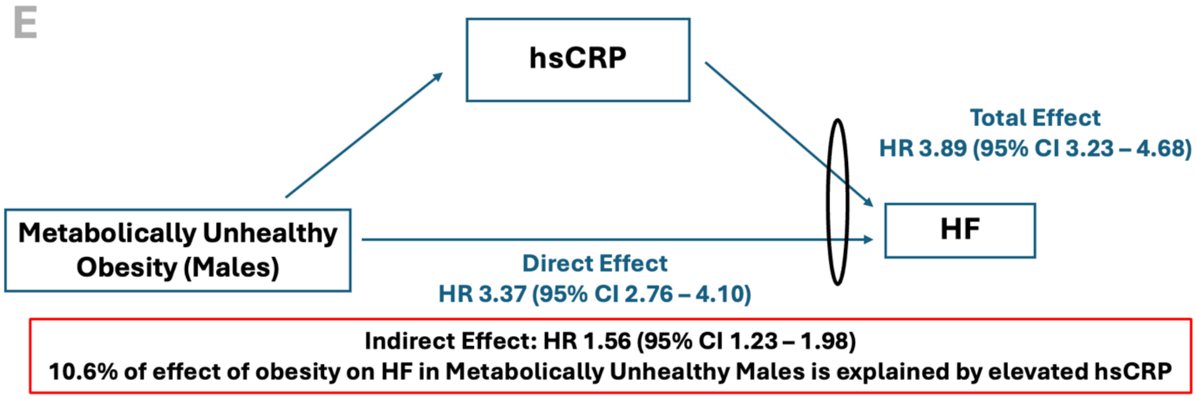
E
hsCRP
Total Effect
HR 3.89 (95% CI 3.23 – 4.68)
Metabolically Unhealthy Obesity (Males)
HF
Direct Effect
HR 3.37 (95% CI 2.76 – 4.10)
Indirect Effect: HR 1.56 (95% CI 1.23 – 1.98)
10.6% of effect of obesity on HF in Metabolically Unhealthy Males is explained by elevated hsCRP

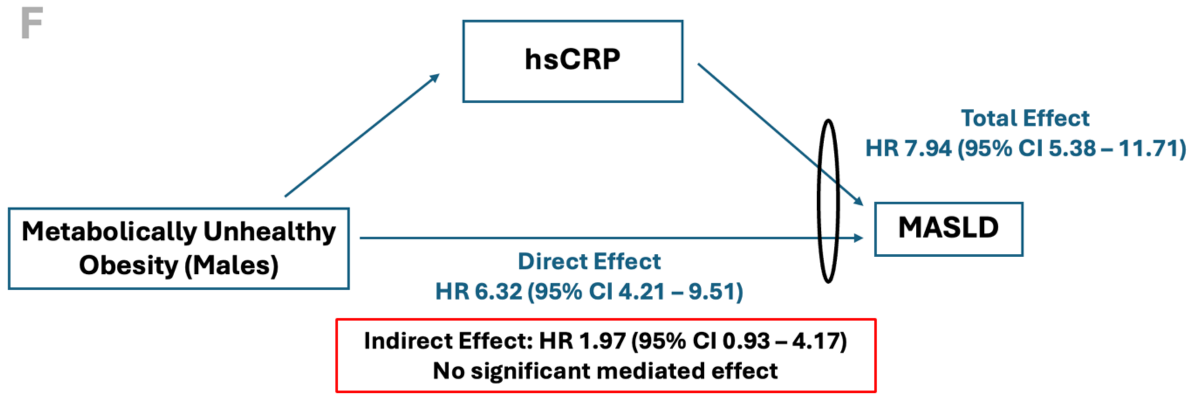
F
hsCRP
Total Effect
HR 7.94 (95% CI 5.38 – 11.71)
Metabolically Unhealthy Obesity (Males)
MASLD
Direct Effect
HR 6.32 (95% CI 4.21 – 9.51)
Indirect Effect: HR 1.97 (95% CI 0.93 – 4.17)
No significant mediated effect

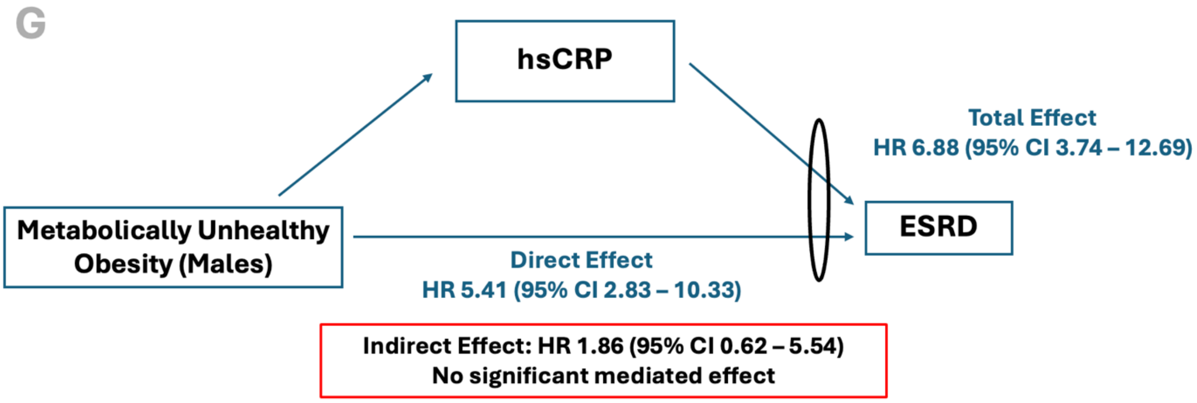
G
hsCRP
Total Effect
HR 6.88 (95% CI 3.74 – 12.69)
Metabolically Unhealthy Obesity (Males)
ESRD
Direct Effect
HR 5.41 (95% CI 2.83 – 10.33)
Indirect Effect: HR 1.86 (95% CI 0.62 – 5.54)
No significant mediated effect

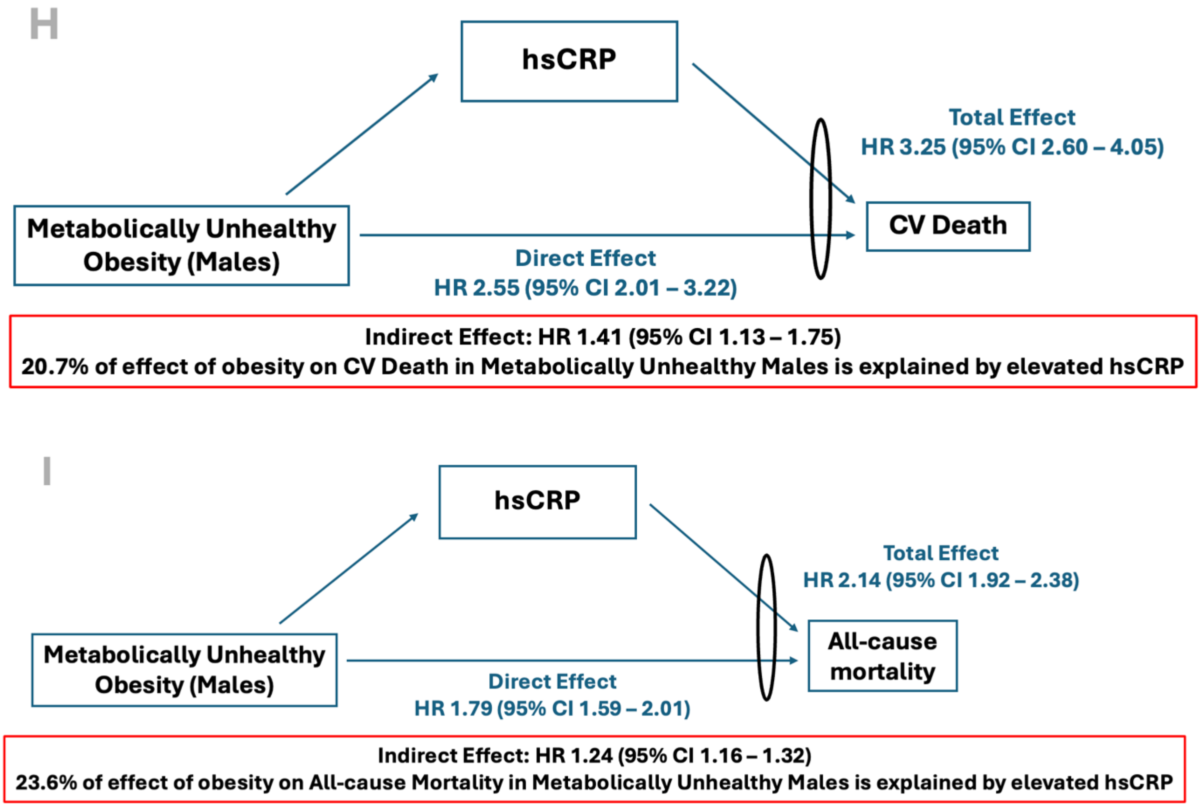
H
hsCRP
Total Effect
HR 3.25 (95% CI 2.60 – 4.05)
Metabolically Unhealthy Obesity (Males)
CV Death
Direct Effect
HR 2.55 (95% CI 2.01 – 3.22)
Indirect Effect: HR 1.41 (95% CI 1.13 – 1.75)
20.7% of effect of obesity on CV Death in Metabolically Unhealthy Males is explained by elevated hsCRP
I
hsCRP
Total Effect
HR 2.14 (95% CI 1.92 – 2.38)
Metabolically Unhealthy Obesity (Males)
All-cause mortality
Direct Effect
HR 1.79 (95% CI 1.59 – 2.01)
Indirect Effect: HR 1.24 (95% CI 1.16 – 1.32)
23.6% of effect of obesity on All-cause Mortality in Metabolically Unhealthy Males is explained by elevated hsCRP

Figure S15: Mediation analysis of the elevated high-sensitivity CRP (≥2 mg/L) and metabolically unhealthy obesity phenotype in Women on the risk of atherosclerotic cardiovascular disease **(A)**, coronary heart disease **(B)**, ischaemic stroke **(C)**, peripheral artery disease **(D)**, heart failure **(E)**, metabolic dysfunction-associated steatotic liver disease **(F)**, end stage renal disease **(G)**, cardiovascular death **(H)** and all-cause mortality **(I)**.
Mediation analysis between the proportion effect of elevated hsCRP in the relationship between metabolically healthy obesity with outcomes.

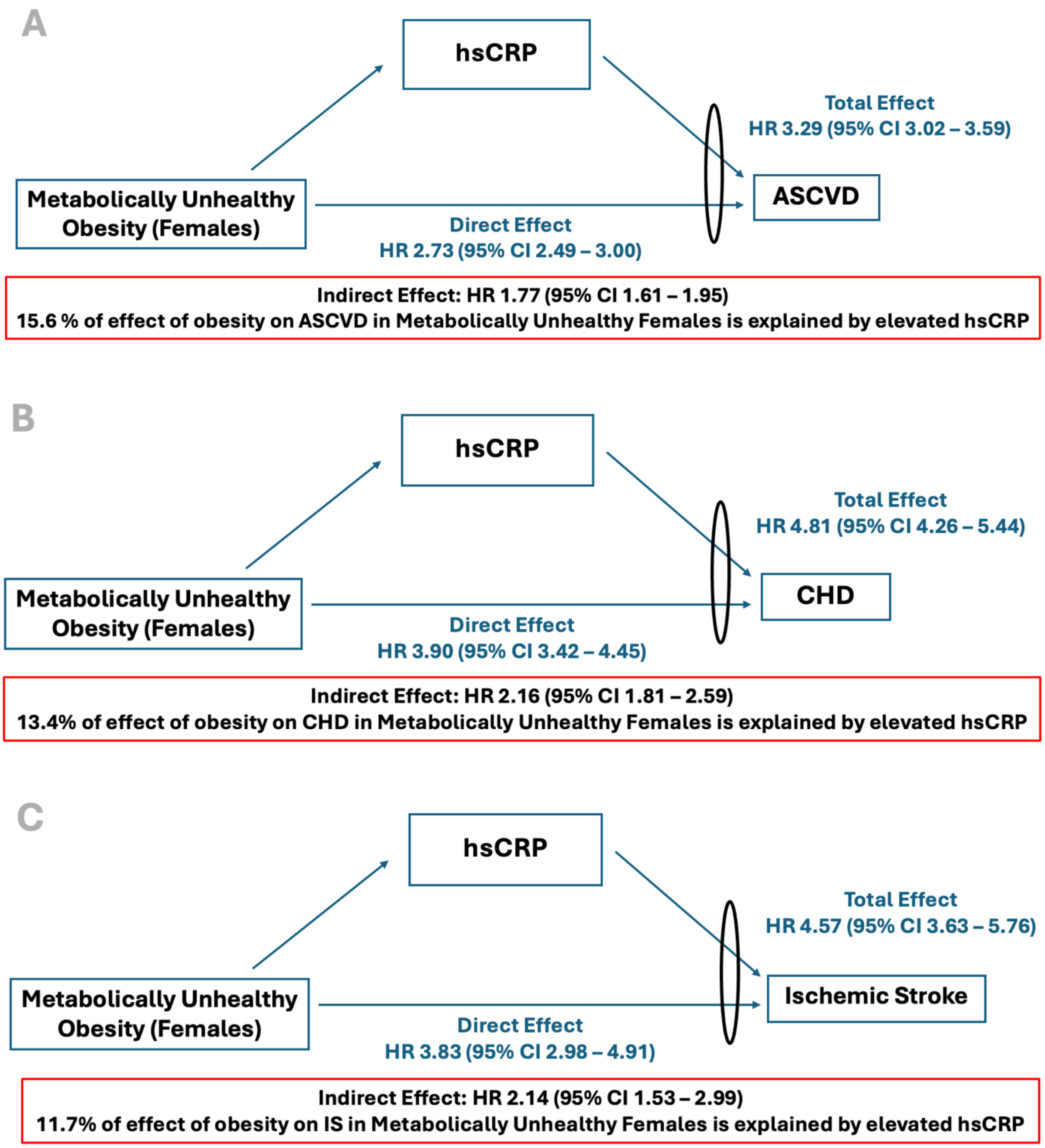

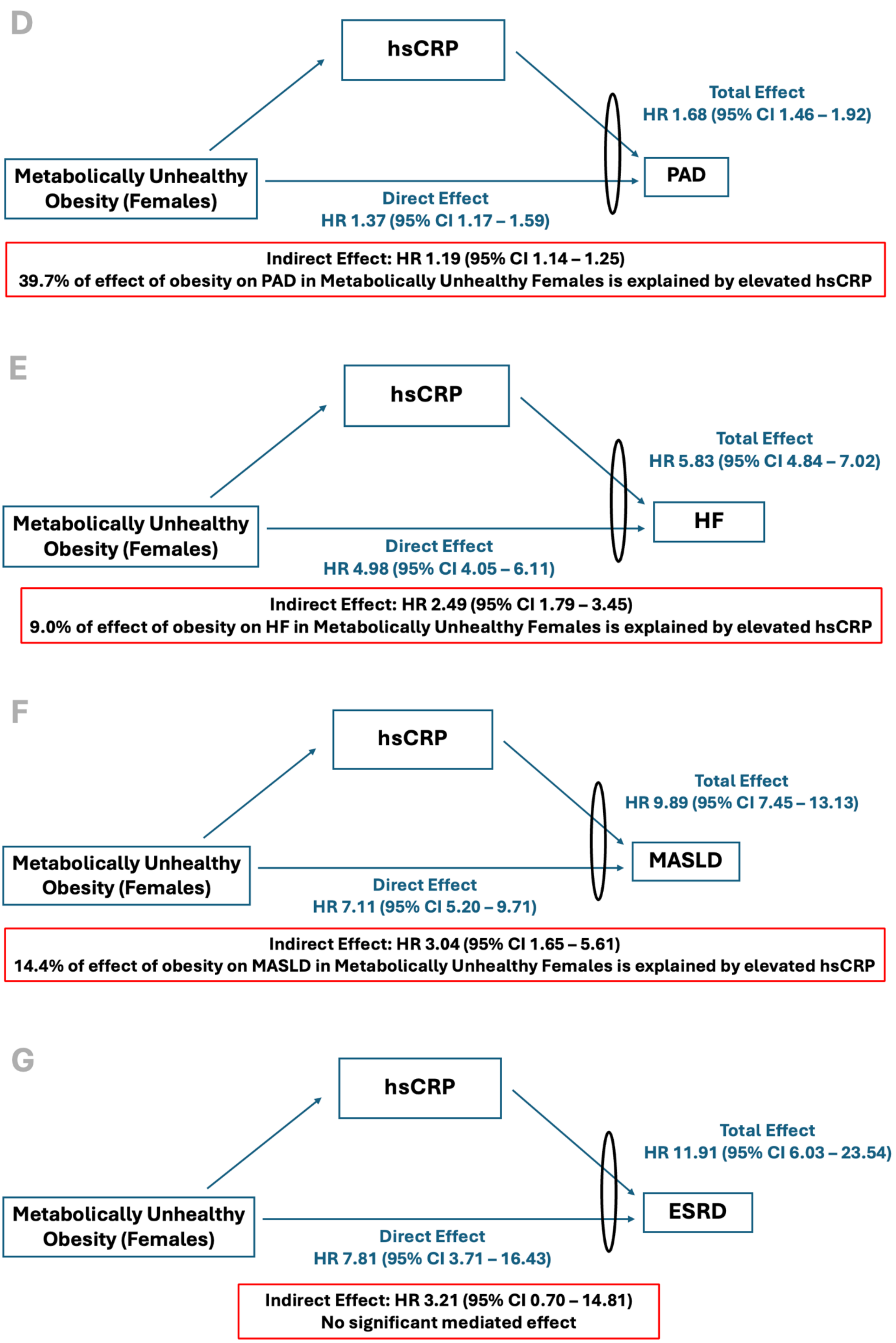
D
hsCRP
Total Effect
HR 1.68 (95% CI 1.46 – 1.92)
Metabolically Unhealthy Obesity (Females)
PAD
Direct Effect
HR 1.37 (95% CI 1.17 – 1.59)
Indirect Effect: HR 1.19 (95% CI 1.14 – 1.25)
39.7% of effect of obesity on PAD in Metabolically Unhealthy Females is explained by elevated hsCRP
E
hsCRP
Total Effect
HR 5.83 (95% CI 4.84 – 7.02)
Metabolically Unhealthy Obesity (Females)
HF
Direct Effect
HR 4.98 (95% CI 4.05 – 6.11)
Indirect Effect: HR 2.49 (95% CI 1.79 – 3.45)
9.0% of effect of obesity on HF in Metabolically Unhealthy Females is explained by elevated hsCRP
F
hsCRP
Total Effect
HR 9.89 (95% CI 7.45 – 13.13)
Metabolically Unhealthy Obesity (Females)
MASLD
Direct Effect
HR 7.11 (95% CI 5.20 – 9.71)
Indirect Effect: HR 3.04 (95% CI 1.65 – 5.61)
14.4% of effect of obesity on MASLD in Metabolically Unhealthy Females is explained by elevated hsCRP
G
hsCRP
Total Effect
HR 11.91 (95% CI 6.03 – 23.54)
Metabolically Unhealthy Obesity (Females)
ESRD
Direct Effect
HR 7.81 (95% CI 3.71 – 16.43)
Indirect Effect: HR 3.21 (95% CI 0.70 – 14.81)
No significant mediated effect

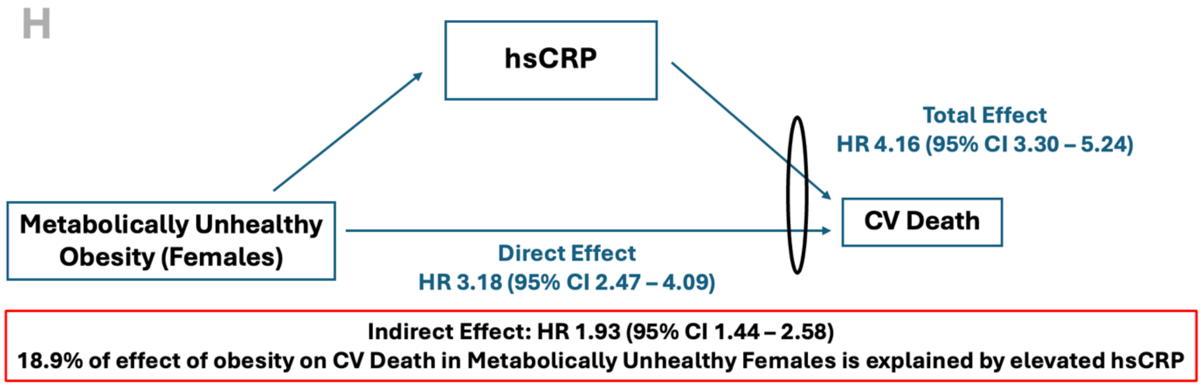
H
hsCRP
Total Effect
HR 4.16 (95% CI 3.30 – 5.24)
Metabolically Unhealthy Obesity (Females)
CV Death
Direct Effect
HR 3.18 (95% CI 2.47 – 4.09)
Indirect Effect: HR 1.93 (95% CI 1.44 – 2.58)
18.9% of effect of obesity on CV Death in Metabolically Unhealthy Females is explained by elevated hsCRP

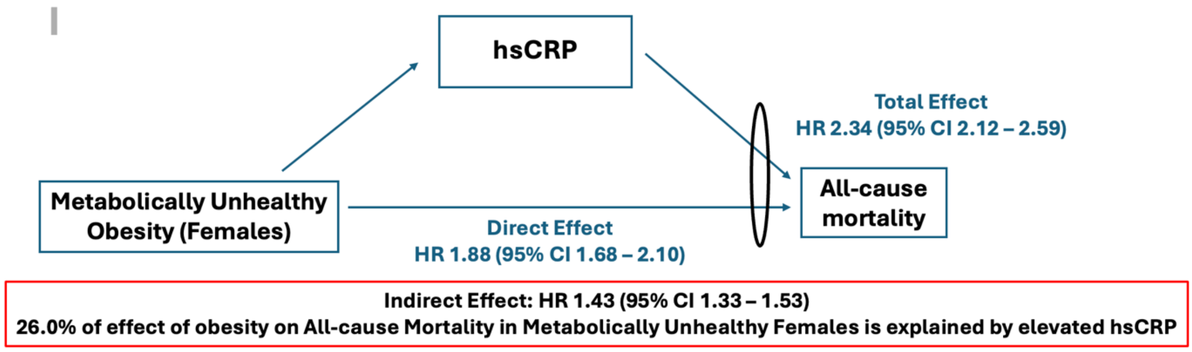
I
hsCRP
Total Effect
HR 2.34 (95% CI 2.12 – 2.59)
Metabolically Unhealthy Obesity (Females)
All-cause mortality
Direct Effect
HR 1.88 (95% CI 1.68 – 2.10)
Indirect Effect: HR 1.43 (95% CI 1.33 – 1.53)
26.0% of effect of obesity on All-cause Mortality in Metabolically Unhealthy Females is explained by elevated hsCRP

References

1. McEvoy JW, McCarthy CP, Bruno RM, et al. 2024 ESC Guidelines for the management of elevated blood pressure and hypertension. *Eur Heart J* 2024; 45: 3912-4018.

2. Cosentino F, Grant PJ, Aboyans V, et al. 2019 ESC Guidelines on diabetes, pre-diabetes, and cardiovascular diseases developed in collaboration with the EASD. *Eur Heart J* 2020; 41: 255-323.

3. Mach F, Baigent C, Catapano AL, et al. 2019 ESC/EAS Guidelines for the management of dyslipidaemias: lipid modification to reduce cardiovascular risk. *Eur Heart J* 2020; 41: 111-188.

4. Grundy SM. Hypertriglyceridemia, insulin resistance, and the metabolic syndrome. *Am J Cardiol* 1999; 83: 25f-29f.

5. Ma M, Liu H, Yu J, et al. Triglyceride is independently correlated with insulin resistance and islet beta cell function: a study in population with different glucose and lipid metabolism states. *Lipids Health Dis* 2020; 19: 121.

6. Schols AM, Broekhuizen R, Weling-Scheepers CA, et al. Body composition and mortality in chronic obstructive pulmonary disease. *Am J Clin Nutr* 2005; 82: 53-59.

7. Hallin R, Gudmundsson G, Suppli Ulrik C, et al. Nutritional status and long-term mortality in hospitalised patients with chronic obstructive pulmonary disease (COPD). *Respir Med* 2007; 101: 1954-1960.

8. Kroenke CH, Neugebauer R, Meyerhardt J, et al. Analysis of Body Mass Index and Mortality in Patients With Colorectal Cancer Using Causal Diagrams. *JAMA Oncol* 2016; 2: 1137-1145.

9. Moon HG, Han W and Noh DY. Underweight and breast cancer recurrence and death: a report from the Korean Breast Cancer Society. *J Clin Oncol* 2009; 27: 5899-5905.
